# Blue light photoredox-catalysed acetalation of alkynyl bromides†

**DOI:** 10.1039/c9ra06596b

**Published:** 2019-11-06

**Authors:** Xue-Li Lyu, Shi-Sheng Huang, Hong-Jian Song, Yu-Xiu Liu, Qing-Min Wang

**Affiliations:** State Key Laboratory of Elemento-Organic Chemistry, Research Institute of Elemento-Organic Chemistry, College of Chemistry, Nankai University Tianjin 300071 People's Republic of China wangqm@nankai.edu.cn; Collaborative Innovation Center of Chemical Science and Engineering (Tianjin) Tianjin 300071 People's Republic of China

## Abstract

Herein, we report an organo-photoredox-based protocol using 2,2-diethoxyacetic acid as the acetal source to achieve acetalation of alkynyl bromides to afford various alkynyl acetal products. In addition to arylethynyl bromides, substrates bearing heteroaryl rings (thiophene, pyridine, and indole) smoothly gave the corresponding acetalation products. This mild protocol has potential utility for the synthesis of aldehydes by further protonization.

## Abstract 2

Herein, we report an organo-photoredox-based protocol using 2,2-diethoxyacetic acid as the acetal source to achieve acetalation of alkynyl bromides to afford various alkynyl acetal products.

## Graphical abstract

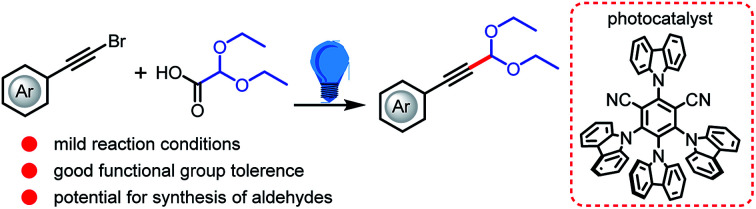

Acetals play a key role in several biological interactions,^1,2^ and they are widespread in natural products obtained from various sources, including insects, marine organisms, fungi, plants, and microbes.^2^ In addition, reports of acetal-containing drugs have recently appeared in the literature,^3^ and acetals are frequently used to protect carbonyl groups during complex synthesis^4^ (Fig. 1). Therefore, the development of new methods for the introduction of acetal moieties is highly desirable, and environmentally friendly methods are of particular interest.

**Fig. 1:**
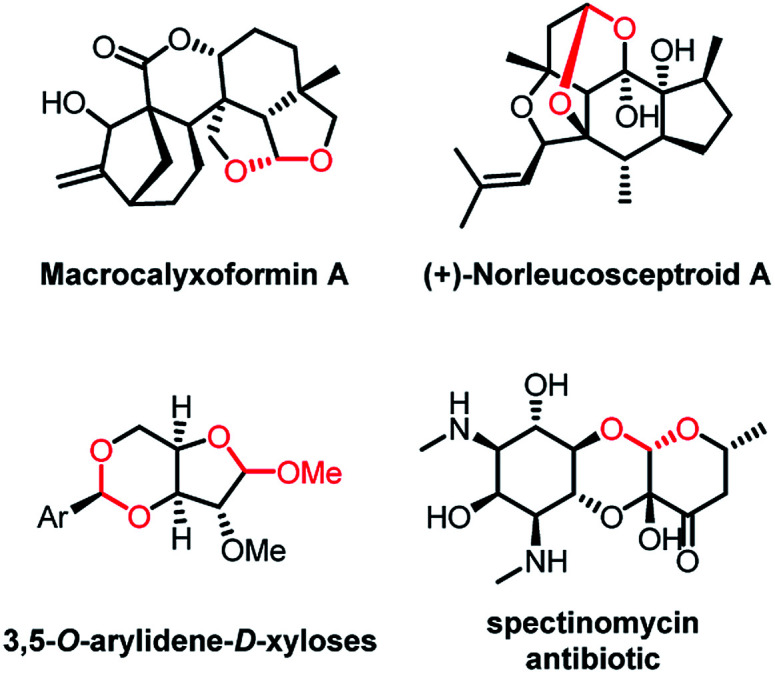
Representative compounds containing acetal moieties.

In light of the growing demand for environmentally benign synthetic methods, visible-light-induced catalysis has recently garnered substantial attention in organic synthesis owing to its mild conditions and low energy requirements.^5^ For example, photoredox-catalyzed decarboxylation of alkyl carboxylic acids has become an important method for generating alkyl radicals.^6^ During decarboxylation of carboxylic acids and their derivatives under photoredox conditions, a C-centered radical can be generated *via* two complementary pathways: (a) a reductive quenching process in which the carboxylic acid transfers an electron to the excited-state photocatalyst to generate a C-centered radical after CO_2_ extrusion^7^ and (b) an oxidative quenching process in which carboxylic acid derivatives such as *N*-(acyloxy)phthalimides accept an electron from the excited-state photocatalyst to form a C-centered radical after extrusion of CO_2_ and a phthalimide anion (Scheme 1).^8^ This photoredox-catalyzed radical decarboxylative functionalization offers a novel and efficient method for constructing C–C and C–X bonds.^9^ Recently, Xu and co-workers^10^ developed a protocol for iridium-photoredox-catalyzed decarboxylative conjugated addition reactions between glyoxylic acid acetals and Michael acceptors; theirs was the first report of the introduction of acetal moieties *via* oxidative decarboxylation.

**Scheme 1:**
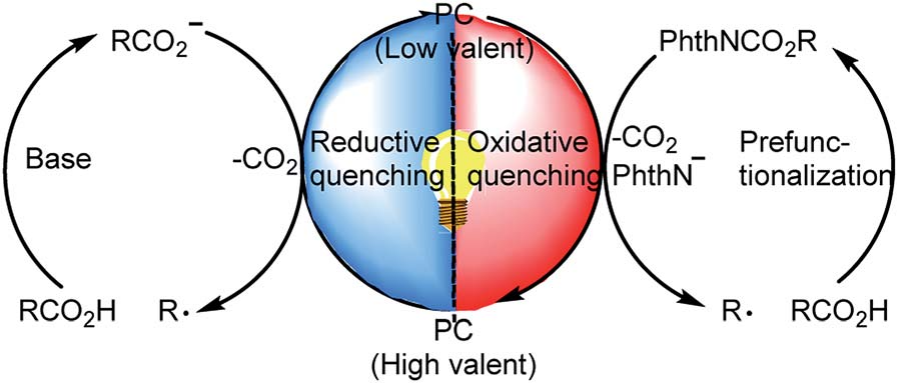
Decarboxylation of alkyl carboxylic acids and their derivatives *via* photoredox catalysis.

Herein, we report an organo-photoredox-based protocol for acetalation of alkynyl bromides, using 2,2-diethoxyacetic acid as the acetal source and 2,4,5,6-*tetra*(9*H*-carbazol-9-yl)isophthalonitrile (4CzIPN) as the photocatalyst. Aldehydes are reactive and therefore need to be protected against oxidation and reduction, and our operationally simple protocol provides direct access to protected aldehydes without producing large amounts of chemical waste. Considering its mild, metal-free conditions, the protocol has a considerable potential utility for the synthesis of alkynyl acetals.

We carried out reactions using alkynyl bromide 1g and 2,2-diethoxyacetic acid 2 as model reactants to optimize the conditions. When using 4CzIPN as a photocatalyst, irradiation of the substrates with 5 W blue LEDs at room temperature in the presence of Cs_2_CO_3_ under argon for 24 h, we delighted to obtain the desired product 3g in 57% yield (see the ESI† for details). Next, we varied the reaction parameters with the goal of optimizing the yield. Through control experiment, we found 4CzIPN, a blue LEDs, Cs_2_CO_3_, argon and Br as the leaving group were all indispensable. Even though benziodoxole-substituted alkynes are known to act as electrophilic alkyne acceptors,^11^ we found that an alkyne in which the Br atom was replaced with a benziodoxole moiety did not undergo addition of the acetal radical. By screening other photocatalysts, we found that 4CzIPN, which is a carbazole-based sensitizer reported by Zhang^12*a*^ and Adachi,^12*b*^ was optimal. The high reduction potential of photoexcited 4CzIPN (*E*_red_ [PC*/PC˙^−^] = +1.35 V *vs.* SCE)^12*a*^ makes it suitable for promoting photo-oxidative decarboxylation reactions. Ir[(dF(CF_3_)ppy)_2_(dtbbpy)]PF_6_ (*E*_red_ [Ir^III^*/Ir^II^] = +1.21 V *vs.* SCE) also catalyzed the reaction, although the yield was substantially lower than that with 4CzIPN. Two other metallaphotoredox catalysts, Ru(bpy)_3_Cl_2_·6H_2_O and Ru(bpy)_3_(PF_6_)_2_, were screened and found to be ineffective, perhaps because the low oxidation potentials (*E*_red_ [Ru^II^*/Ru^I^] = +0.77 V *vs.* SCE) of their excited states prevented them from promoting the decarboxylation reaction. Reaction in MeCN alone or DMF alone gave a lower yield than reaction in the 1 : 1 (v/v) MeCN/DMF. Finally, Cs_2_CO_3_ was a better base than Na_2_CO_3_ or K_2_CO_3_.

Using the optimal conditions, we explored the substrate scope of the reaction with respect to the alkynyl bromide and found that a broad range of functional groups were tolerated (Scheme 2). Specifically, substrates with electron-donating groups on the aromatic ring of the alkynyl bromide gave moderate yields (34–64%) of the expected products (3a–3k) upon reaction with 2. For example, reactions of phenylacetylene bromides with Me, Et, ^*n*^Pr, ^*n*^Bu, ^*t*^Bu, and Ph groups at the *para* position of the benzene ring afforded 3b–3g, respectively. Some heteroatom-containing electron-donating groups were also explored. In addition to a methoxy group (3h), a 3,4-methylenedioxy group, a 3,4-ethylenedioxy group were acceptable, and acetals 3i and 3j, respectively, were obtained in 43% yield. A phenylacetylene bromide with an amide-substituted benzene ring also reacted smoothly, giving a 49% yield of 3k. Next we examined compounds with halogen-substituted benzene rings. Phenylacetylenes with a para F or Br atom, could give 3m and 3q in 44% and 49% yields, respectively. 2,2-Diethoxyacetic acid also reacted with *ortho*-, *meta*-, and *para*-Cl-substituted phenylacetylene bromides to afford desired products 3n–3p in 48–55% yields. Substrates with strongly electron-withdrawing CF_3_ (3r, 42%) and CN (3s, 43% and 3t, 55%) substituents on the benzene ring were satisfactory as well, and the location of the CN group slightly affected the yield: the *ortho*-substituted compound gave a higher yield (55%) than the *para*-substituted compound (43%). Carbonyl substituents were also tolerated: ketone- and ester-bearing phenylacetylene bromides yielded the corresponding products (3u and 3v) in 30–45% yields. We also found that the presence of multiple Cl atoms on the benzene ring did not affect the yield; 3w was obtained in 40% yield. We also explored the reactivity of substrates in which the phenyl ring had been replaced by a heterocycle and found that electron-rich thiophene (3x, 34%) and indole (3z, 40%) rings and an electron-deficient aromatic pyridine ring (3y, 56%) were tolerated.

**Scheme 2:**
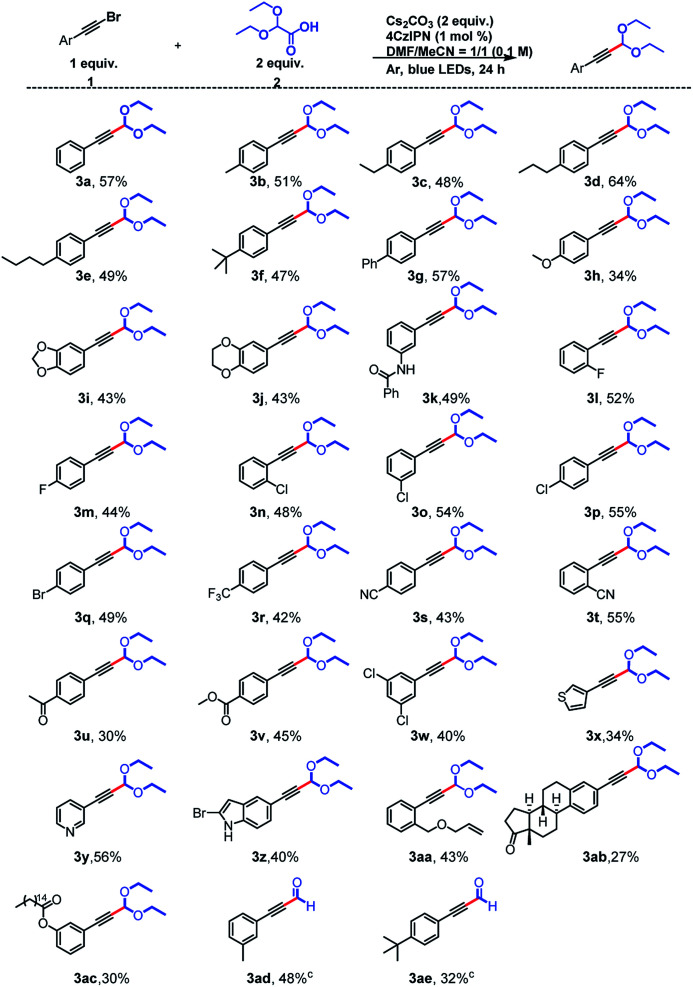
Scope of the reaction with respect to the alkynyl bromide. ^a^Standard conditions: a mixture of 1g (0.3 mmol, 1.0 equiv.), 2 (0.6 mmol, 2.0 equiv.), 4CzIPN (0.003 mmol, 1 mol%), and Cs_2_CO_3_ (0.6 mmol, 2.0 equiv.) in 1 : 1 (v/v) DMF/MeCN (3 mL total) was irradiated with 5 W blue LEDs at room temperature under argon for 24 h. ^b^Isolated yields are provided. ^c^Acetals were not separated, and the subsequent removal of the acetal group was directly carried out, and the yield is the total yield of the two-step reaction.

Alkenyl groups can also act as acceptors for acetal radicals, as reported by Xu^10^ and Wang.^13^ However, when we carried out the reaction between 2 and an olefin-containing substrate, we isolated (3aa, 43%), which indicates that the olefin did not participate in the reaction. In addition, polycyclic product (3ab, 27%) was prepared from a Br-substituted derivative of estrone, which is a steroid hormone and an endogenous estrogen. We were also able to obtain 3ac (30%) from a substrate derived from palmitic acid, which is an important component of blood lipids. The derivatization of these biologically relevant compounds indicates the potential utility of this protocol. Finally, when the products of the acetalation reactions of 1-(bromoethynyl)-3-methylbenzene and 1-(bromoethynyl)-4-(*tert*-butyl)benzene were directly protonated,^14^ the corresponding hydroformylation products (3ad and 3ae) were obtained with yield of 48% and 32%.

To gain insight into the reaction mechanism, we conducted some radical-trapping and radical-inhibition experiments (Scheme 3). When the reaction of alkynyl bromide 1g was carried out in the presence of the radical inhibitor TEMPO (2,2,6,6-tetramethylpiperidin-1-oxyl), no reaction occurred and none of the desired product was observed, a result that supports the involvement of radical intermediates (eq. (1)). In addition, the acetal radical could be trapped by ethene-1,1-diyldibenzene (eq. (2)), as confirmed by high-resolution mass spectrometry. Surprisingly, when 2 is omitted, 1g of debromination occurs in the presence of Cs_2_CO_3_ and a photocatalyst, which consumes a portion of the alkynyl bromide, wherein the terminal alkyne may be derived from less water in the solvent as its proton hydrogen source^15^ and it may be the main cause of low yield. Because terminal alkynes are known to act as radical acceptors,^16^ we wondered whether a terminal alkyne could undergo the acetalation reaction. To explore this possibility, we carried out two experiments with 1-ethynyl-4-phenylbenzene. When this substrate was subjected to the standard conditions, the acetalation reaction did not proceed (eq. (4)). However, when *N*-bromosuccinimide and AgNO_3_ were present in the reaction mixture (eq. (5)), 3g was obtained in 52% yield, which confirms that an alkynyl bromide was the true reactive species.

**Scheme 3:**
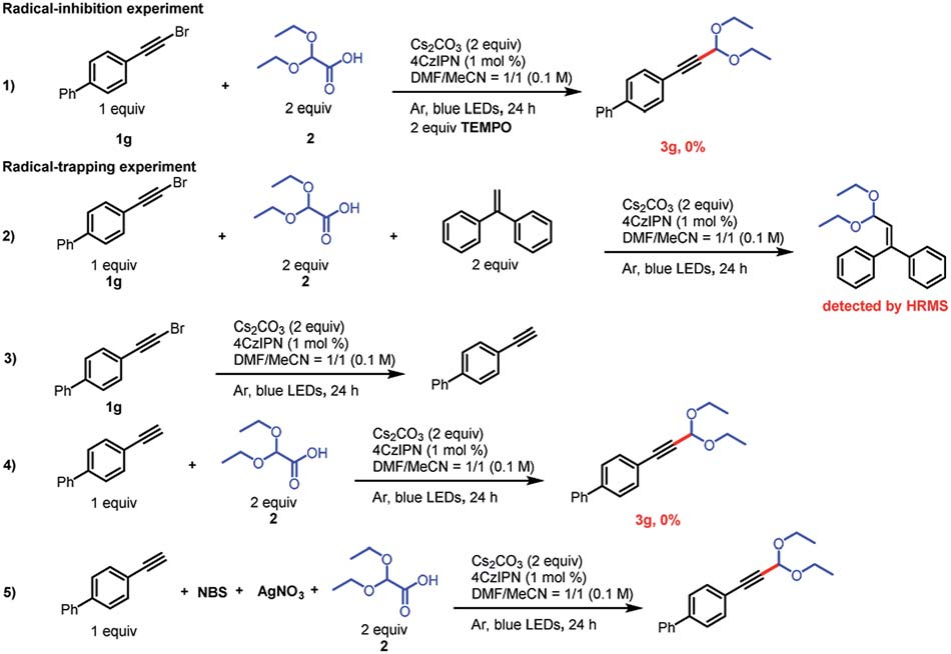
Mechanistic experiments.

On the basis of our mechanistic studies, we propose the mechanism outlined in Scheme 4. First, the metal-free photocatalyst 4CzIPN is photoexcited to 4CzIPN* by irradiation with the 5 W blue LEDs. The cesium salt of 2,2-diethoxyacetic acid (*E*_ox_ = +0.95 V *vs.* SCE)^17^ readily undergoes a single-electron transfer reaction with 4CzIPN* to form carboxyl radical intermediate I. Intermediate I then loses a molecule of CO_2_ to form acetal radical II, which adds to arylethynyl bromide 1g to give stable bromoalkenyl radical III.^18^ Collapse of this radical gives product 3g and a bromine radical.^19^ The bromine radical (*E*_1/2_^red^ [Br/Br^−^] = +0.80 V *vs.* SCE in DME)^20^ is reduced to a bromine anion by the reduced-state photocatalyst (*E* [PC/PC˙^−^] = −1.21 V *vs.* SCE),^12^ which completes the catalytic cycle. However, we cannot exclude another mechanism involving alkynyl radicals, whose generation has been reported under similar conditions.^21^

**Scheme 4:**
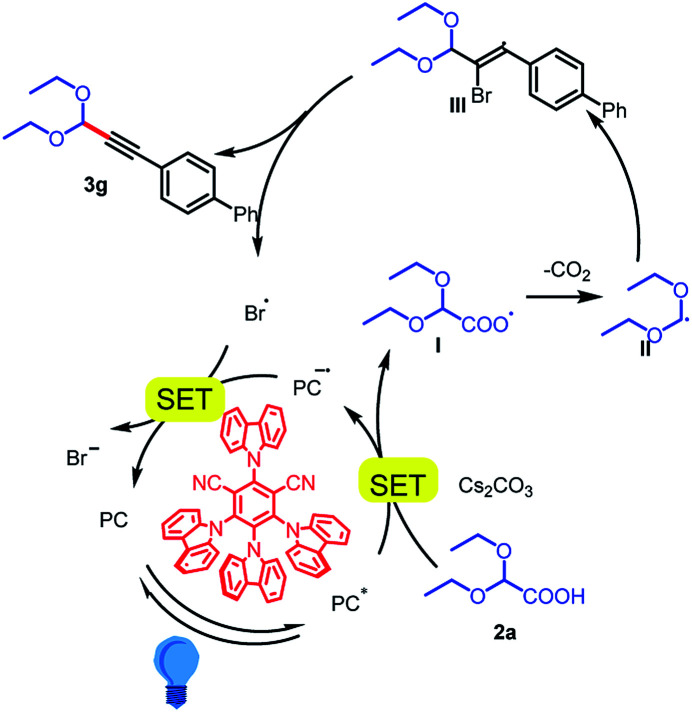
Possible reaction mechanism.

## Conclusions

In summary, we have developed a protocol for visible-light-promoted acetalation of alkynyl bromides with readily available 2,2-diethoxyacetic acid as the acetal source. The alkynyl bromide substrates can be synthesized easily, and the acetalation reaction proceeds without the need for an additional oxidant and is therefore compatible with various functional groups. In addition to arylethynyl bromides, alkynyl bromides bearing heteroaryl rings (thiophene, pyridine, and indole) were also effective substrates.

## Conflicts of interest

There are no conflicts to declare.

## Supplementary Material

RA-009-C9RA06596B-s001
