# Supplementary material for: Blue light photoredox-catalysed acetalation of alkynyl bromides
Source: RSC Adv. 2019 Nov 6;9(62):36213–6. doi: 10.1039/c9ra06596b (PMC9074944; doi:10.1039/c9ra06596b)
Supplement: RA-009-C9RA06596B-s001 [file RA-009-C9RA06596B-s001.pdf]

Electronic Supplementary Material (ESI) for RSC Advances.
This journal is © The Royal Society of Chemistry 2019

Blue Light Photoredox-Catalysed Acetalation of Alkynyl Bromides

Xue-Li Lyu, † Shi-Sheng Huang, † Hong-Jian Song,*† Yu-Xiu Liu*† and Qing-Min Wang*†‡

†State Key Laboratory of Elemento-Organic Chemistry, Research Institute of Elemento-Organic Chemistry, College of Chemistry, Nankai University, Tianjin 300071, People's Republic of China.

‡Collaborative Innovation Center of Chemical Science and Engineering (Tianjin), Tianjin 300071, People's Republic of China.

Table of contents

1. General Information 2
2. Optimization of reaction conditions 2
  2.1 Photocatalyst screening for the reaction 2
  2.2 Solvent screening for the reaction 2
  2.3 Base screening for the reaction 3
  2.4 Leaving group screening for the reaction 4
  2.5 Loading of 2 screening for the reaction 5
3. General procedure of compounds 1 5
  3.1 General Procedure for the synthesis of 1. 5
  3.2 Procedure for the synthesis of 3-(bromoethynyl) thiophene 7
  3.3 Procedure for the synthesis of 3-(bromoethynyl) pyridine 8
4. General procedure of compounds 2 8
5. General procedure of photocatalyst 4CzIPN 9
6. General procedure of compounds 3 and removal of the acetal group 10
  General procedure of compounds 3 10
  Removal of the acetal group 10
7. Characterization of products 11
8 Another proposed mechanism. 21
9. References 22
10. Copies of ^{1}H NMR ^{13}C NMR and ^{19}F NMR spectra for compounds 22

1. General Information

All reagents were used as received. 1 H and 13 C Nuclear Magnetic Resonance (NMR) spectra were recorded on a Bruker Avance 400 Ultrashield NMR spectrometers. Chemical shifts (δ) were given in parts per million (ppm) and were measured downfield from internal tetramethylsilane. High-resolution mass spectrometry (HRMS) data were obtained on an FTICR-MS instrument (Ionspec 7.0 T). The melting points were determined on an X-4 binocular microscope melting point apparatus (Beijing Tech Instruments Co., Beijing, China) and are uncorrected. Conversion was monitored by thin layer chromatography (TLC). Flash column chromatography was performed over silica gel (100-200 mesh).

2. Optimization of reaction conditions

2.1 Photocatalyst screening for the reaction

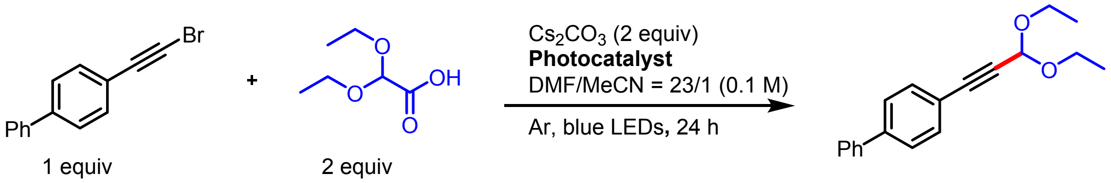

Table S1. Photocatalyst screening for the reaction

Entry	Photocatalyst	Loading of Photocatalyst	Yield/%
**1**	$Ru(bpy)_3Cl_2{\cdot}6H_2O$	2 mol %	0
**2**	$Ru(bpy)_3(PF_6)_2$	2 mol %	0
**3**	Fluorescein	10 mol %	0
**4**	Eosin Y	10 mol %	0
**5**	Mes-Acr$^+$ClO4$^-$	10 mol %	0
6	***4CzIPN***	***2 mol %***	***36***
**7**	Ir[(dF(CF3)ppy)$_2$(dtbbpy)]PF$_6$	2 mol %	5

2.2 Solvent screening for the reaction

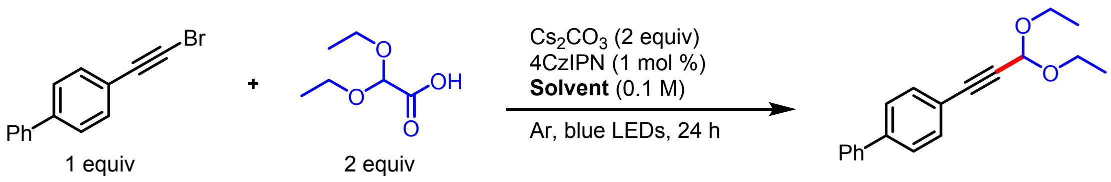

Table S2. Slovent screening for the reaction

Entry	Solvent	Yield/%
**1**	DCM	0
**2**	MeCN	25
**3**	Toluene	0
**4**	PhCl	0
**5**	DMA	36
**6**	DMF	43
**7**	DMSO	41
**8**	1,4 – dioxane	0
**9**	$CHCl_3$	0
**10**	Acetone	12
**11**	$PhCF_3$	0
12	***DMF/MeCN = 1/1***	***57***

2.3 Base screening for the reaction

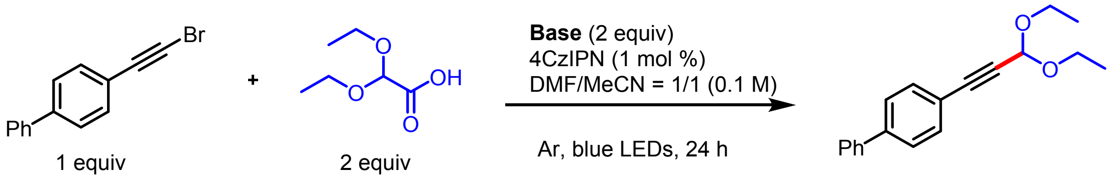

Table S3. Base screening for the reaction

Entry	Base	Yield
**1**	K_2HPO_4	40
**2**	CsF	0
**3**	K_2CO_3	52
**4**	Li_2CO_3	0
**5**	Na_2CO_3	47
**6**	KOH	0
**7**	CsOH	0
**8**	KOAc	40
**9**	2,4 – lutidine	0
10	***Cs_2CO_3***	***57***

2.4 Leaving group screening for the reaction

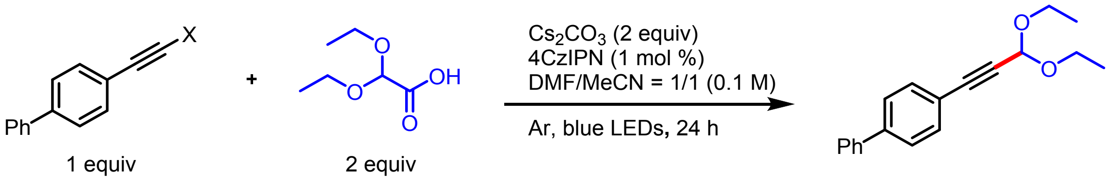

Table S4. Leaving group screening for the reaction

Entry	X	Yield/%
**1**	Cl	37
2	***Br***	***57***
**3**	I	Trace

**4**	H	0
**5**	BI	0
**6**	TMS	0
**7**	Ts	0
**8**	COOH	0

2.5 Loading of 2 screening for the reaction

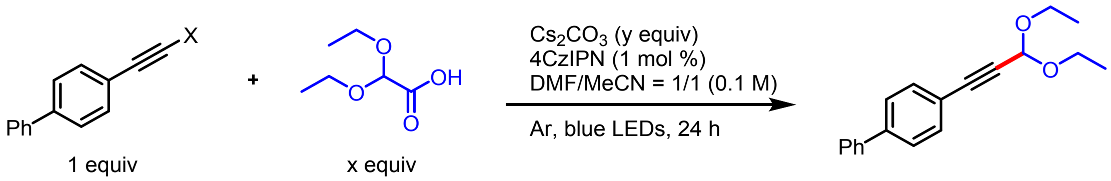

Table S5. Loading of 2 screening for the reaction

Entry	x	y	Yield/%
1	*2*	*2*	*57*
**2**	5	5	30
**3**	5	10	49
**4**	10	10	44

3. General procedure of compounds 1

3.1 General Procedure for the synthesis of 1.

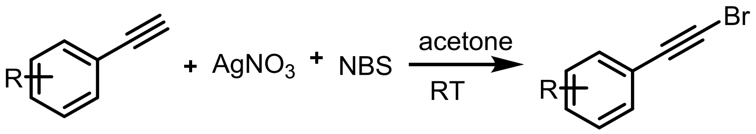

To the round bottom flask, terminal alkyne (1.0 equiv) and $AgNO_3$ (0.1 equiv) were sequentially added, then acetone was added, and then NBS (1.2 equiv) was added in portions under stirring, and the mixture was stirred at room temperature until the

reaction was completely monitored by TLC. After the reaction was completed, the solvent was removed under vacuum. The desired products were obtained in the corresponding yields after purification by flash chromatography on silica gel eluting with PE and ethyl acetate.

1-(bromoethynyl)-2-chlorobenzene

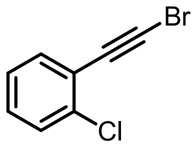

^{1}H NMR (400 MHz, $CDCl_3$) δ 7.48 (d, J = 7.6 Hz, 1H), 7.39 (d, J = 8.0 Hz, 1H), 7.27 (t, J = 6.8 Hz, 1H), 7.21 (t, J = 7.6 Hz, 1H). **^{13}C NMR** (100 MHz, $CDCl_3$) δ 136.5, 134.0, 129.8, 129.4, 126.6, 122.8, 100.1, 55.4.

***N*-(3-(bromoethynyl) phenyl) benzamide**

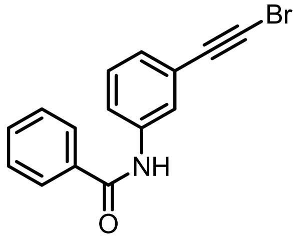

^{1}H NMR (400 MHz, $CDCl_3$) δ 7.89 (d, J = 7.2 Hz, 2H), 7.79 (s, 1H), 7.77 (s, 1H), 7.69 (d, J = 8.0 Hz, 1H), 7.60 (t, J = 7.2 Hz, 1H), 7.53 (t, J = 7.6 Hz, 2H), 7.35 (t, J = 8.0 Hz, 1H), 7.26 (d, J = 7.6 Hz, 1H). **^{13}C NMR** (100 MHz, $CDCl_3$) δ 165.8, 138.1, 134.8, 132.2, 129.3, 129.0, 128.2, 127.2, 123.7, 123.6, 120.7, 79.7, 50.6.

6-(bromoethynyl)-2,3-dihydrobenzo[*b*] [1,4] dioxine

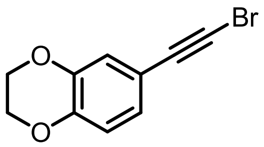

^{1}H NMR (400 MHz, $CDCl_3$) δ 6.89 – 6.81 (m, 2H), 6.68 (d, J = 8.4 Hz, 1H), 4.13 (d, J = 2.4 Hz, 4H). **^{13}C NMR** (100 MHz, $CDCl_3$) δ 144.5, 143.2, 125.6, 120.9, 117.4, 115.4, 79.8, 64.5, 64.2, 48.0.

1-((allyloxy)methyl)-2-(bromoethynyl) benzene

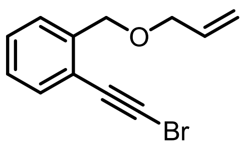

^{1}H NMR (400 MHz, $CDCl_3$) δ 7.37 (dd, J = 14.8, 7.6 Hz, 2H), 7.26 (t, J = 7.6 Hz, 1H), 7.14 (t, J = 7.6 Hz, 1H), 5.90 (ddd, J = 22.4, 10.8, 5.6 Hz, 1H), 5.26 (d, J = 17.2 Hz, 1H), 5.14 (d, J = 10.4 Hz, 1H), 4.58 (s, 2H), 4.00 (d, J = 5.2 Hz, 2H). **^{13}C NMR** (100 MHz, $CDCl_3$) δ 141.0, 134.74, 132.7, 129.0, 127.7, 127.4, 121.3, 117.3, 78.0, 71.7, 70.1, 53.9.

(8*R*,9*S*,13*S*,14*S*)-3-(bromoethynyl)-13-methyl-6,7,8,9,11,12,13,14,15,16-decahydro-17*H*-cyclopenta[*a*]phenanthren-17-one

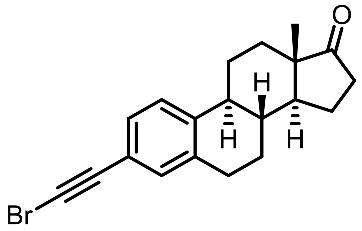

^{1}H NMR (400 MHz, $CDCl_3$) δ 7.23 (t, J = 12.8 Hz, 3H), 2.88 (s, 1H), 2.66 – 1.08 (m, 14H), 0.91 (s, 3H). **^{13}C NMR** (100 MHz, $CDCl_3$) δ 220.8, 140.9, 136.8, 132.6, 129.4, 125.5, 120.2, 80.2, 50.6, 48.9, 48.1, 44.6, 38.1, 36.0, 31.7, 29.2, 26.4, 25.7, 21.7, 14.0.

3-(bromoethynyl)phenyl palmitate

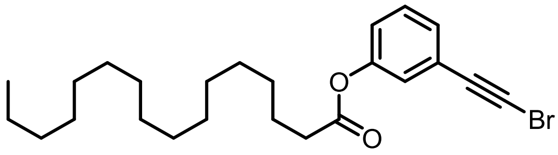

^{1}H NMR (400 MHz, $CDCl_3$) δ 7.30 (s, 2H), 7.17 (s, 1H), 7.07 (s, 1H), 2.54 (t, J = 7.2 Hz, 2H), 1.78 – 1.69 (m, 2H), 1.26 (s, 24H), 0.88 (t, J = 6.4 Hz, 3H). **^{13}C NMR** (100 MHz, $CDCl_3$) δ 172.2, 150.6, 129.5, 129.5, 125.3, 124.1, 122.5, 79.2, 51.0, 34.5, 32.1, 29.8, 29.8, 29.7, 29.6, 29.5, 29.4, 29.2, 25.0, 22.9, 14.3.

3.2 Procedure for the synthesis of 3-(bromoethynyl) thiophene

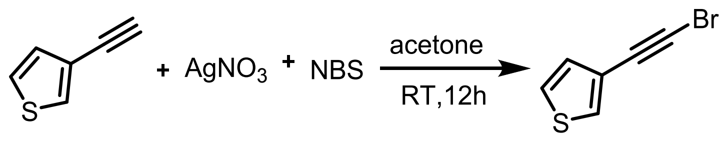

To the round bottom flask, 3-thienyl acethylene (500 mg, 4.62 mmol, 1.0 equiv) and $AgNO_3$ (78.1 mg, 0.46 mmol, 0.1 equiv) were sequentially added, then acetone (20 mL) was added, and then NBS (987.3 mg, 5.55 mmol, 1.2 equiv) was added in portions under stirring, and the mixture was stirred at room temperature for 12 hours. After the reaction was completed, the solvent was removed under vacuum. The desired products were obtained in the corresponding yields after purification by flash chromatography on silica gel eluting with PE. (brown oil, 52%).

3-(bromoethynyl)thiophene

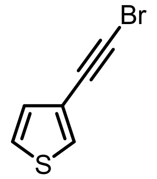

^{1}H NMR (400 MHz, $CDCl_3$) δ 7.48 (dd, J = 3.2, 1.2 Hz, 1H), 7.26 – 7.23 (m, 1H), 7.11 (dd, J = 5.2, 1.2 Hz, 1H). **^{13}C NMR** (100 MHz, $CDCl_3$) δ 129.8, 129.8, 125.4, 121.7, 75.4, 49.5.

3.3 Procedure for the synthesis of 3-(bromoethynyl) pyridine

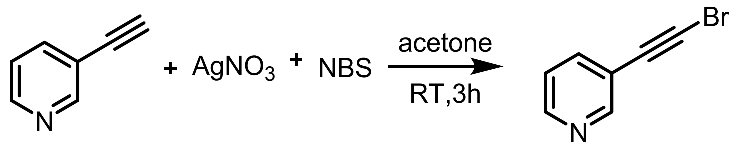

To the round bottom flask, 3-Ethynylpyridine (250 mg, 2.42 mmol, 1.0 equiv) and $AgNO_3$(40.8 mg, 0.24 mmol, 0.1 equiv) were sequentially added, then acetone (10 mL) was added, and then NBS (517.8 mg, 2.91 mmol, 1.2 equiv) was added in portions under stirring, and the mixture heated to reflux for three hours. After completion of the reaction, the solvent was removed under vacuum. The desired products were obtained in the corresponding yields after purification by flash chromatography on silica gel eluting with PE/ethyl acetate = 5/1. (white solid, 68%).

4. General procedure of compounds 2

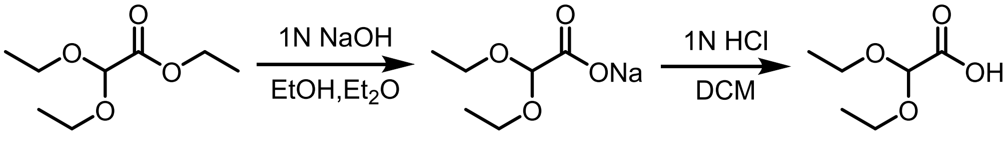

Add 1N NaOH (26 mL, 1.2 equiv) aqueous solution to 2,2-diethoxyacetate (3,86 mL, 21.6 mmol, 1.0 equiv), stir at room temperature for 3 h, then the solution was removed under vacuum, extract the aqueous phase with diethyl ether (20 mL × 3) to wash off unreacted 2,2-diethoxyacetate. Then add 1N HCl under stirring at -10 °C until the reaction solution is acidic. The reaction solution is slowly warmed to room temperature and stirred for another 2 h. Finally, it is extracted with DCM (20 mL × 3) washed with saturated brine, dried over anhydrous Na_2SO_4. The solvent was removed under vacuum to give the product (light yellow oil, 82%).

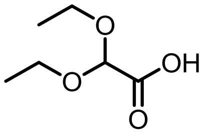

^{1}H NMR (400 MHz, $CDCl_3$) δ 10.21 (s, 1H), 4.95 (s, 1H), 3.75 – 3.61 (m, 4H), 1.24 (t, J = 7.2 Hz, 6H).

5. General procedure of photocatalyst 4CzIPN

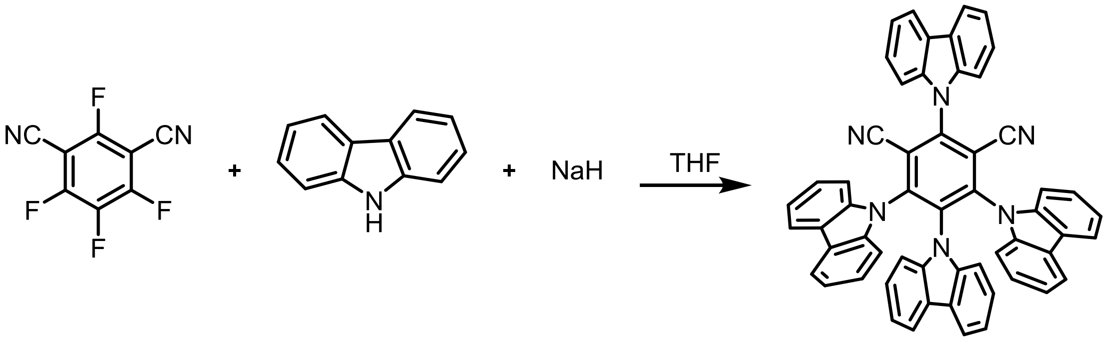

Add 9H – carbazole (1.67 g, 10 mmol, 5 equiv) and a magneton to a 100 mL four-necked flask, then replace the gas three times under argon, add THF (20 mL) to the system through a syringe, place the device in an ice bath at 0 ° C, then add NaH (0.6 g, 15 mmol, 7.5 equiv) under stirring slowly, continue to stir for another 0.5 h, after then add 2,4,5,6 – tetrafluoroisophthalonitrile (0.4 g, 2.0 mmol, 1.0 equiv) The four-necked flask was slowly warmed to room temperature and stirred for 12 h. After completion of the reaction, the reaction was quenched by the addition of 2 mL H_2O. The solvent was removed under vacuum, the crude product was washed with water and ethanol, and then recrystallized from dichloromethane and n-hexane. The desired products were obtained in the corresponding yields after purification by flash chromatography on silica gel eluting with DCM/n-hexane = 4/1. (1.4 g, bright yellow crystalline solid, 87%).

1,2,3,5-tetrakis(carbazol-9-yl)-4,6-dicyanobenzene

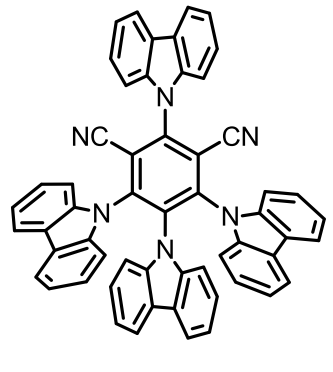

^{1}H NMR (400 MHz, $CDCl_3$) δ 8.22 (d, J = 7.6 Hz, 2H), 7.74 – 7.67 (m, 8H), 7.52 – 7.47 (m, 2H), 7.33 (d, J = 7.6 Hz, 2H), 7.22 (dd, J = 6.8, 2.0 Hz, 4H), 7.08 (m, 8H), 6.82 (t, J = 8.4 Hz, 4H), 6.63 (t, J = 7.6 Hz, 2H).

6. General procedure of compounds 3 and removal of the acetal group

General procedure of compounds 3

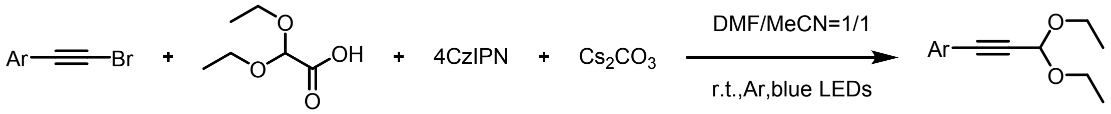

To an oven-dried 8 mL reaction flask equipped with a stir bar, was added arylethynyl bromide (0.3 mmol, 1.0 equiv), 2,2 -diethoxyacetic acid (88.8 mg, 0.6 mmol, 2.0 equiv), 4CzIPN (2.3 mg, 0.003 mmol, 0.01 equiv), Cs_2CO_3 (195.5 mg, 0.6 mmol, 2.0 equiv). Then replace the gas three times under argon. After then, DMF (1.5 mL) and MeCN (1.5 mL) were injected into the tube by syringe. The solution was then stirred at room temperature under the irradiation of 5 W blue LED for 24 h. After completion of the reaction, Then the solution was extracted with DCM (10 mL × 3). The combined organic layer was washed with water (30 mL × 3), and then was washed with brine and dried over anhydrous Na_2SO_4 and evaporated in vacuum. The desired products were obtained in the corresponding yields after purification by flash chromatography on silica gel eluting with PE and ethyl acetate.

Removal of the acetal group

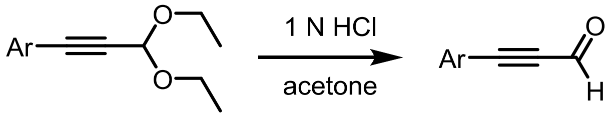

Adding 0.5 mL 1 N HCl to the above product, stirred for 2 h and extracted with DCM (10 mL × 3). The combined organic layer was washed with brine and then dried over anhydrous Na_2SO_4 and evaporated in vacuum. The desired products were obtained in the corresponding yields after purification by flash chromatography on silica gel eluting with hexanes and ethyl acetate.

7. Characterization of products

(3, 3-diethoxyprop-1-yn-1-yl) benzene (3a)

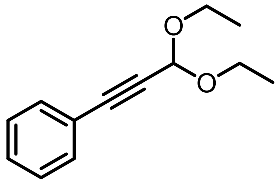

Colourless oil, yield 57%, **^{1}H NMR** (400 MHz, $CDCl_3$) δ 7.48 (d, J = 7.6 Hz, 2H), 7.32 (t, J = 6.4 Hz, 3H), 5.50 (s, 1H), 3.87 – 3.79 (m, 2H), 3.71 – 3.63 (m, 2H), 1.28 (t, J = 7.2 Hz, 6H). **^{13}C NMR** (100 MHz, $CDCl_3$) δ 132.1, 128.9, 128.4, 122.1, 92.0, 85.4, 84.6, 61.1, 15.3. NMR data were in close agreement with the literature[1].

1-(3, 3-diethoxyprop-1-yn-1-yl)-4-methylbenzene (3b)

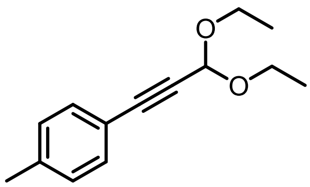

Colourless oil, yield 51%, **^{1}H NMR** (400 MHz, $CDCl_3$) δ 7.37 (d, J = 8.0 Hz, 2H), 7.12 (d, J = 8.0 Hz, 2H), 5.49 (s, 1H), 3.82 (m, 2H), 3.66 (m, 2H), 2.35 (s, 3H), 1.28 (t, J = 7.1 Hz, 6H). **^{13}C NMR** (100 MHz, $CDCl_3$) δ 139.1, 132.0, 129.2, 119.0, 92.0, 85.6, 83.9, 61.1, 21.7, 15.3. NMR data were in close agreement with the literature[2].

1-(3,3-diethoxyprop-1-yn-1-yl)-4-ethylbenzene(3c)

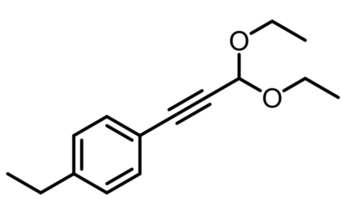

Colourless oil, yield 48%, **^{1}H NMR** (400 MHz, $CDCl_3$) δ 7.39 (d, J = 8.0 Hz, 2H), 7.14 (d, J = 8.0 Hz, 2H), 5.49 (s, 1H), 3.87 – 3.78 (m, 2H), 3.71 – 3.61 (m, 2H), 2.63 (q, J =

7.6 Hz, 2H), 1.27 (t, J = 7.2 Hz, 6H), 1.22 (t, J = 7.6 Hz, 3H). **^{13}C NMR** (100 MHz, $CDCl_3$) δ 145.3, 132.0, 127.9, 119.1, 92.0, 85.5, 83.8, 61.0, 28.9, 15.4, 15.2. The compound is new compound.

1-(3, 3-diethoxyprop-1-yn-1-yl)-4-propylbenzene(3d)

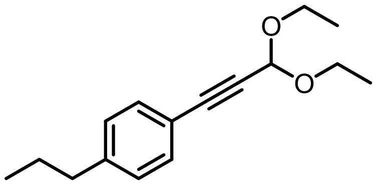

Colourless oil, yield 64%, **^{1}H NMR** (400 MHz, $CDCl_3$) δ 7.39 (d, J = 6.8 Hz, 2H), 7.12 (d, J = 6.8 Hz, 2H), 5.49 (s, 1H), 3.88 – 3.76 (m, 2H), 3.73 – 3.61 (m, 2H), 2.57 (t, J = 6.8 Hz, 2H), 1.62 (dd, J = 14.0, 6.8 Hz, 2H), 1.27 (t, J = 6.0 Hz, 6H), 0.92 (t, J = 6.4 Hz, 3H). **^{13}C NMR** (100 MHz, $CDCl_3$) δ 143.8, 132.0, 128.5, 119.1, 92.0, 85.6, 83.8, 61.0, 38.1, 24.4, 15.3, 13.9. The compound is new compound.

1-butyl-4-(3,3-diethoxyprop-1-yn-1-yl) benzene(3e)

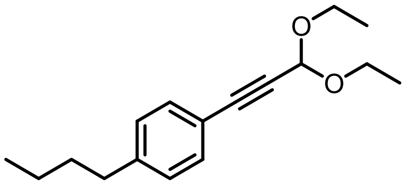

Yellow oil, yield 49%, **^{1}H NMR** (400 MHz, $CDCl_3$) δ 7.38 (d, J = 8.0 Hz, 2H), 7.12 (d, J = 8.0 Hz, 2H), 5.49 (s, 1H), 3.87 – 3.76 (m, 2H), 3.71 – 3.61 (m, 2H), 2.62 – 2.56 (m, 2H), 1.62 – 1.53 (m, 2H), 1.35 (dd, J = 14.0, 6.8 Hz, 2H), 1.27 (t, J = 7.2 Hz, 6H), 0.91 (t, J = 7.2 Hz, 3H). **^{13}C NMR** (100 MHz, $CDCl_3$) δ 144.1, 132.0, 128.5, 119.1, 92.0, 85.6, 83.8, 61.0, 35.7, 33.5, 22.4, 15.3, 14.0. The compound is new compound.

1-(tert-butyl)-4-(3,3-diethoxyprop-1-yn-1-yl) benzene (3f)

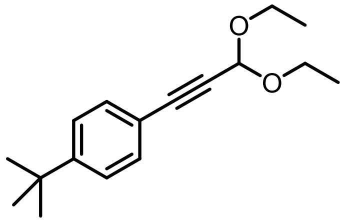

Yellow oil, yield 47%, **^{1}H NMR** (400 MHz, $CDCl_3$) δ 7.41 (d, J = 8.4 Hz, 2H), 7.33 (d, J = 8.4 Hz, 2H), 5.49 (s, 1H), 3.86 – 3.79 (m, 2H), 3.70 – 3.62 (m, 2H), 1.30 (s, 9H), 1.27 (t, J = 7.2 Hz, 6H). **^{13}C NMR** (100 MHz, $CDCl_3$) δ 152.2, 131.8, 125.4, 118.9,

92.0, 85.5, 83.8, 61.0, 34.9, 31.3, 15.3. NMR data were in close agreement with the literature[2].

4-(3, 3-diethoxyprop-1-yn-1-yl)-1,1'-biphenyl (3g)

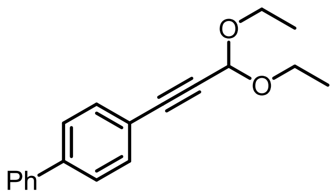

Colourless oil, yield 57%, **^{1}H NMR** (400 MHz, $CDCl_3$) δ 7.66 – 7.56 (m, 6H), 7.48 (t, *J* = 7.6 Hz, 2H), 7.40 (t, *J* = 7.2 Hz, 1H), 5.57 (s, 1H), 3.99 – 3.82 (m, 2H), 3.82 – 3.65 (m, 2H), 1.34 (t, J = 7.1 Hz, 6H). **^{13}C NMR** (100 MHz, $CDCl_3$) δ 140.7, 139.3, 131.5, 128.0, 126.9, 126.2, 126.1, 119.9, 91.0, 84.2, 84.1, 60.1, 14.3. The compound is new compound.

1-(3,3-diethoxyprop-1-yn-1-yl)-4-methoxybenzene(3h)

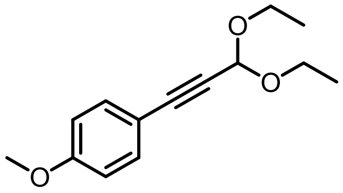

Colourless oil, yield 34%, **^{1}H NMR** (400 MHz, $CDCl_3$) δ 7.41 (d, *J* = 8.4 Hz, 2H), 6.83 (d, *J* = 8.5 Hz, 2H), 5.48 (s, 1H), 3.87 – 3.76 (m, 5H), 3.70 – 3.61 (m, 2H), 1.27 (t, *J* = 7.2 Hz, 6H). **^{13}C NMR** (100 MHz, $CDCl_3$) δ 160.1, 133.5, 114.0, 114.0, 92.0, 85.4, 83.2, 61.0, 55.4, 15.3. NMR data were in close agreement with the literature[2].

5-(3, 3-diethoxyprop-1-yn-1-yl) benzo[*d*] [1, 3] dioxole (3i)

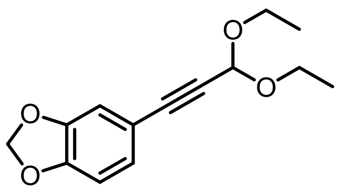

Yellow oil, yield 43%, **^{1}H NMR** (400 MHz, $CDCl_3$) δ 7.00 (d, *J* = 9.6 Hz, 1H), 6.91 (s, 1H), 6.74 (d, *J* = 8.0 Hz, 1H), 5.97 (s, 2H), 5.46 (s, 1H), 3.85 – 3.76 (m, 2H), 3.64 (m, 2H), 1.27 (t, *J* = 7.2 Hz, 6H). **^{13}C NMR** (100 MHz, $CDCl_3$) δ 148.4, 147.5, 126.9, 115.2, 112.0, 108.5, 101.5, 91.9, 85.3, 82.9, 61.0, 15.3. The compound is new compound.

6-(3, 3-diethoxyprop-1-yn-1-yl)-2,3-dihydrobenzo[*b*] [1,4] dioxine (3j)

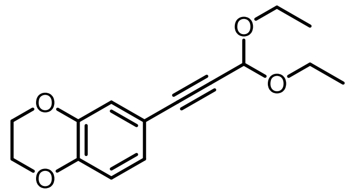

Colourless oil, yield 43%,^{1}**H NMR** (400 MHz, $CDCl_3$) δ 6.98 (d, J = 1.6 Hz, 1H), 6.96 (d, J = 8.4 Hz, 1H), 6.77 (d, J = 8.4 Hz, 1H), 5.46 (s, 1H), 4.23 (q, J = 4.8 Hz, 4H), 3.85 – 3.75 (m, 2H), 3.68 – 3.59 (m, 2H), 1.26 (t, J = 7.2 Hz, 6H). ^{13}**C NMR** (100 MHz, $CDCl_3$) δ 144.6, 143.3, 125.7, 120.9, 117.4, 114.7, 91.9, 85.1, 82.9, 64.5, 64.3, 61.0, 15.2. The compound is new compound.

N-(3-(3, 3-diethoxyprop-1-yn-1-yl) phenyl) benzamide (3k)

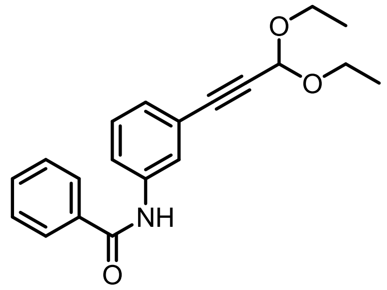

Colourless solid, yield 49%, M.p 125°C,^{1}**H NMR** (400 MHz, $CDCl_3$) δ 8.01 (s, 1H), 7.83 (d, J = 7.2 Hz, 2H), 7.74 (s, 1H), 7.66 (d, J = 8.0 Hz, 1H), 7.53 (t, J = 7.2 Hz, 1H), 7.45 (t, J = 7.6 Hz, 2H), 7.28 (t, J = 8.0 Hz, 1H), 7.24 (d, J = 7.6 Hz, 1H), 5.48 (s, 1H), 3.81 (m, 2H), 3.65 (m, 2H), 1.27 (t, J = 7.2 Hz, 6H). ^{13}**C NMR** (100 MHz, $CDCl_3$) δ 166.0, 138.1, 134.8, 132.1, 129.2, 128.9, 128.2, 127.2, 123.5, 122.8, 120.9, 91.9, 84.8, 84.8, 61.1, 15.2. The compound is new compound.

1-(3, 3-diethoxyprop-1-yn-1-yl)-2-fluorobenzene(3l)

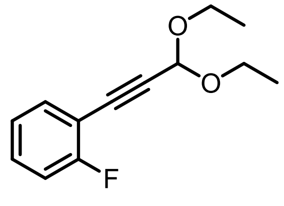

yellowish oil, yield 52%, ^{1}**H NMR** (400 MHz, $CDCl_3$) δ 7.49 – 7.43 (m, 1H), 7.31 (tdd, J = 7.6, 5.4, 1.6 Hz, 1H), 7.07 (dd, J = 16.8, 8.8 Hz, 2H), 5.51 (s, 1H), 3.83 (m, 2H), 3.67 (m, 2H), 1.27 (t, J = 7.2 Hz, 6H). ^{13}**C NMR** (100 MHz, $CDCl_3$) δ 163.1(d, J = 251.0 Hz), 133.8, 130.71 (d, J = 7.9 Hz), 124.00 (d, J = 3.9 Hz), 115.6 (d, J = 21.0 Hz), 110.7 (d, J = 15.8 Hz), 91.8, 89.7, 78.8, 61.2, 15.2. ^{19}**F NMR** (376 MHz, $CDCl_3$) δ -

113.95 (dd, J = 14.7, 6.5 Hz, 1F). NMR data were in close agreement with the literature[2].

1-(3, 3-diethoxyprop-1-yn-1-yl)-4-fluorobenzene (3m)

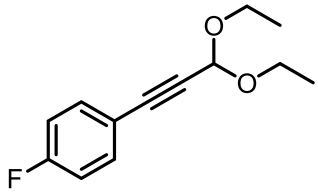

Colourless oil, yield 44%, ^{1}**H NMR** (400 MHz, $CDCl_3$) δ 7.48 – 7.41 (m, 2H), 7.00 (t, J = 8.8 Hz, 2H), 5.47 (s, 1H), 3.87 – 3.76 (m, 2H), 3.70 – 3.60 (m, 2H), 1.27 (t, J = 7.2 Hz, 6H). ^{13}**C NMR** (100 MHz, $CDCl_3$) δ 164.2 (d, J = 252.5 Hz), 134.0 (d, J = 8.5 Hz), 118.1, 115.7 (d, J = 22 Hz), 91.9, 84.3, 77.5, 61.1, 15.2. ^{19}**F NMR** (376 MHz, $CDCl_3$) δ -114.48 – -114.76 (m). 1F NMR data were in close agreement with the literature[2].

1-chloro-2-(3,3-diethoxyprop-1-yn-1-yl) benzene(3n)

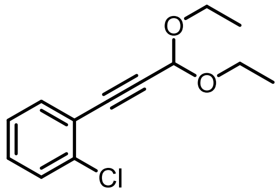

Colourless oil, yield 48%, ^{1}**H NMR** (400 MHz, $CDCl_3$) δ 7.51 (d, J = 7.6 Hz, 1H), 7.39 (d, J = 8.0 Hz, 1H), 7.27 (t, J = 7.2 Hz, 1H), 7.20 (t, J = 7.6 Hz, 1H), 5.54 (s, 1H), 3.87 (m, 2H), 3.69 (m, 2H), 1.28 (t, J = 7.2 Hz, 6H). ^{13}**C NMR** (100 MHz, $CDCl_3$) δ 136.3, 133.8, 130.0, 129.4, 126.5, 122.1, 91.9, 89.7, 82.0, 61.2, 15.3. The compound is new compound.

1-chloro-3-(3, 3-diethoxyprop-1-yn-1-yl) benzene (3o)

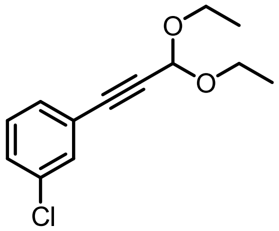

yellowish oil, yield 54%, ^{1}**H NMR** (400 MHz, $CDCl_3$) δ 7.39 (s, 1H), 7.26 (dd, J = 16.0, 7.6Hz, 2H), 7.17 (dd, J = 8.8, 6.8 Hz, 1H), 5.41 (s, 1H), 3.80 – 3.69 (m, 2H), 3.64 – 3.54 (m, 2H), 1.20 (t, J = 6.0 Hz, 6H). ^{13}**C NMR** (100 MHz, $CDCl_3$) δ 134.2, 131.9,

130.2, 129.6, 129.2, 123.7, 91.8, 85.7, 83.8, 61.2, 15.2. The compound is new compound.

1-chloro-4-(3, 3-diethoxyprop-1-yn-1-yl) benzene (3p)

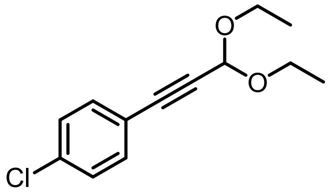

Colourless oil, yield 55%, **^{1}H NMR** (400 MHz, $CDCl_3$) δ 7.40 (d, J = 8.4 Hz, 2H), 7.28 (d, J = 8.4 Hz, 2H), 5.47 (s, 1H), 3.81 (m, 2H), 3.65 (m, 2H), 1.27 (t, J = 7.2 Hz, 6H). **^{13}C NMR** (100 MHz, $CDCl_3$) δ 135.1, 133.3, 128.8, 120.5, 91.8, 85.5, 84.2, 61.1, 15.2. The compound is new compound.

1-bromo-4-(3, 3-diethoxyprop-1-yn-1-yl) benzene (3q)

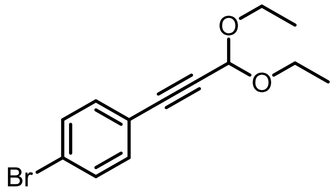

Colourless oil, yield 49%, ^{1}H NMR (400 MHz, $CDCl_3$) δ 7.45 (d, J = 7.2 Hz, 2H), 7.33 (d, J = 7.2 Hz, 2H), 5.47 (s, 1H), 3.86 – 3.76 (m, 2H), 3.70 – 3.61 (m, 2H), 1.27 (t, J = 7.2 Hz, 6H). **^{13}C NMR** (100 MHz, $CDCl_3$) δ 133.4, 131.6, 123.2, 120.8, 91.7, 85.6, 84.1, 61.0, 15.2. The compound is new compound.

1-(3, 3-diethoxyprop-1-yn-1-yl)-4-(trifluoromethyl) benzene (3r)

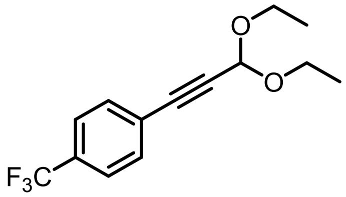

Colourless oil, yield 42%, **^{1}H NMR** (400 MHz, $CDCl_3$) δ 7.57 (s, 4H), 5.50 (s, 1H), 3.86 – 3.78 (m, 2H), 3.70 – 3.63 (m, 2H) 1.28 (t, J = 7.2 Hz, 6H). **^{13}C NMR** (100 MHz, $CDCl_3$) δ 131.3 (q, J = 33 Hz), 125.8, 125.4 (q, J = 3.7 Hz), 122.6, 91.8, 87.0, 83.8, 61.3, 15.3. ^{19}F NMR (376 MHz, $CDCl_3$) δ -67.46 (s). 3F NMR data were in close agreement with the literature[2].

4-(3, 3-diethoxyprop-1-yn-1-yl) benzonitrile (3s)

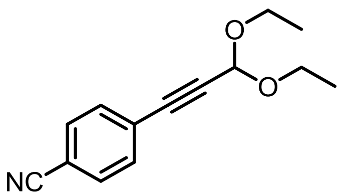

Colourless oil, yield 43%, ^{1}H NMR (400 MHz, $CDCl_3$) δ 7.59 (dd, J = 22.4, 8.4 Hz, 4H), 5.50 (s, 1H), 3.81 (m, 2H), 3.67 (m, 2H), 1.28 (t, J = 7.2Hz, 6H). **^{13}C NMR** (100 MHz, $CDCl_3$) δ 132.5, 132.0, 126.8, 118.3, 112.3, 91.6, 88.7, 83.3, 61.2, 15.1. NMR data were in close agreement with the literature[2].

2-(3, 3-diethoxyprop-1-yn-1-yl) benzonitrile (3t)

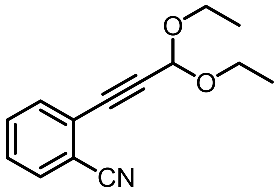

Colourless oil, yield 55%, ^{1}H NMR (400 MHz, $CDCl_3$) δ 7.64 (d, J = 7.6 Hz, 1H), 7.57 (t, J = 6.0 Hz, 1H), 7.53 (d, J = 7.6 Hz, 1H), 7.43 (t, J = 7.6 Hz, 1H), 5.53 (s, 1H), 3.88 – 3.82 (m, 2H), 3.73 – 3.64 (m, 2H), 1.27 (t, J = 7.2 Hz, 6H). **^{13}C NMR** (100 MHz, $CDCl_3$) δ 132.9, 132.8, 132.5, 129.1, 125.9, 117.3, 115.7, 91.7, 90.9, 81.1, 61.4, 15.2. NMR data were in close agreement with the literature[2].

1-(4-(3, 3-diethoxyprop-1-yn-1-yl) phenyl) ethenone (3u)

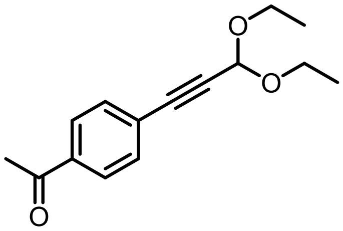

Colourless oil, yield 30%, **^{1}H NMR** (400 MHz, $CDCl_3$) δ 7.91 (d, J = 8.4 Hz, 2H), 7.56 (d, J = 8.4 Hz, 2H), 5.51 (s, 1H), 3.83 (m, 2H), 3.68 (m, 2H), 2.60 (s, 3H), 1.29 (t, J = 7.2 Hz, 6H). **^{13}C NMR** (100 MHz, $CDCl_3$) δ 197.4, 136.8, 132.2, 128.3, 126.8, 91.8, 87.6, 84.3, 61.2, 26.8, 15.2. NMR data were in close agreement with the literature[2].

methyl 4-(3, 3-diethoxyprop-1-yn-1-yl) benzoate (3v)

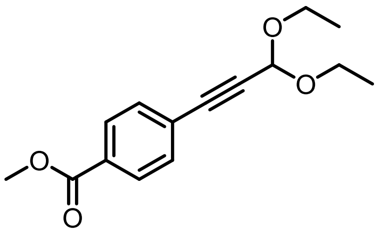

Colourless oil, yield 45%, **^{1}H NMR** (400 MHz, $CDCl_3$) δ 7.97 (d, J = 8.4 Hz, 2H), 7.52 (d, J = 8.4 Hz, 2H), 5.49 (s, 1H), 3.90 (s, 3H), 3.84 – 3.78 (m, 2H), 3.70 – 3.61 (m, 2H), 1.26 (t, J = 7.2 Hz, 6H) **^{13}C NMR** (100 MHz, $CDCl_3$) δ 166.5, 132.0, 130.2, 129.5, 126.6, 91.8, 87.3, 84.4, 61.2, 52.4, 15.2. NMR data were in close agreement with the literature[2].

1, 3-dichloro-5-(3, 3-diethoxyprop-1-yn-1-yl) benzene (3w)

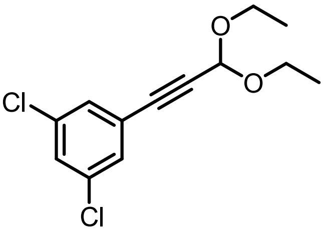

Colourless oil, yield 40%, **^{1}H NMR** (400 MHz, $CDCl_3$) δ 7.34 (d, J = 1.6 Hz, 2H), 7.33 – 7.32 (m, 1H), 5.46 (s, 1H), 3.84 – 3.75 (m, 2H), 3.65 (m, 2H), 1.27 (t, J = 7.2 Hz, 6H). **^{13}C NMR** (100 MHz, $CDCl_3$) δ 135.0, 130.2, 129.3, 124.8, 91.6, 86.9, 82.5, 61.2, 15.2. The compound is new compound.

3-(3, 3-diethoxyprop-1-yn-1-yl) thiophene (3x)

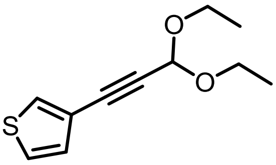

Yellowish oil, yield 34%, **^{1}H NMR** (400 MHz, $CDCl_3$) δ 7.52 (dd, J = 3.2, 1.6 Hz, 1H), 7.27 (dd, J = 3.2, 1.6 Hz, 1H), 7.15 (dd, J = 5.2, 1.2 Hz, 1H), 5.50 (d, J = 8.8 Hz, 1H), 3.83 (m, 2H), 3.67 (m, 2H), 1.29 (t, J = 7.2 Hz, 6H). **^{13}C NMR** (100 MHz, $CDCl_3$) δ 130.0, 130.0, 125.5, 121.0, 91.9, 84.1, 80.6, 61.1, 15.3. NMR data were in close agreement with the literature[2].

3-(3, 3-diethoxyprop-1-yn-1-yl) pyridine (3y)

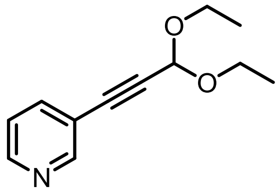

Yellow oil, yield 56%, **^{1}H NMR** (400 MHz, $CDCl_3$) δ 8.69 (s, 1H), 8.53 (s, 1H), 7.73 (d, J = 8.0 Hz, 1H), 7.23 (dd, J = 7.6, 4.8 Hz, 1H), 5.47 (s, 1H), 3.83 – 3.75 (m, 2H), 3.68 – 3.63 (m, 2H), 1.25 (t, J = 7.2 Hz, 6H). **^{13}C NMR** (100 MHz, $CDCl_3$) δ 152.6, 149.2, 138.9, 123.1, 119.2, 91.7, 87.9, 81.9, 61.2, 15.2. NMR data were in close agreement with the literature[2].

2-bromo-5-(3, 3-diethoxyprop-1-yn-1-yl)-1-methyl-1*H*-indole (3z)

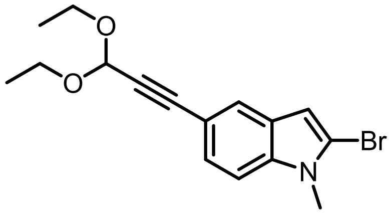

Colourless oil, yield 55%, **^{1}H NMR** (400 MHz, $CDCl_3$) δ 7.73 (s, 1H), 7.36 (d, J = 8.4 Hz, 1H), 7.22 (d, J = 8.5 Hz, 1H), 7.08 (s, 1H), 5.53 (s, 1H), 3.90 – 3.82 (m, 2H), 3.76 (s, 3H), 3.73 – 3.64 (m, 2H), 1.29 (t, J = 7.2 Hz, 6H). **^{13}C NMR** (100 MHz, $CDCl_3$) δ 136.2, 128.8, 127.2, 126.5, 123.9, 113.5, 109.7, 92.1, 89.8, 86.5, 82.8, 61.0, 33.3, 15.3. The compound is new compound.

1-((allyloxy)methyl)-2-(3, 3-diethoxyprop-1-yn-1-yl) benzene (3aa)

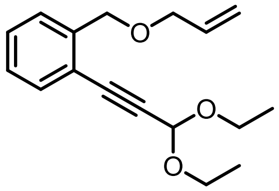

Light pink oil, yield 43%, ^{1}H NMR (400 MHz, $CDCl_3$) δ 7.48 (t, J = 6.8 Hz, 2H), 7.35 (t, J = 7.6 Hz, 1H), 7.23 (t, J = 7.6 Hz, 1H), 5.98 (ddd, J = 22.8, 10.8, 5.6 Hz, 1H), 5.52 (s, 1H), 5.37 – 5.29 (m, 1H), 5.21 (dd, J = 10.4, 1.2 Hz, 1H), 4.69 (s, 2H), 4.09 (dt, J = 5.6, 1.2 Hz, 2H), 3.82 (m, 2H), 3.67 (m, 2H), 1.28 (t, J = 7.2 Hz, 6H). **^{13}C NMR** (100 MHz, $CDCl_3$) δ 140.7, 134.8, 132.6, 129.2, 127.5, 127.3, 120.5, 117.2, 92.0, 89.1, 82.9, 71.7, 70.2, 61.1, 15.3. The compound is new compound.

(8*R*,9*S*,13*S*,14*S*)-3-(3-ethoxy-4-methoxybut-1-yn-1-yl)-13-methyl-6,7,8,9,11,12,13,14,15,16-decahydro-17*H*-cyclopenta[*a*]phenanthren-17-one (3ab)

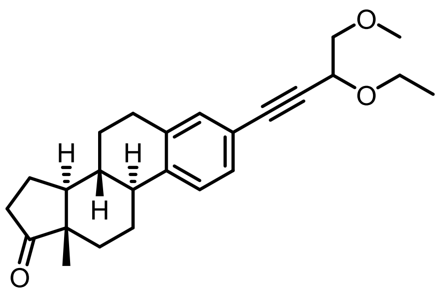

Yellowish oil, yield 27%,

^{1}H NMR (400 MHz, $CDCl_3$) δ 7.26 (s, 1H), 7.23 (d, J = 3.6 Hz, 2H), 5.48 (s, 1H), 3.82 (tt, J = 14.4, 7.2 Hz, 2H), 3.71 – 3.61 (m, 2H), 2.88 (dd, J = 8.8, 4.0 Hz, 2H), 2.51 (dd, J = 18.8, 8.8 Hz, 1H), 2.44 – 2.36 (m, 1H), 2.29 (t, J = 8.4 Hz, 1H), 2.16 (dd, J = 18.4, 9.2 Hz, 1H), 2.11 – 1.94 (m, 3H), 1.69 – 1.62 (m, 1H), 1.49 (tdd, J = 24.8, 15.2, 9.6 Hz, 5H), 1.27 (t, J = 7.2 Hz, 6H), 0.91 (s, 3H). **^{13}C NMR** (100 MHz, $CDCl_3$) δ 220.9, 140.9, 136.7, 132.6, 129.4, 125.5, 119.3, 92.0, 85.5, 83.9, 61.0, 50.6, 48.1, 44.6, 38.1, 36.0, 31.7, 29.2, 26.4, 25.7, 21.7, 15.3, 14.0. The compound is new compound.

3-(3,3-diethoxyprop-1-yn-1-yl) phenyl palmitate (3ac)

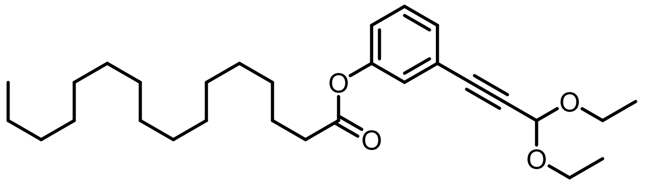

Colourless oil, yield 30%,**^{1}H NMR** (400 MHz, $CDCl_3$) δ 7.31 (d, J = 8.0 Hz, 2H), 7.20 (s, 1H), 7.06 (d, J = 6.4 Hz, 1H), 5.47 (s, 1H), 3.86 – 3.76 (m, 2H), 3.70 – 3.61 (m, 2H), 2.54 (t, J = 7.6 Hz, 2H), 1.79 – 1.70 (m, 2H), 1.26 (s, 30H), 0.88 (t, J = 6.4 Hz, 3H).**^{13}C NMR** (100 MHz, $CDCl_3$) δ 172.1, 150.6, 129.3, 129.3, 125.2, 123.3, 122.5, 91.8, 85.3, 84.3, 61.1, 34.5, 32.1, 29.7, 29.7, 29.7, 29.6, 29.5, 29.4, 29.3, 29.1, 25.0, 22.8, 15.2, 14.3. The compound is new compound.

3-(m-tolyl)propiolaldehyde (3ad)

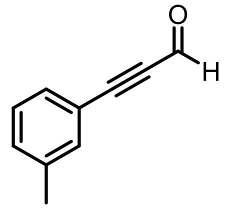

Brown oil, yield 48%, ^{1}H NMR (400 MHz, $CDCl_3$) δ 9.74 (s, 1H), 7.77 – 7.71 (m, 2H), 7.62 (d, J = 5.2 Hz, 2H), 2.69 (s, 3H). **^{13}C NMR** (100 MHz, $CDCl_3$) δ 176.9, 138.7, 133.9, 132.4, 130.6, 128.7, 119.3, 95.7, 88.4, 21.3. NMR data were in close agreement with the literature[3].

3-(4-(tert-butyl)phenyl)propiolaldehyde (3ae)

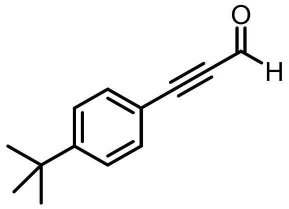

Brown oil, yield 45%, **^{1}H NMR** (400 MHz, $CDCl_3$) δ 9.41 (s, 1H), 7.55 (d, J = 8.4 Hz, 2H), 7.43 (d, J = 8.4 Hz, 2H), 1.33 (s, 9H). **^{13}C NMR** (100 MHz, $CDCl_3$) δ 177.0, 155.3, 133.4, 126.0, 116.4, 96.1, 88.6, 35.3, 31.1. The compound is new compound.

8 Another proposed mechanism.

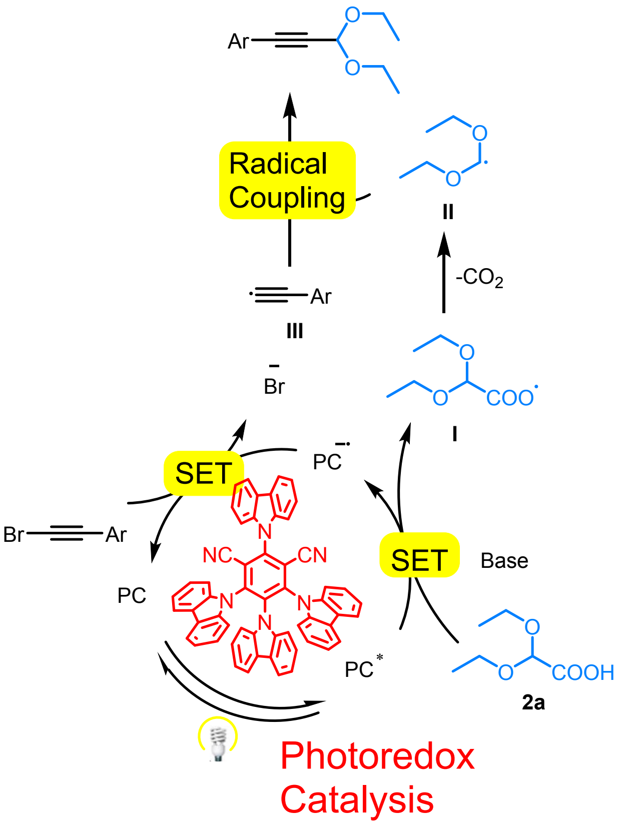

Scheme S1. Another proposed mechanism

The other plausible mechanism for the reaction is shown in Scheme S1. The metal-free photocatalyst 4CzIPN is irradiated to its photoexcited state 4CzIPN* under 5W blue LEDs. With the help of Cs_2CO_3, the readily available 2, 2-diethoxyacetic acid [Eox (2a Cs salt) = +0.95V vs. SCE][4] is easily oxidized to produce carboxyl radical intermediate **I** by 4CzIPN* due to its featured higher redox potential [E ($PC^*/PC^{-\cdot}$) = +1.35V vs. SCE][5]. **I** then removes one molecule of CO_2 to form a 2, 2-diethoxyacetal radical **II**. At the same time, arylethynyl bromide undergoes a SET process to produce an arylethynyl radical **III**[6] and a bromine anion by the photocatalyst in a reduced state [E (PC/PC-·) = -1.21 V vs. SCE][4], and the photocatalyst completes the cycle. Finally, the radical–radical cross-coupling between **II** and **III** provides the cross-coupling product.

9. References

1. Z.-S. Ye, M.-C. Liu, B.-D. Lin, H.-Y. Wu, J.-C. Ding, J. Cheng. *Tetrahedron Lett.* **2009**, *50*, 530.

2. M. Lemhadri, H. Doucet, M. Santelli. *Tetrahedron* **2005**, *61*, 9839.

3. S. Belot, K. A. Vogt, C. Besnard, N. Krause, A. Alexakis. *Angew. Chem. Int. Ed.* **2009**, *48*, 8923.

4. H. Huang, X.-M. Li, C.-G. Yu, Y.-T. Zhang, P. S. Mariano and W. Wang *Angew. Chem., Int. Ed.* **2017**, *56*, 1500-1505.

5. J. Luo and J. Zhang, *ACS Catal.* **2016**, *6*, 873-877.

6. (a) Y.-C. Zhao, J.-W. Jin and P. W. H. Chan, *Adv. Synth. Catal.* 2019, 361, 1313-1321. (b) K. Ni, L.-G. Meng, K. Wang and L. Wang, *Org. Lett.* **2018**, *20*, 2245−2248.

10. Copies of ^{1}H NMR ^{13}C NMR and ^{19}F NMR spectra for compounds

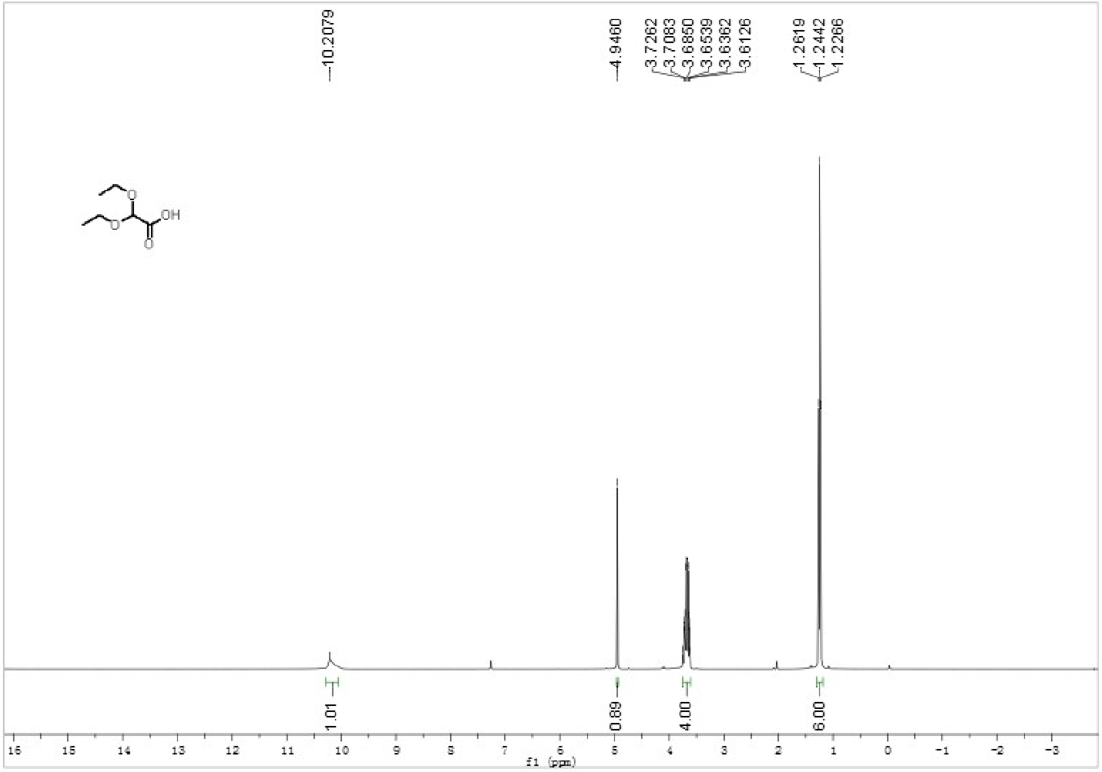

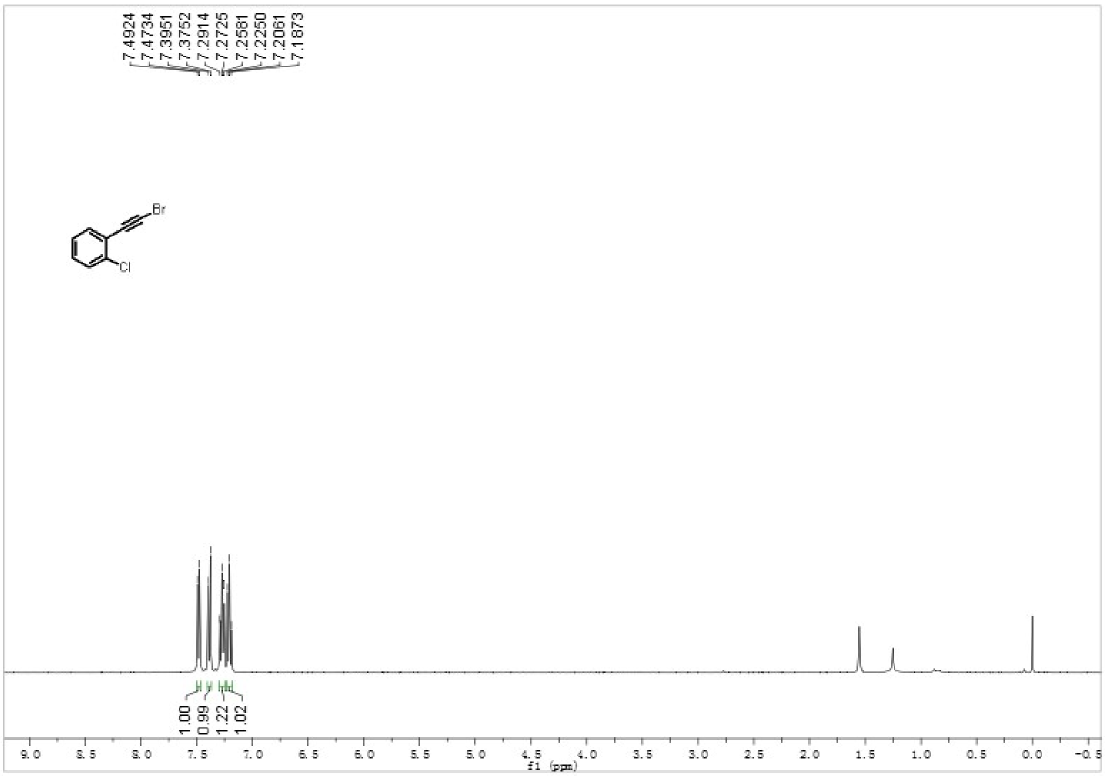

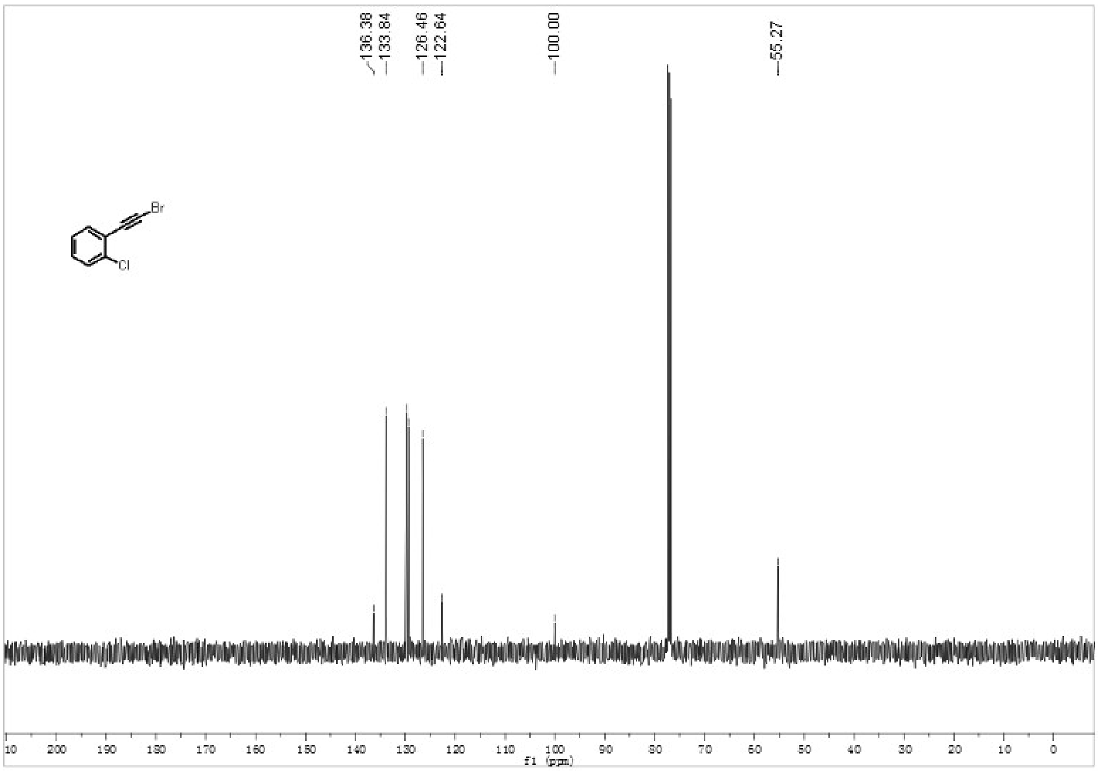
136.38
133.84
126.46
122.64
100.00
55.27
Br
Cl
f1 (ppm)

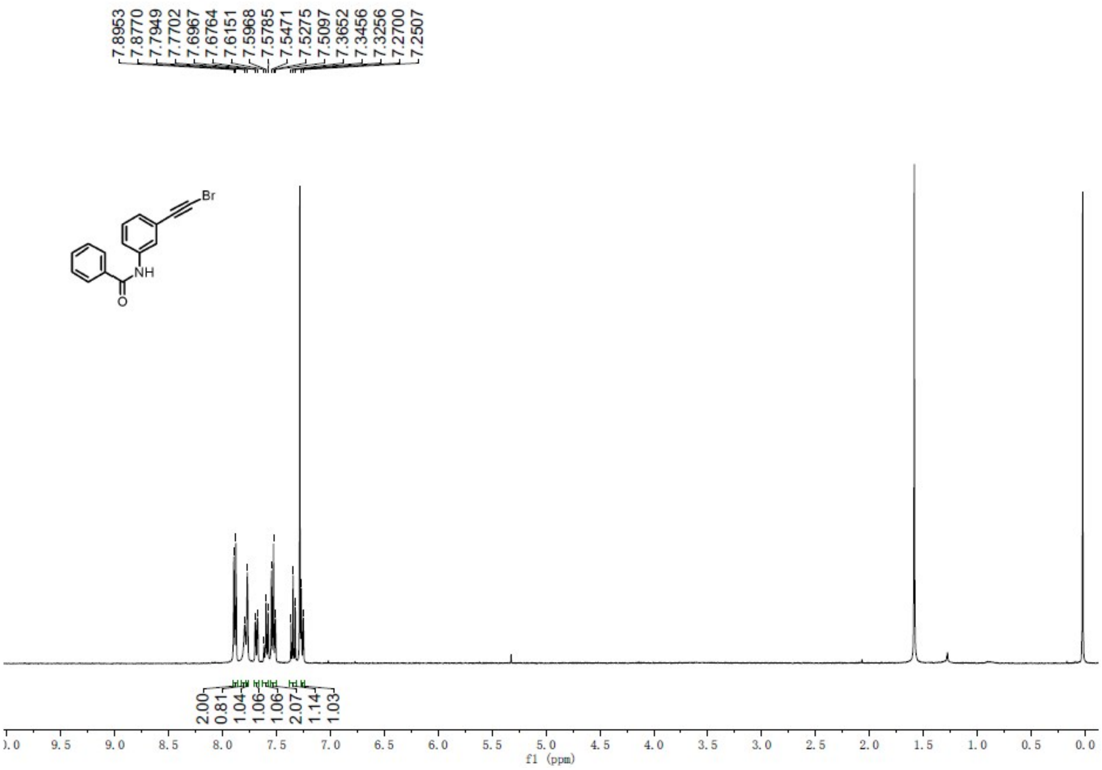
7.8953
7.8770
7.7949
7.7702
7.6967
7.6764
7.6151
7.5968
7.5785
7.5471
7.5275
7.5097
7.3652
7.3456
7.3256
7.2700
7.2507
Br
NH
O
2.00
0.81
1.04
1.06
1.06
2.07
1.14
1.03
f1 (ppm)

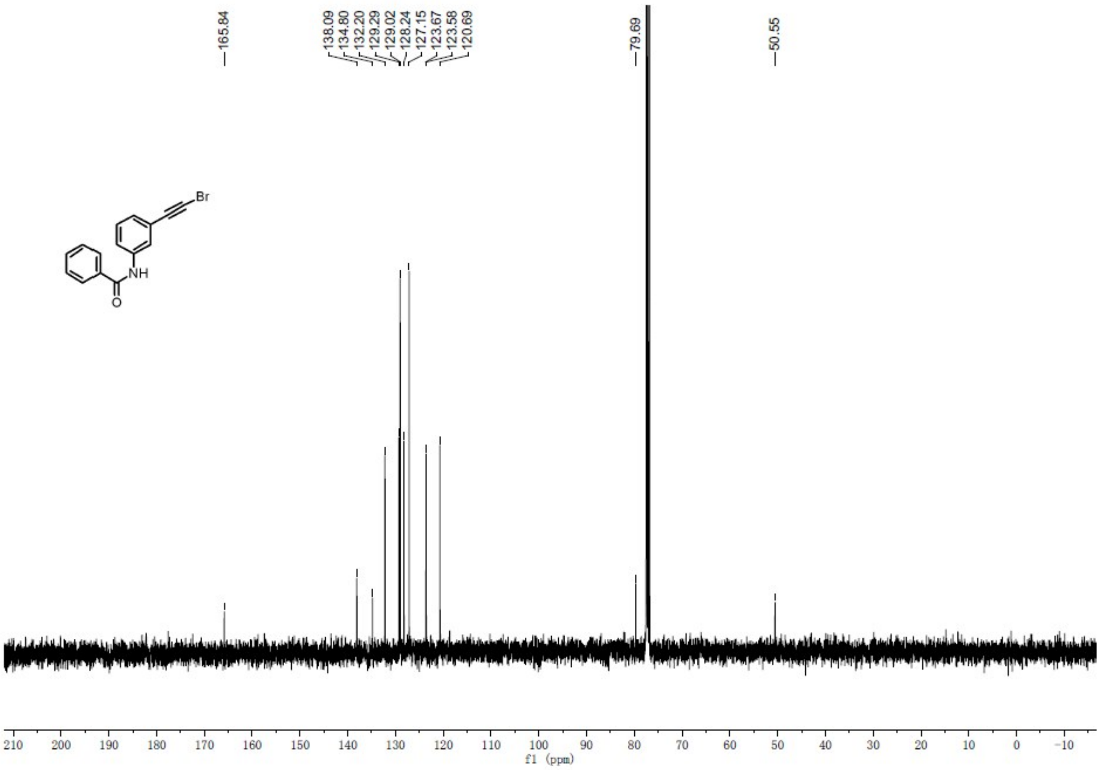
165.84
138.09
134.80
132.20
129.29
129.02
128.24
127.15
123.67
123.58
120.69
79.69
50.55
Br
NH
O
f1 (ppm)

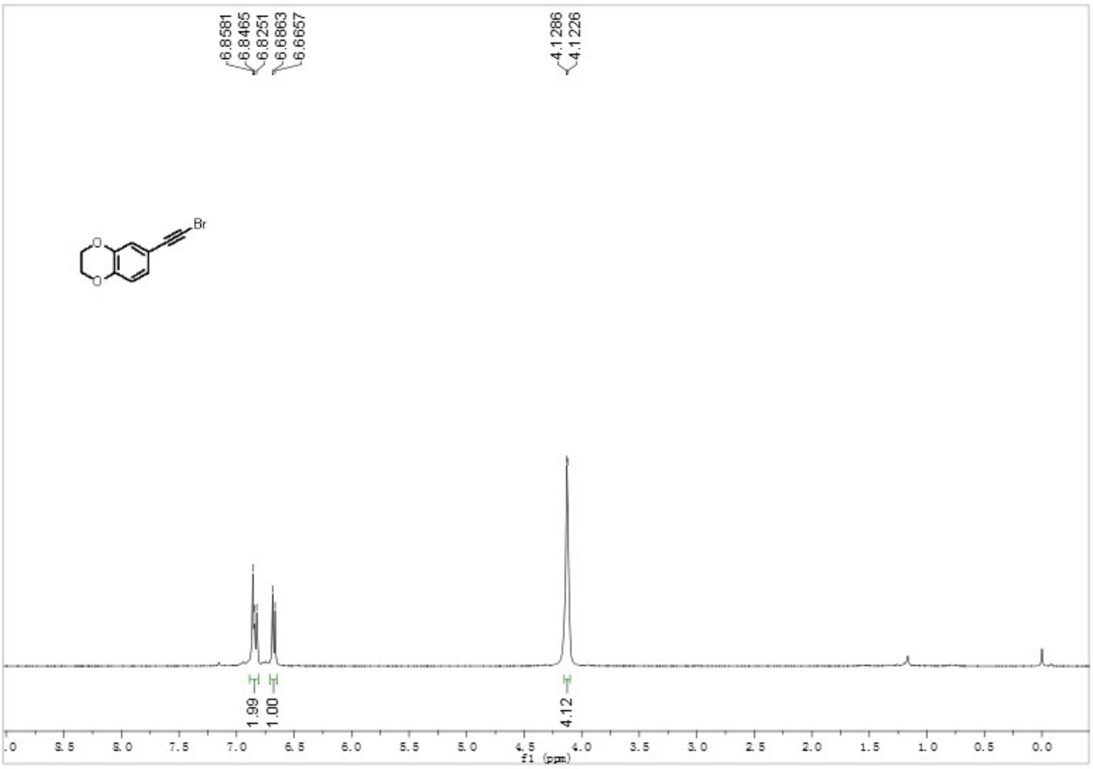
6.8581
6.8465
6.8251
6.6863
6.6657
4.1286
4.1226
Br
O
O
1.99
1.00
4.12
f1 (ppm)

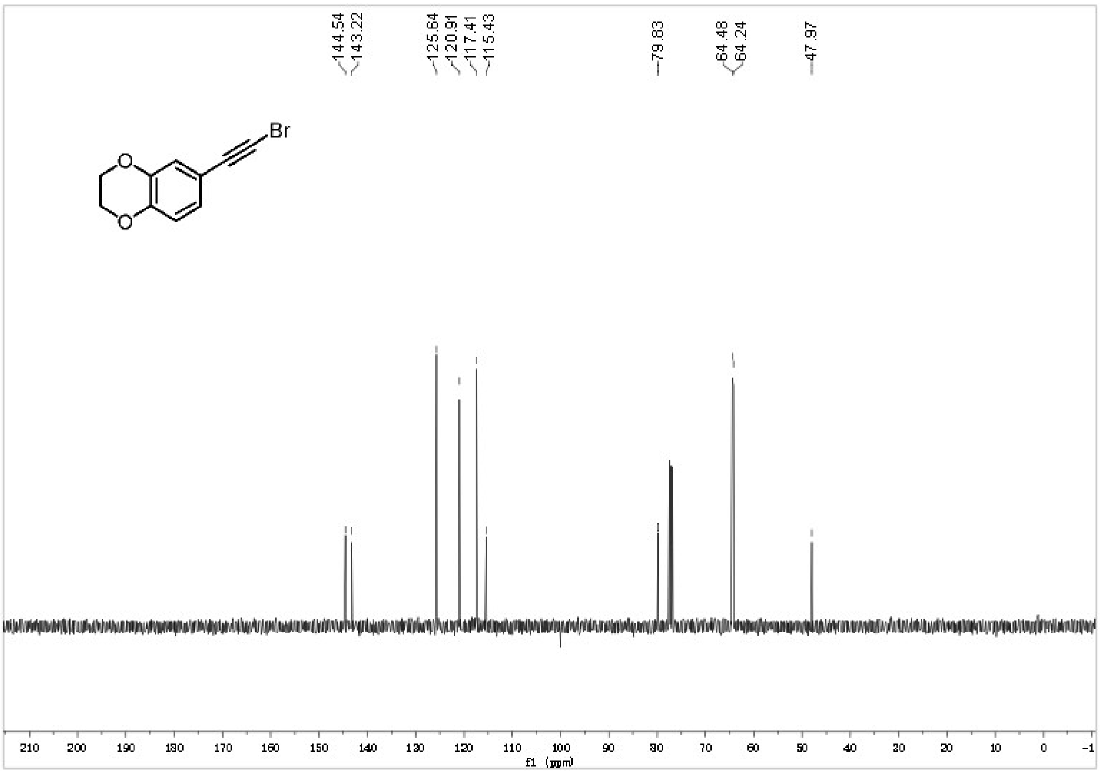
144.54
143.22
125.64
120.91
117.41
115.43
79.83
64.48
64.24
47.97
Br
O
O
f1 (ppm)

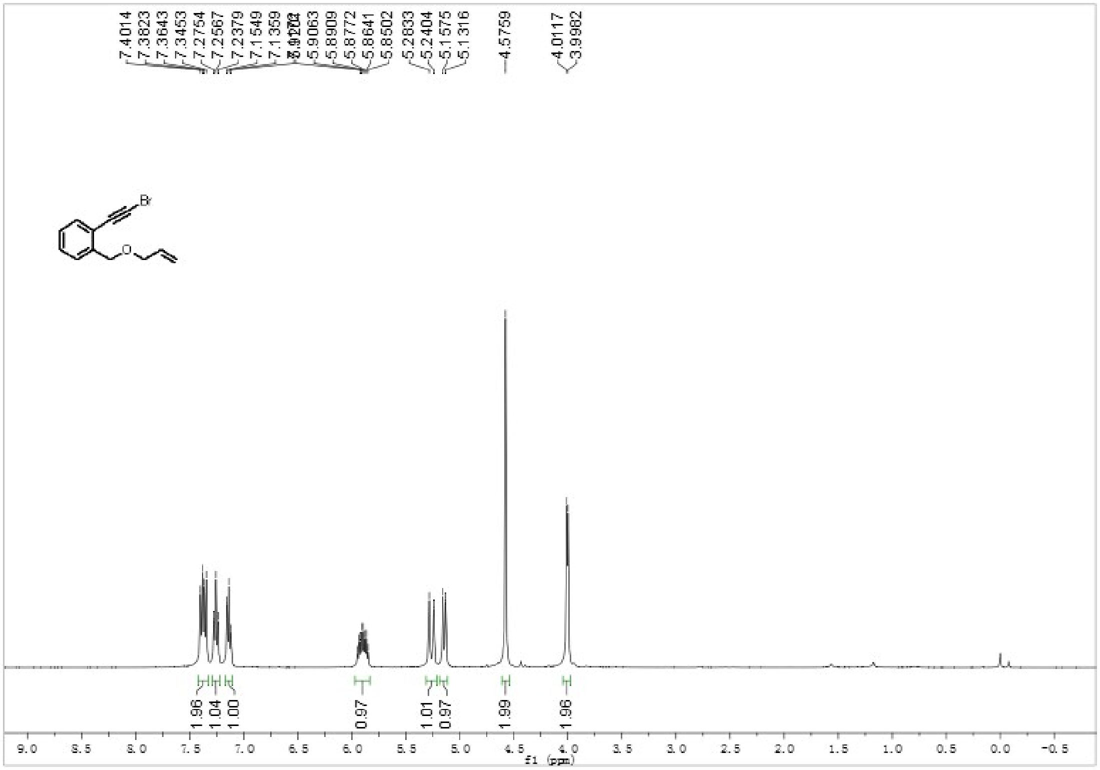
7.4014
7.3823
7.3643
7.3453
7.2754
7.2567
7.2379
7.1549
7.1359
5.9063
5.8909
5.8772
5.8641
5.8502
5.2833
5.2404
5.1575
5.1316
4.5759
4.0117
3.9982
Br
O
1.96
1.04
1.00
0.97
1.01
0.97
1.99
1.96
f1 (ppm)

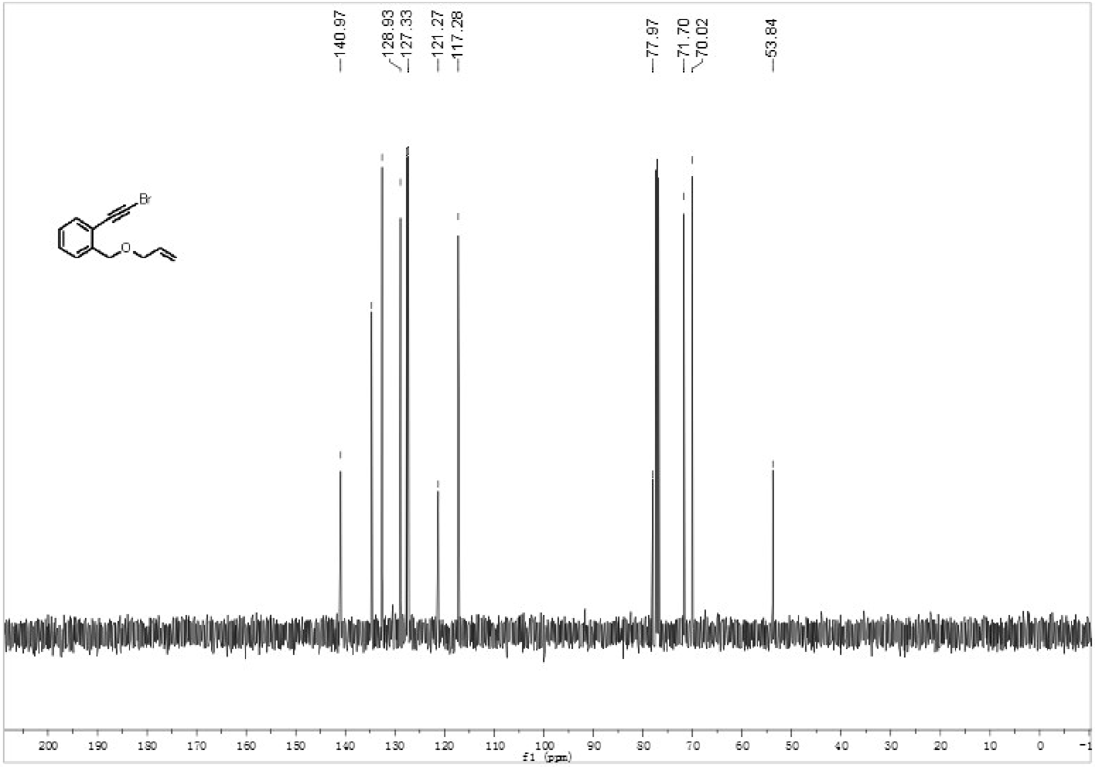

140.97
128.93
127.33
121.27
117.28
77.97
71.70
70.02
53.84
Br
O
200
190
180
170
160
150
140
130
120
110
100
90
80
70
60
50
40
30
20
10
0
f1 (ppm)

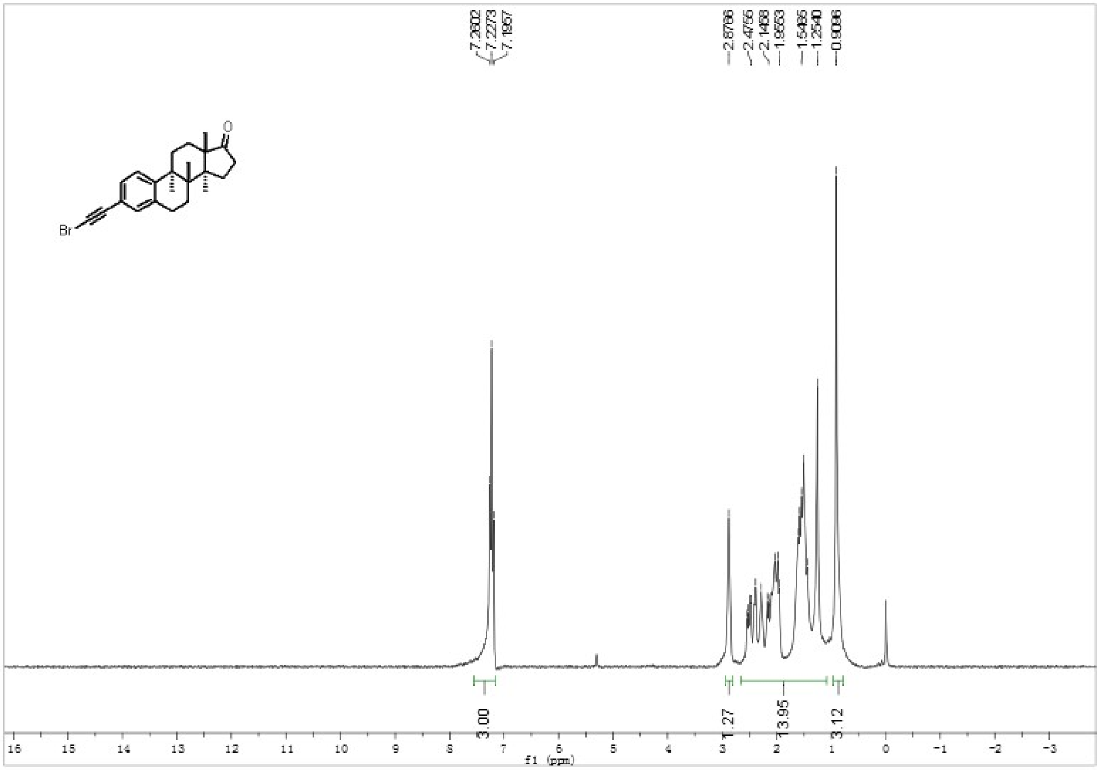

7.2602
7.2273
7.1957
2.8766
2.4755
2.1468
1.9553
1.5436
1.2540
0.9006
O
Br
3.00
1.27
13.95
3.12
16
15
14
13
12
11
10
9
8
7
6
5
4
3
2
1
0
-1
-2
-3
f1 (ppm)

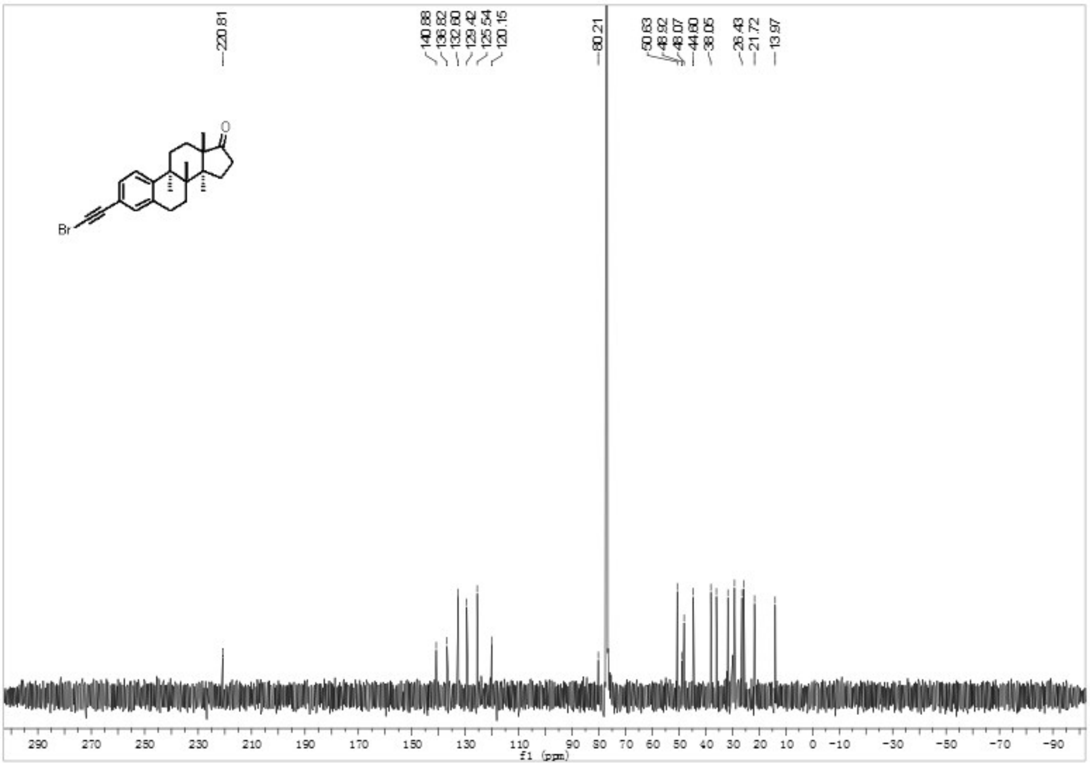
220.81
140.88
136.82
132.60
129.42
125.54
120.15
80.21
50.63
48.92
48.07
44.60
38.05
26.43
21.72
13.97
Br
O
f1 (ppm)

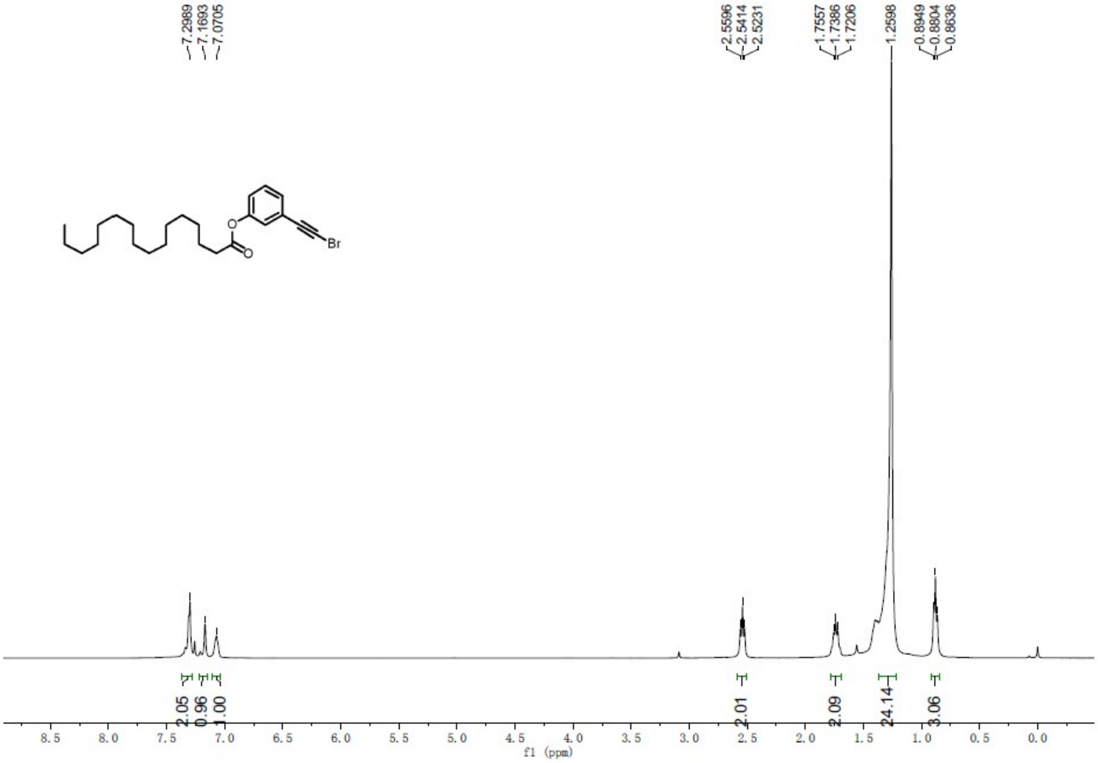
7.2989
7.1693
7.0705
2.5596
2.5414
2.5231
1.7557
1.7386
1.7206
1.2598
0.8949
0.8804
0.8636
O
Br
2.05
0.96
1.00
2.01
2.09
24.14
3.06
f1 (ppm)

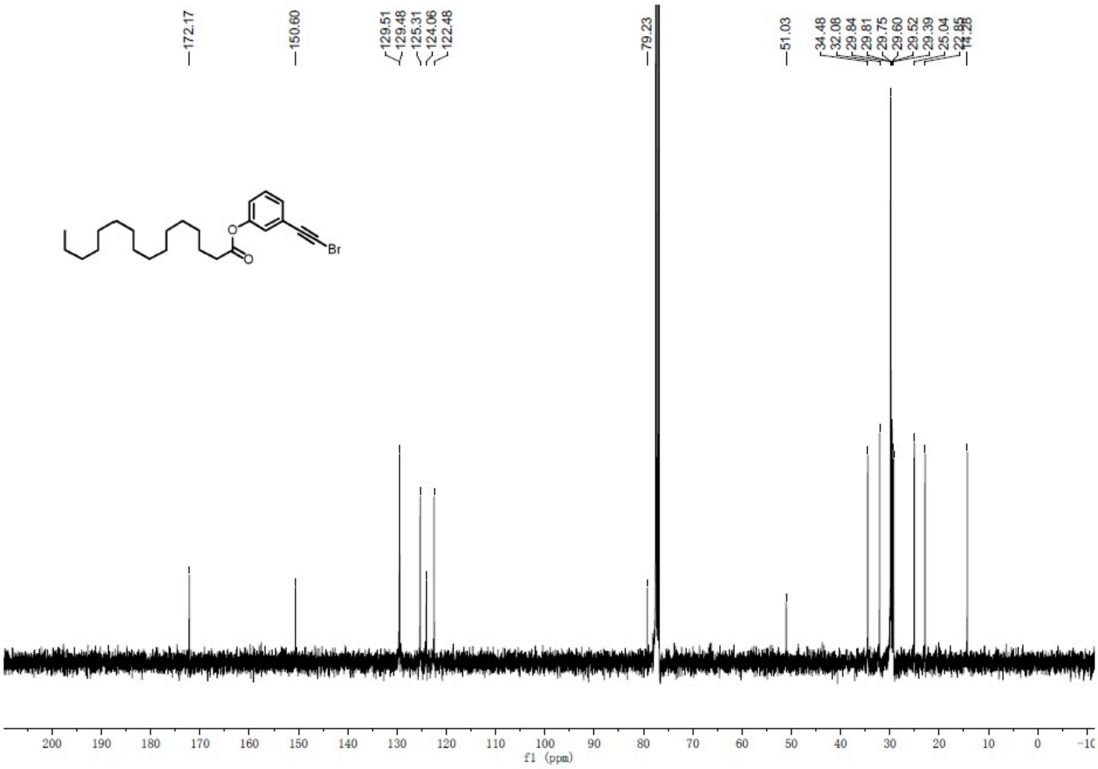
172.17
150.60
129.51
129.48
125.31
124.06
122.48
79.23
51.03
34.48
32.08
29.84
29.81
29.75
29.60
29.52
29.39
25.04
Br
O
f1 (ppm)

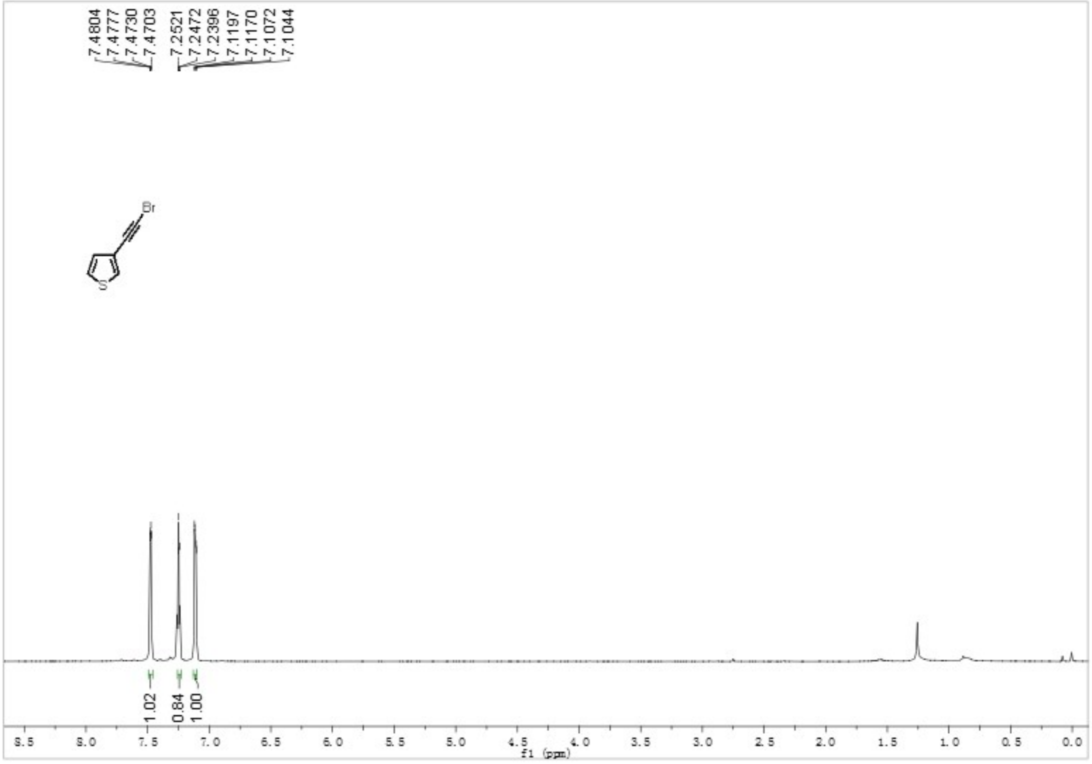
7.4804
7.4777
7.4730
7.4703
7.2521
7.2472
7.2396
7.1197
7.1170
7.1072
7.1044
Br
S
1.02
0.84
1.00
f1 (ppm)

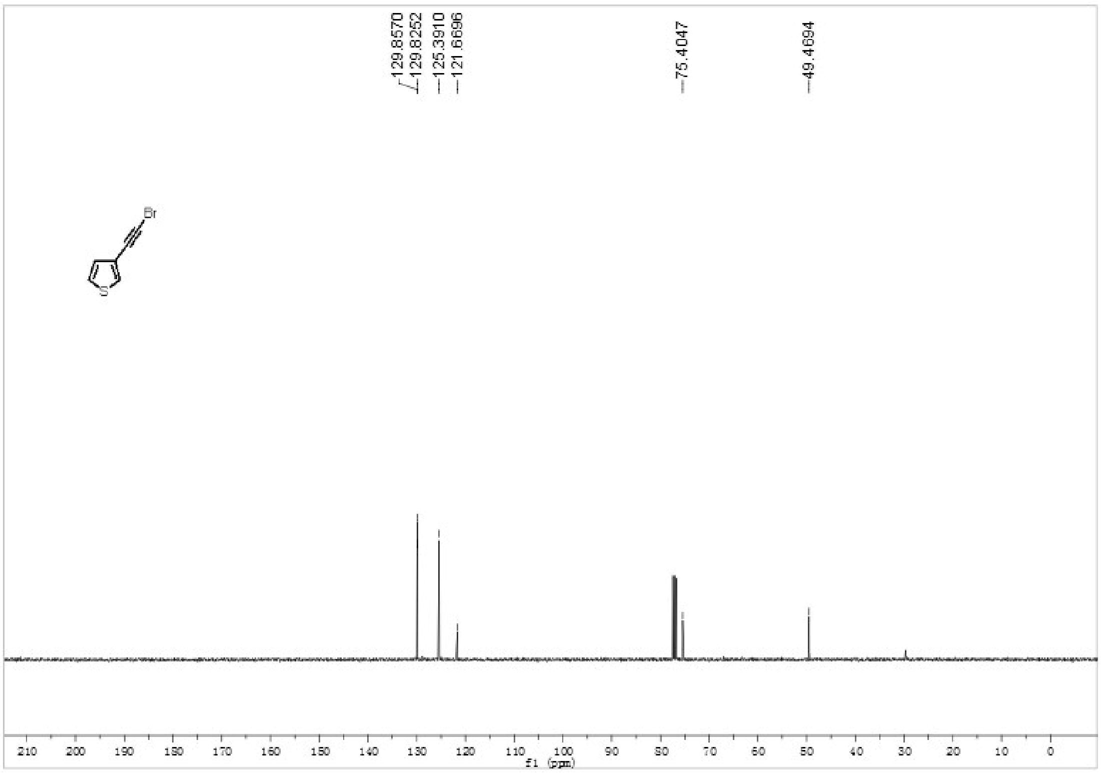
129.8570
129.8252
125.3910
121.6696
75.4047
49.4694
Br
S
f1 (ppm)

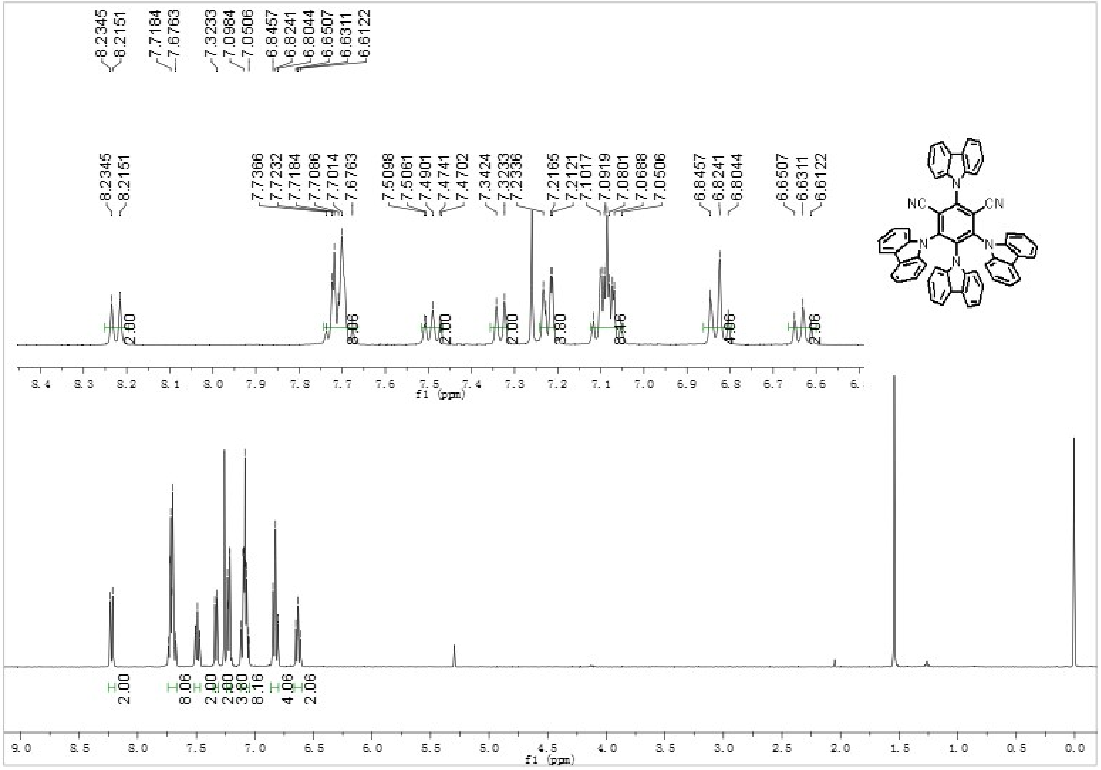
8.2345
8.2151
7.7184
7.6763
7.3233
7.0984
7.0506
6.8457
6.8241
6.8044
6.6507
6.6311
6.6122
7.7366
7.7232
7.7184
7.7086
7.7014
7.6763
7.5098
7.5061
7.4901
7.4741
7.4702
7.3424
7.3233
7.2336
7.2165
7.2121
7.1017
7.0919
7.0801
7.0688
7.0506
NC
CN
N
2.00
8.06
2.00
2.00
3.80
8.16
4.06
2.06
f1 (ppm)

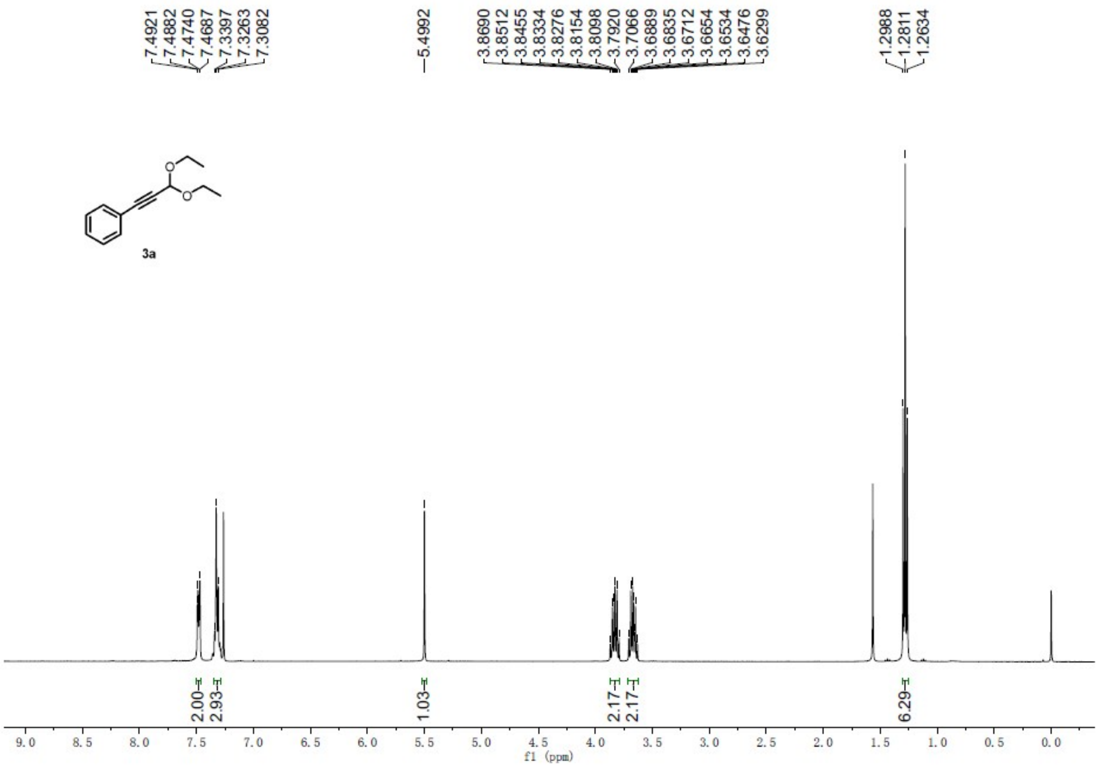
7.4921
7.4882
7.4740
7.4687
7.3397
7.3263
7.3082
5.4992
3.8690
3.8512
3.8455
3.8334
3.8276
3.8154
3.8098
3.7920
3.7066
3.6889
3.6835
3.6712
3.6654
3.6534
3.6476
3.6299
1.2988
1.2811
1.2634
3a
2.00
2.93
1.03
2.17
2.17
6.29
f1 (ppm)

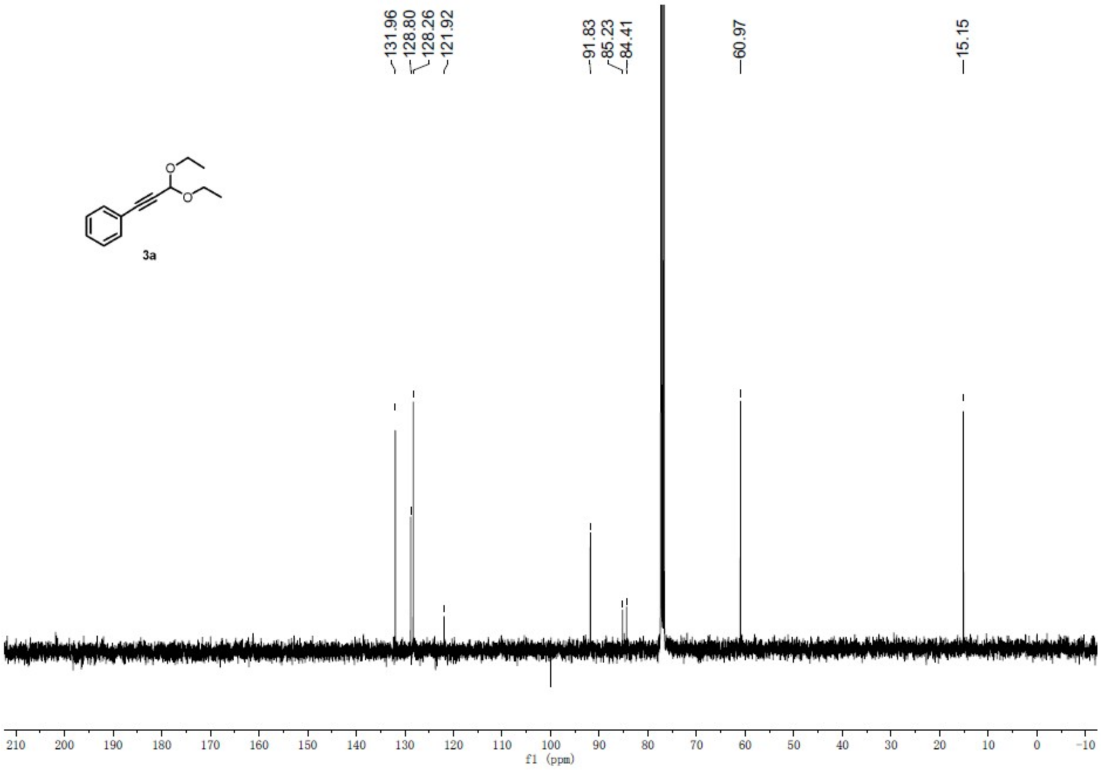
131.96
128.80
128.26
121.92
91.83
85.23
84.41
60.97
15.15
3a
f1 (ppm)

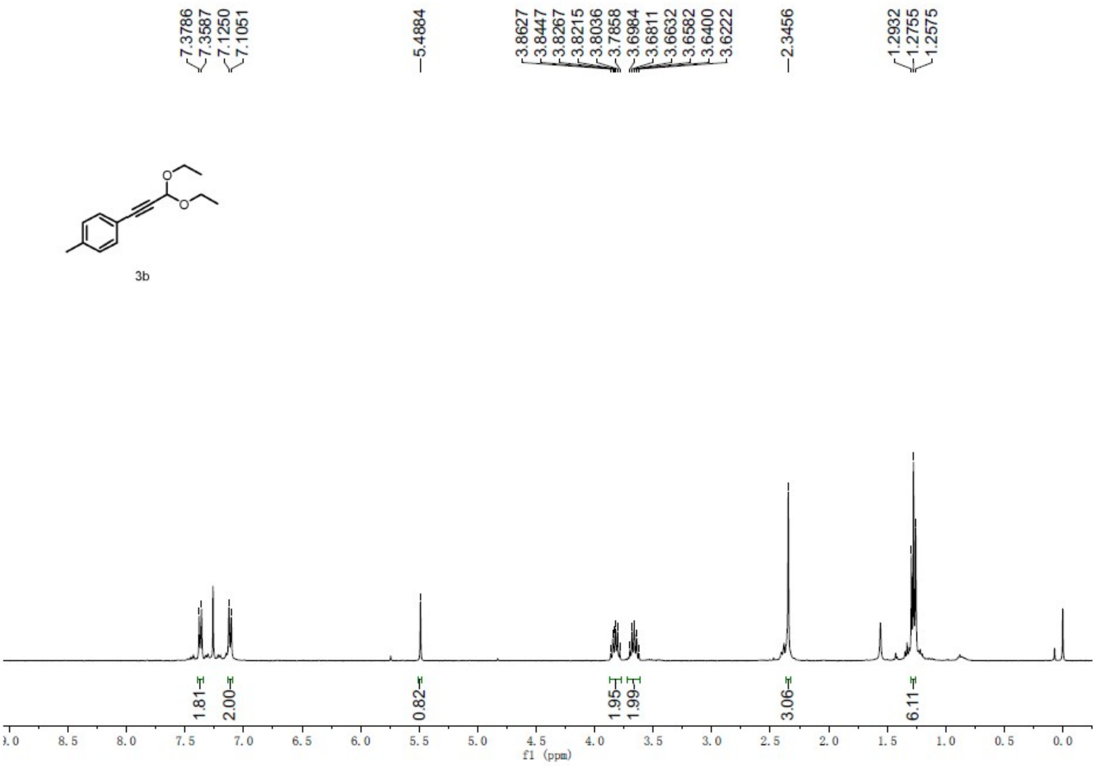
7.3786
7.3587
7.1250
7.1051
5.4884
3.8627
3.8447
3.8267
3.8215
3.8036
3.7858
3.6984
3.6811
3.6632
3.6582
3.6400
3.6222
2.3456
1.2932
1.2755
1.2575
3b
1.81
2.00
0.82
1.95
1.99
3.06
6.11
f1 (ppm)

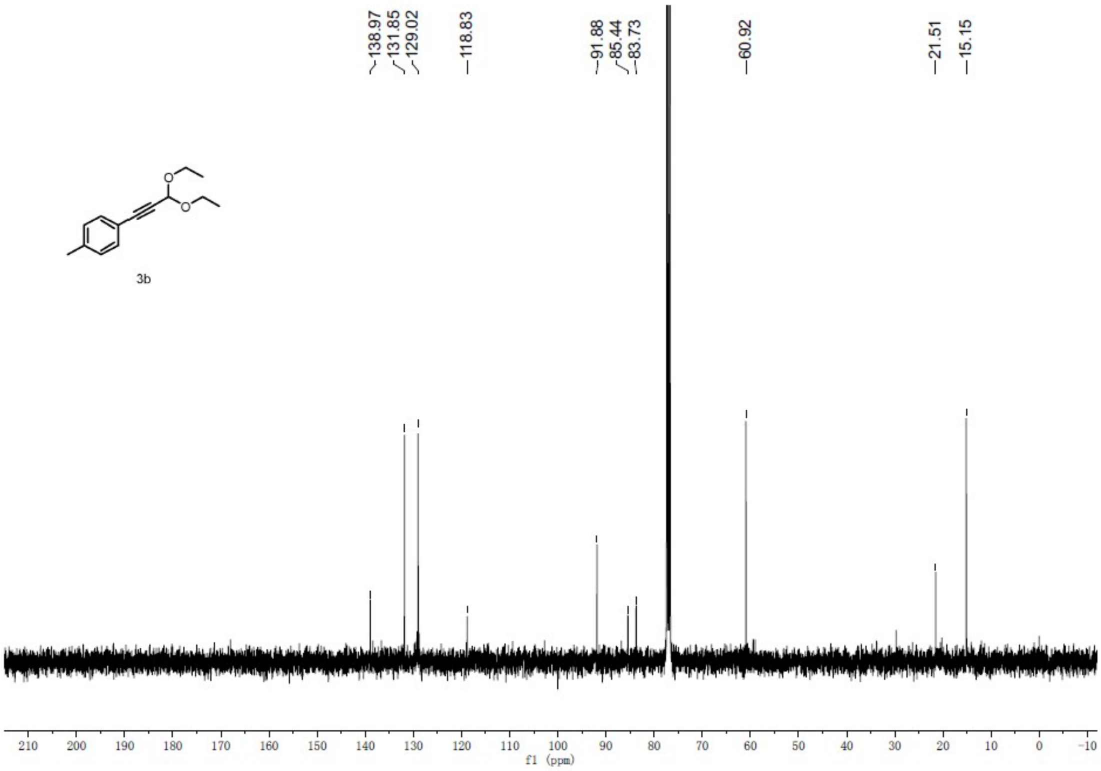
138.97
131.85
129.02
118.83
91.88
85.44
83.73
60.92
21.51
15.15
3b
210 200 190 180 170 160 150 140 130 120 110 100 90 80 70 60 50 40 30 20 10 0 -10
f1 (ppm)

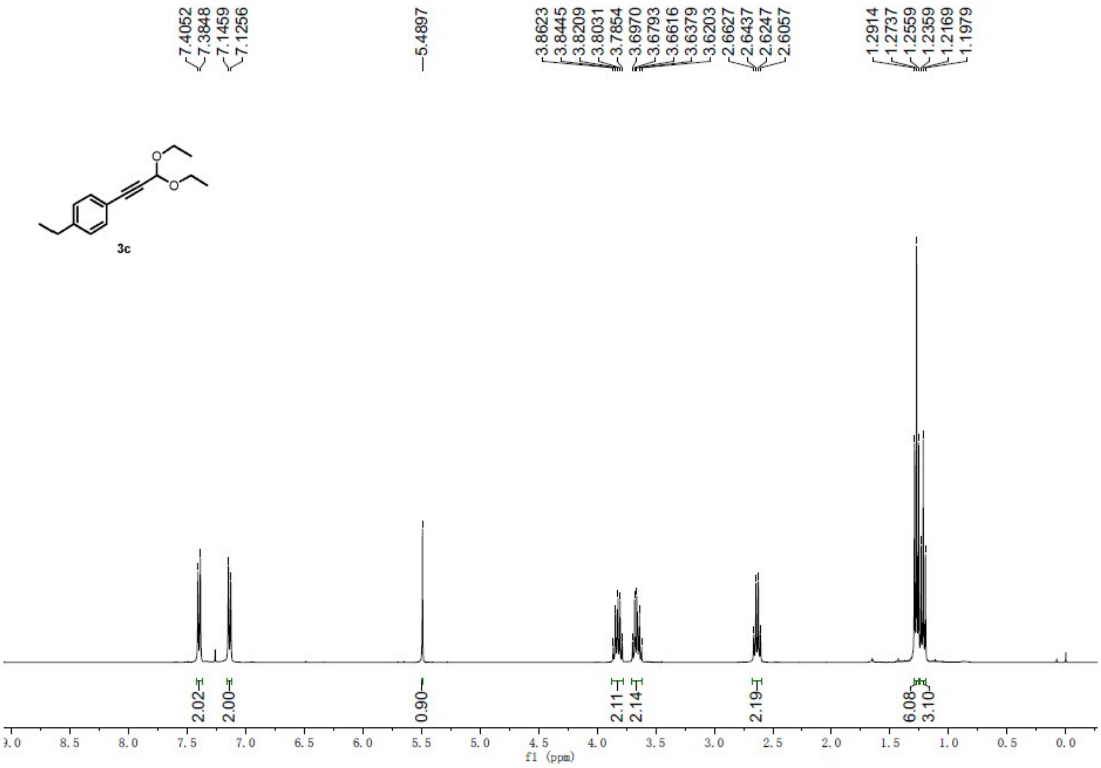
7.4052
7.3848
7.1459
7.1256
5.4897
3.8623
3.8445
3.8209
3.8031
3.7854
3.6970
3.6793
3.6616
3.6379
3.6203
2.6627
2.6437
2.6247
2.6057
1.2914
1.2737
1.2559
1.2359
1.2169
1.1979
3c
2.02
2.00
0.90
2.11
2.14
2.19
6.08
3.10
9.0 8.5 8.0 7.5 7.0 6.5 6.0 5.5 5.0 4.5 4.0 3.5 3.0 2.5 2.0 1.5 1.0 0.5 0.0
f1 (ppm)

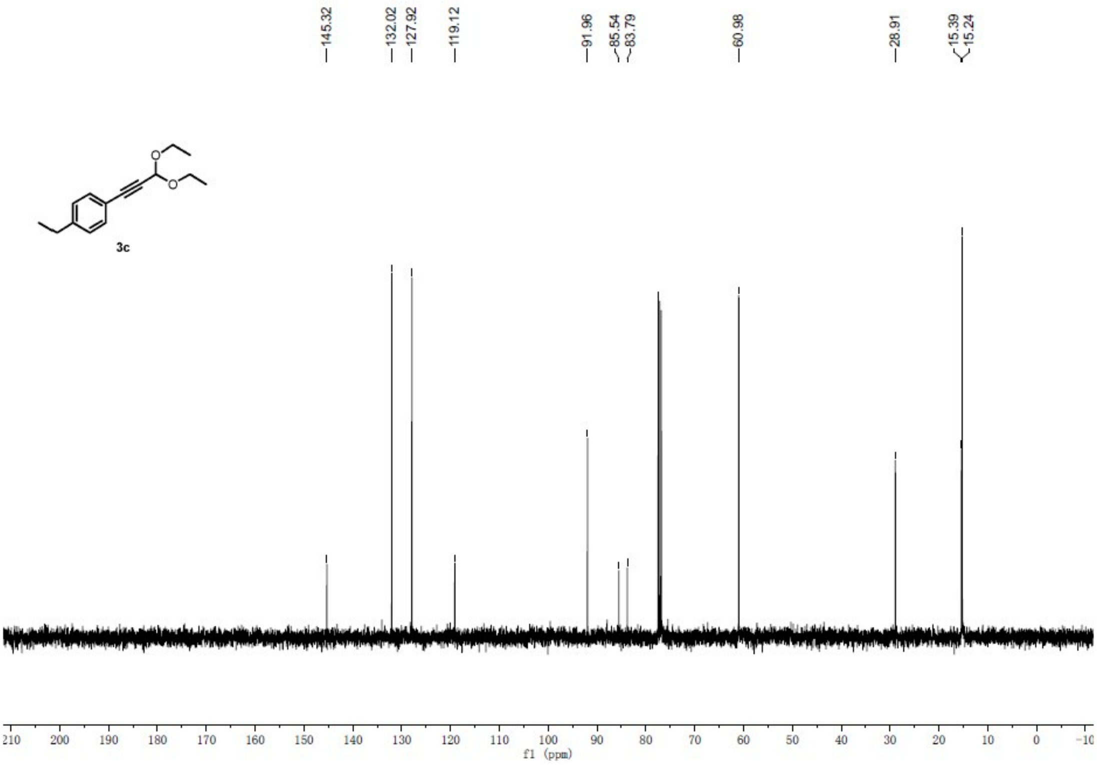
145.32
132.02
127.92
119.12
91.96
85.54
83.79
60.98
28.91
15.39
15.24
O
O
3c
f1 (ppm)

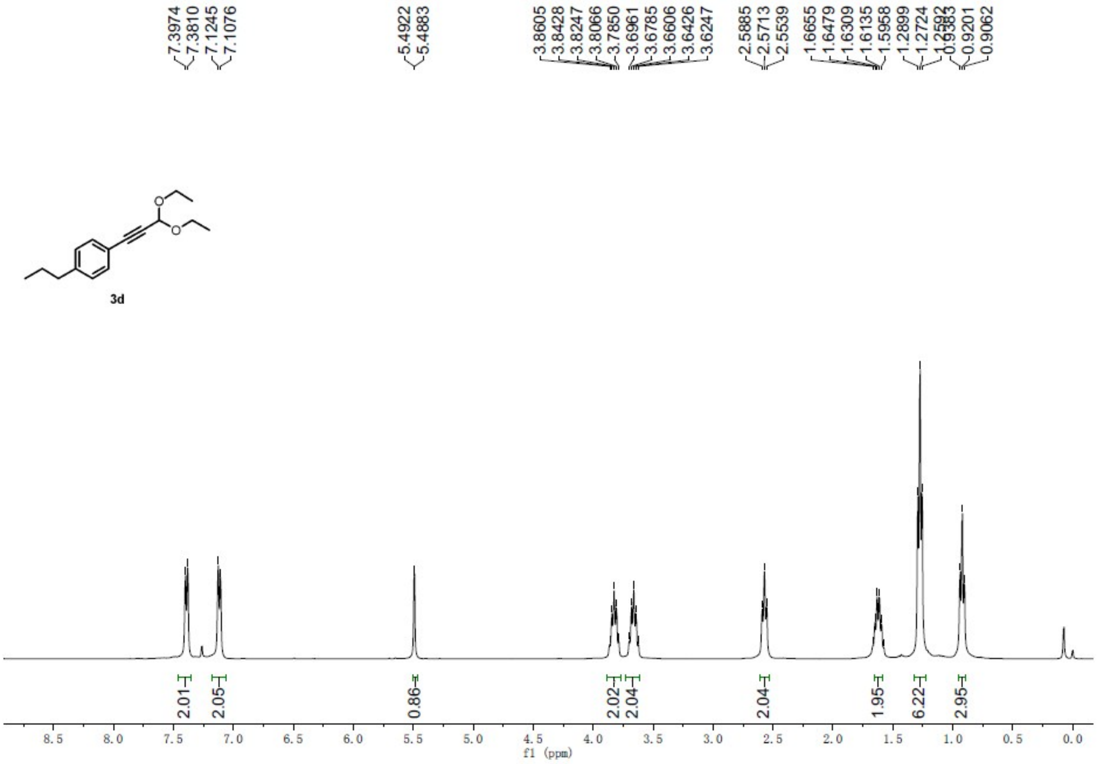
7.3974
7.3810
7.1245
7.1076
5.4922
5.4883
3.8605
3.8428
3.8247
3.8066
3.7850
3.6961
3.6785
3.6606
3.6426
3.6247
2.5885
2.5713
2.5539
1.6655
1.6479
1.6309
1.6135
1.5958
1.2899
1.2724
1.2592
0.9383
0.9201
0.9062
O
O
3d
2.01
2.05
0.86
2.02
2.04
2.04
1.95
6.22
2.95
f1 (ppm)

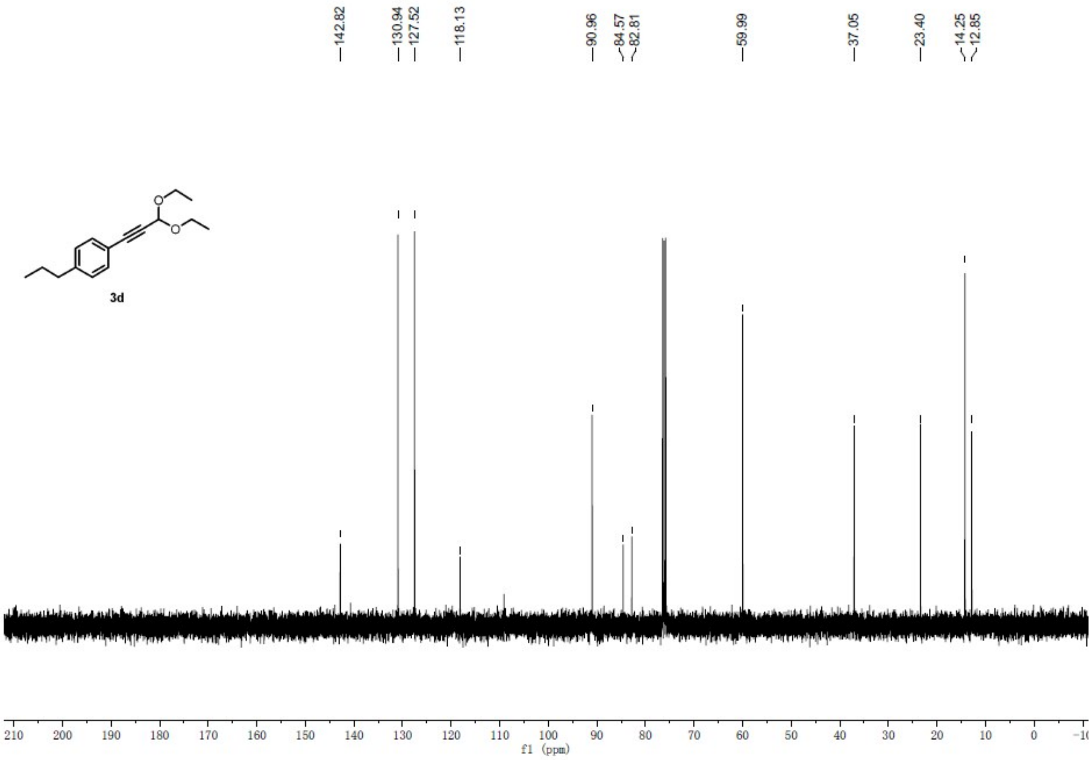
142.82
130.94
127.52
118.13
90.96
84.57
82.81
59.99
37.05
23.40
14.25
12.85
3d
f1 (ppm)

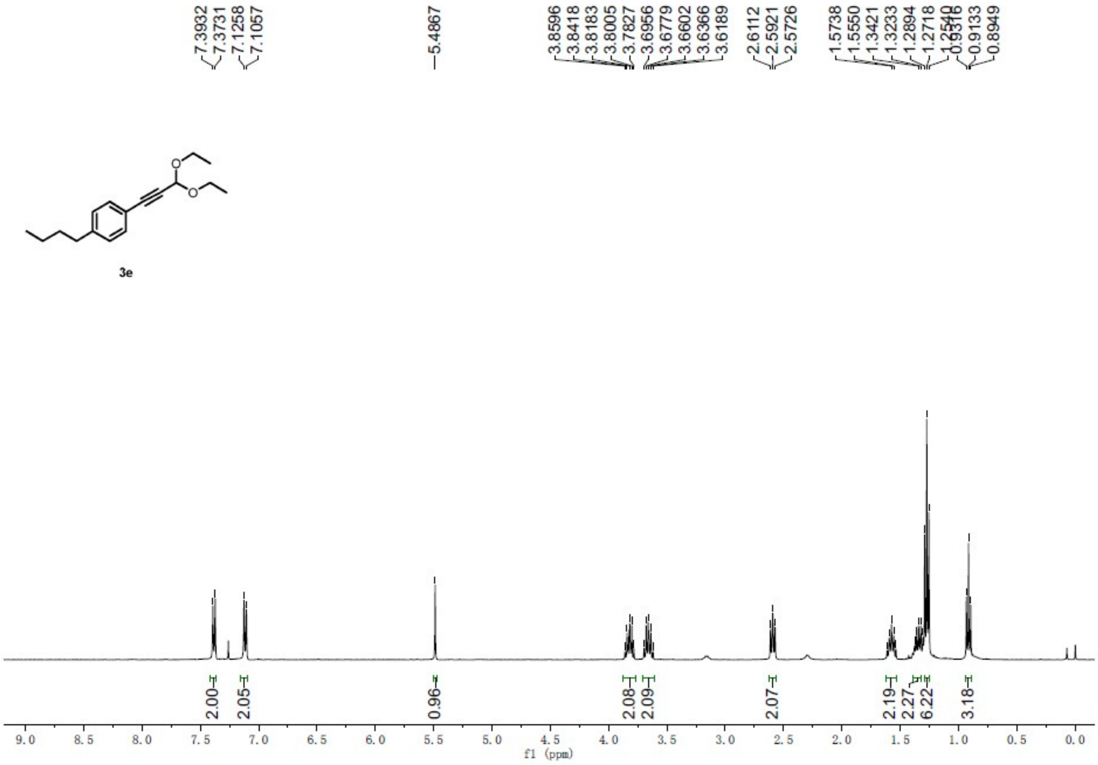
7.3932
7.3731
7.1258
7.1057
5.4867
3.8596
3.8418
3.8183
3.8005
3.7827
3.6956
3.6779
3.6602
3.6366
3.6189
2.6112
2.5921
2.5726
1.5738
1.5550
1.3421
1.3233
1.2894
1.2718
1.2540
0.9316
0.9133
0.8949
3e
2.00
2.05
0.96
2.08
2.09
2.07
2.19
2.27
6.22
3.18
f1 (ppm)

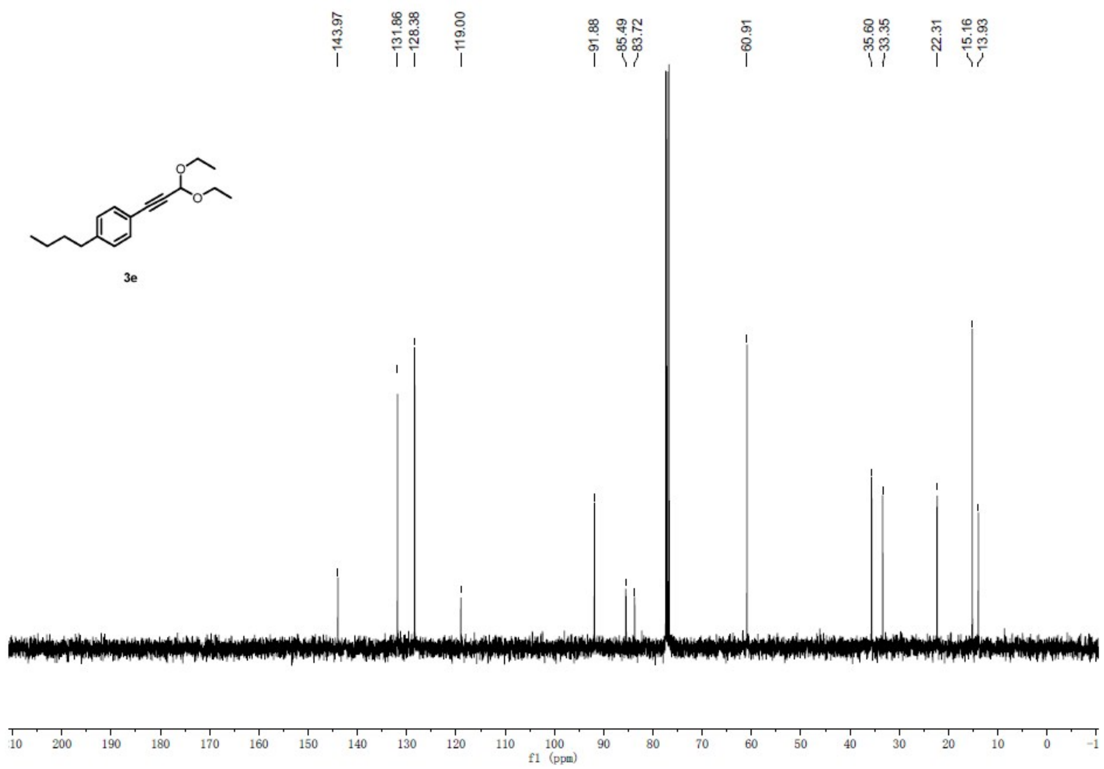
143.97
131.86
128.38
119.00
91.88
85.49
83.72
60.91
35.60
33.35
22.31
15.16
13.93
O
O
3e
f1 (ppm)

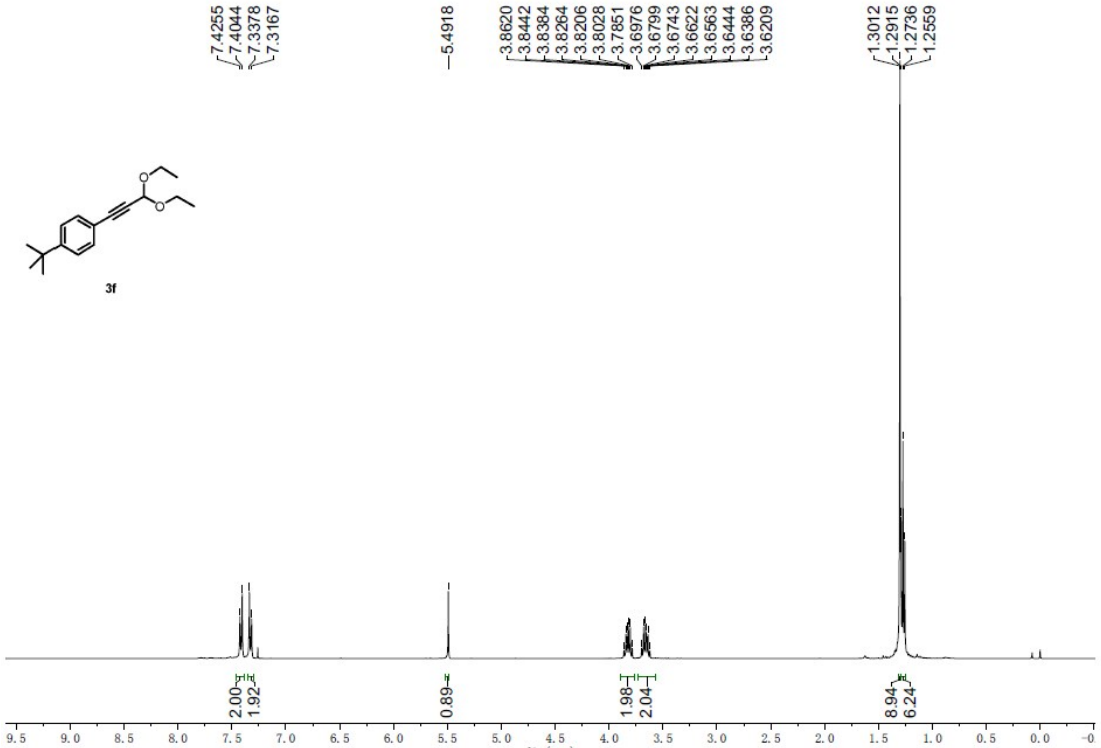
7.4255
7.4044
7.3378
7.3167
5.4918
3.8620
3.8442
3.8384
3.8264
3.8206
3.8028
3.7851
3.6976
3.6799
3.6743
3.6622
3.6563
3.6444
3.6386
3.6209
1.3012
1.2915
1.2736
1.2559
O
O
3f
2.00
1.92
0.89
1.98
2.04
8.94
6.24
f1 (ppm)

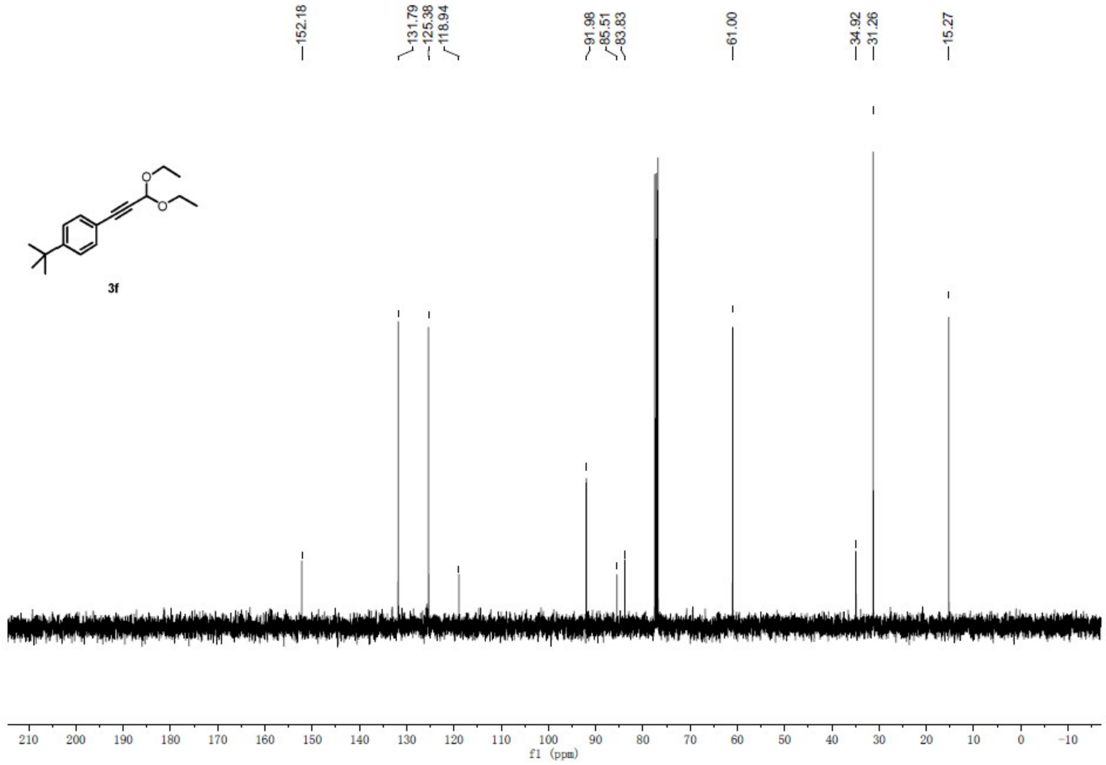
152.18
131.79
125.38
118.94
91.98
85.51
83.83
61.00
34.92
31.26
15.27
3f
210 200 190 180 170 160 150 140 130 120 110 100 90 80 70 60 50 40 30 20 10 0 -10
f1 (ppm)

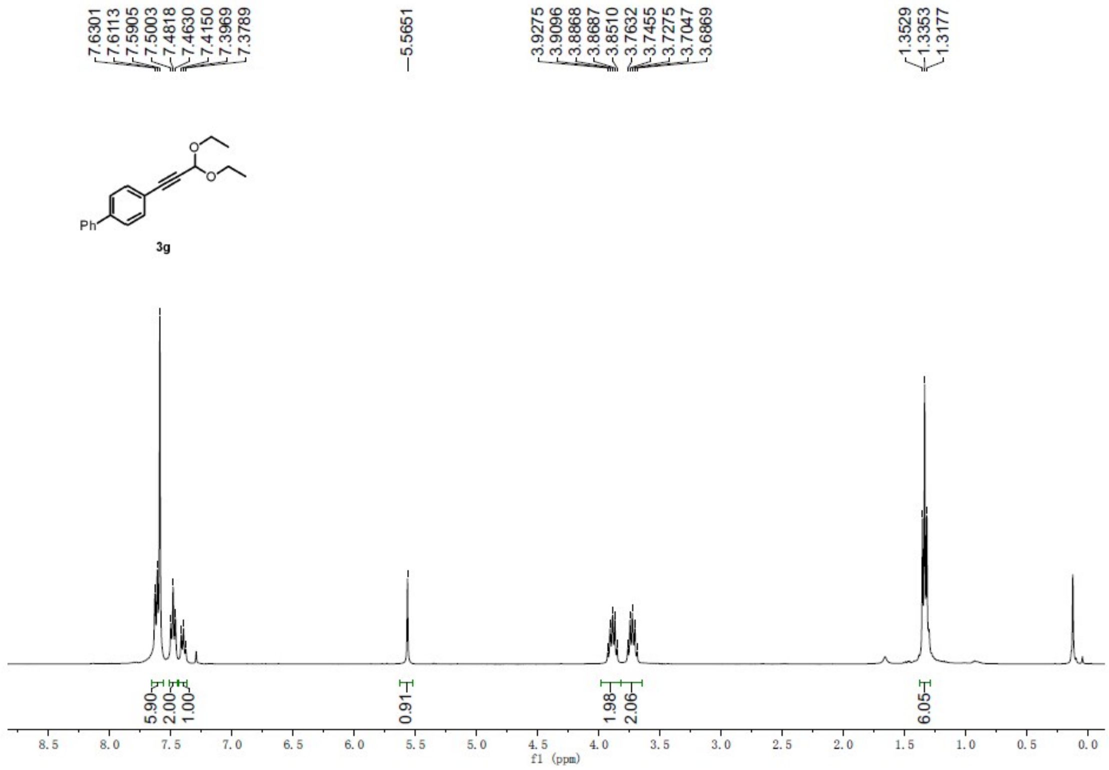
7.6301
7.6113
7.5905
7.5003
7.4818
7.4630
7.4150
7.3969
7.3789
5.5651
3.9275
3.9096
3.8868
3.8687
3.8510
3.7632
3.7455
3.7275
3.7047
3.6869
1.3529
1.3353
1.3177
Ph
3g
5.90
2.00
1.00
0.91
1.98
2.06
6.05
8.5 8.0 7.5 7.0 6.5 6.0 5.5 5.0 4.5 4.0 3.5 3.0 2.5 2.0 1.5 1.0 0.5 0.0
f1 (ppm)

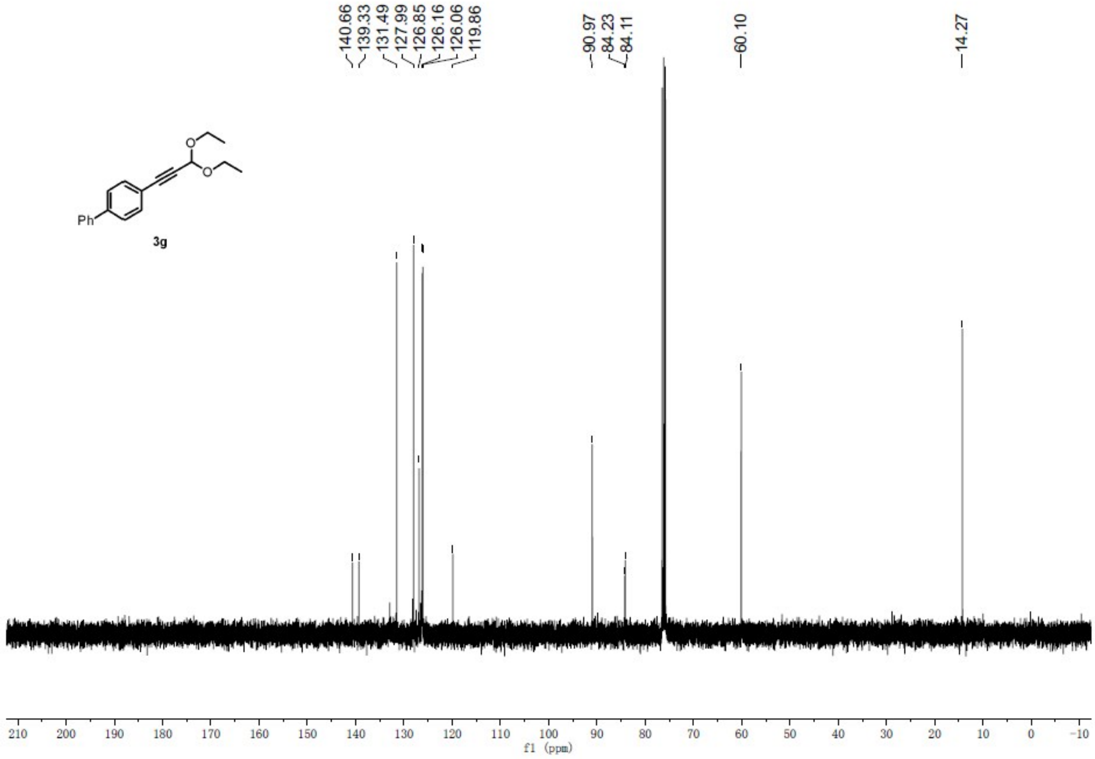
140.66
139.33
131.49
127.99
126.85
126.16
126.06
119.86
90.97
84.23
84.11
60.10
14.27
Ph
3g
210 200 190 180 170 160 150 140 130 120 110 100 90 80 70 60 50 40 30 20 10 0 -10
f1 (ppm)

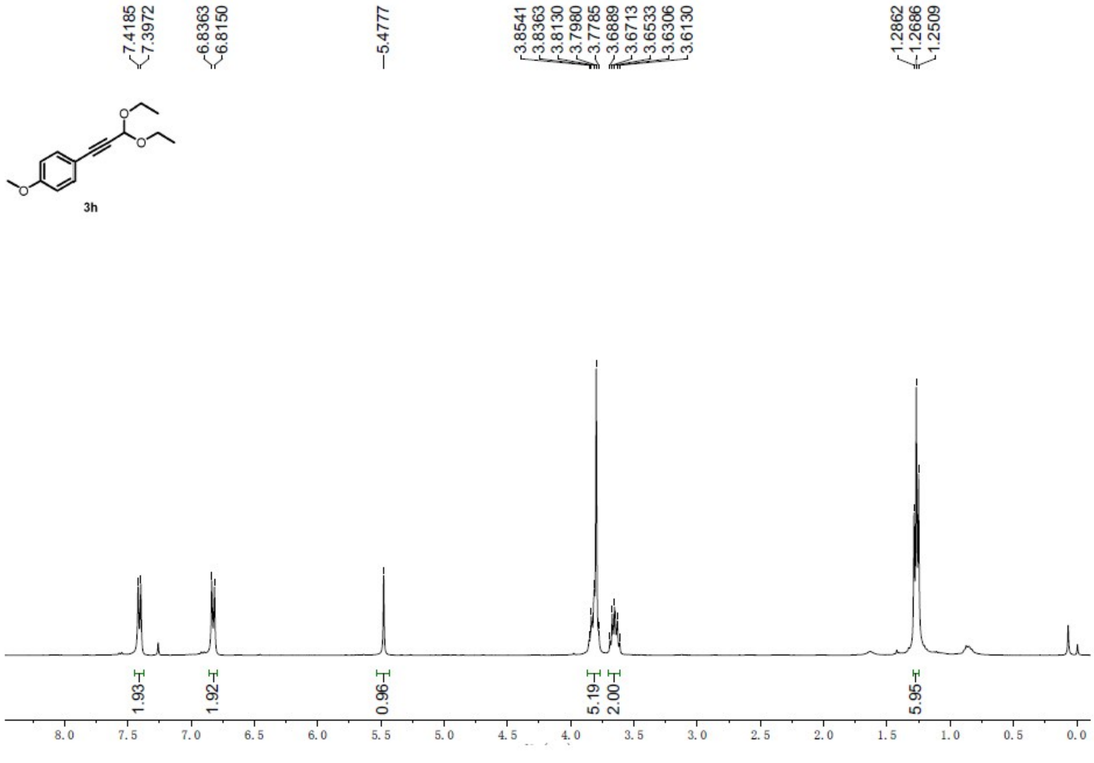
7.4185
7.3972
6.8363
6.8150
5.4777
3.8541
3.8363
3.8130
3.7980
3.7785
3.6889
3.6713
3.6533
3.6306
3.6130
1.2862
1.2686
1.2509
3h
1.93
1.92
0.96
5.19
2.00
5.95
8.0 7.5 7.0 6.5 6.0 5.5 5.0 4.5 4.0 3.5 3.0 2.5 2.0 1.5 1.0 0.5 0.0

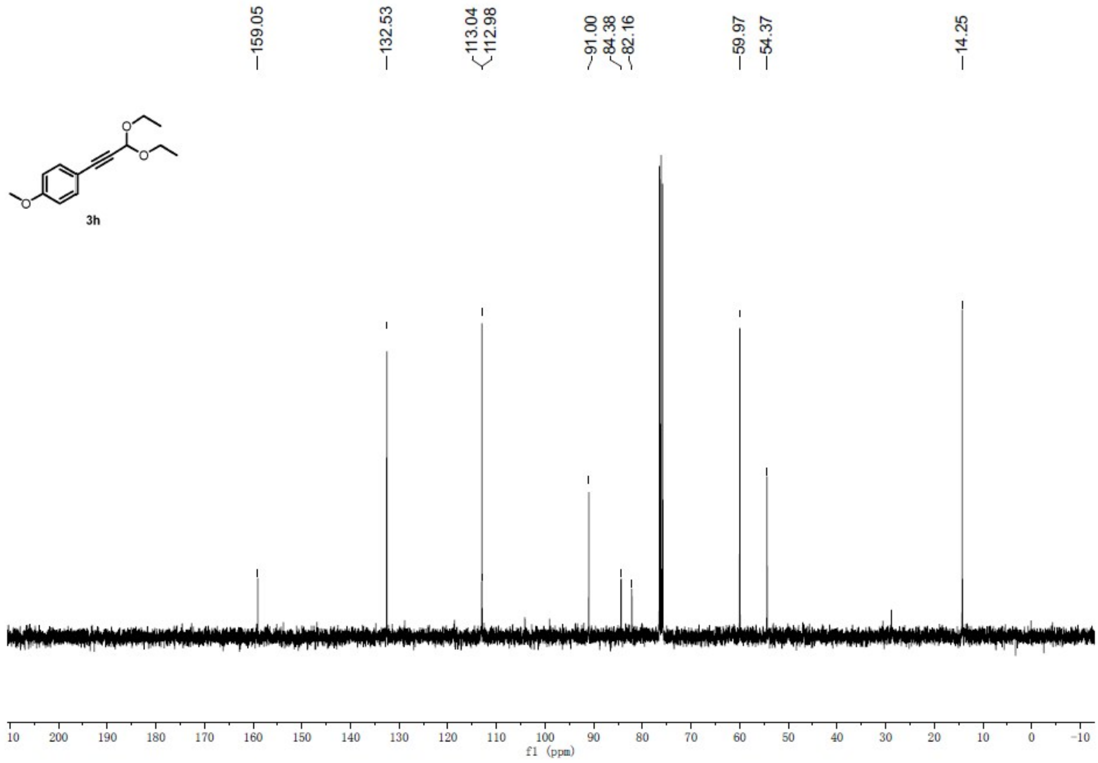
159.05
132.53
113.04
112.98
91.00
84.38
82.16
59.97
54.37
14.25
3h
f1 (ppm)

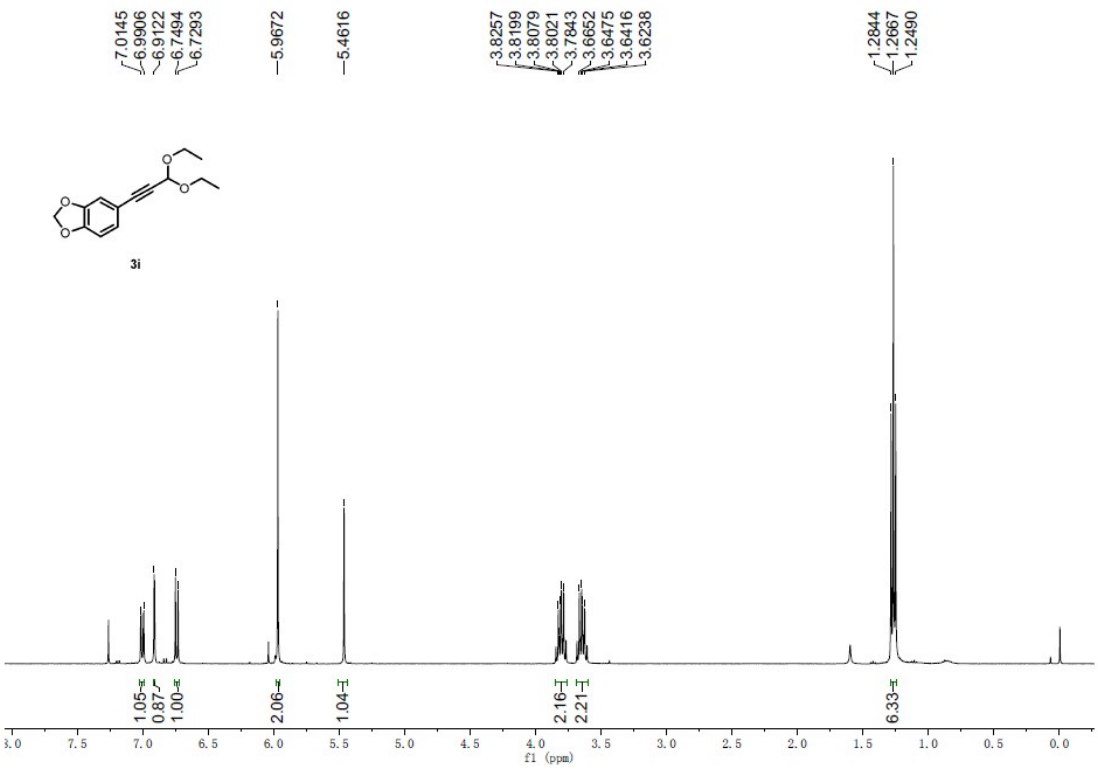
7.0145
6.9906
6.9122
6.7494
6.7293
5.9672
5.4616
3.8257
3.8199
3.8079
3.8021
3.7843
3.6652
3.6475
3.6416
3.6238
1.2844
1.2667
1.2490
3i
1.05
0.87
1.00
2.06
1.04
2.16
2.21
6.33
f1 (ppm)

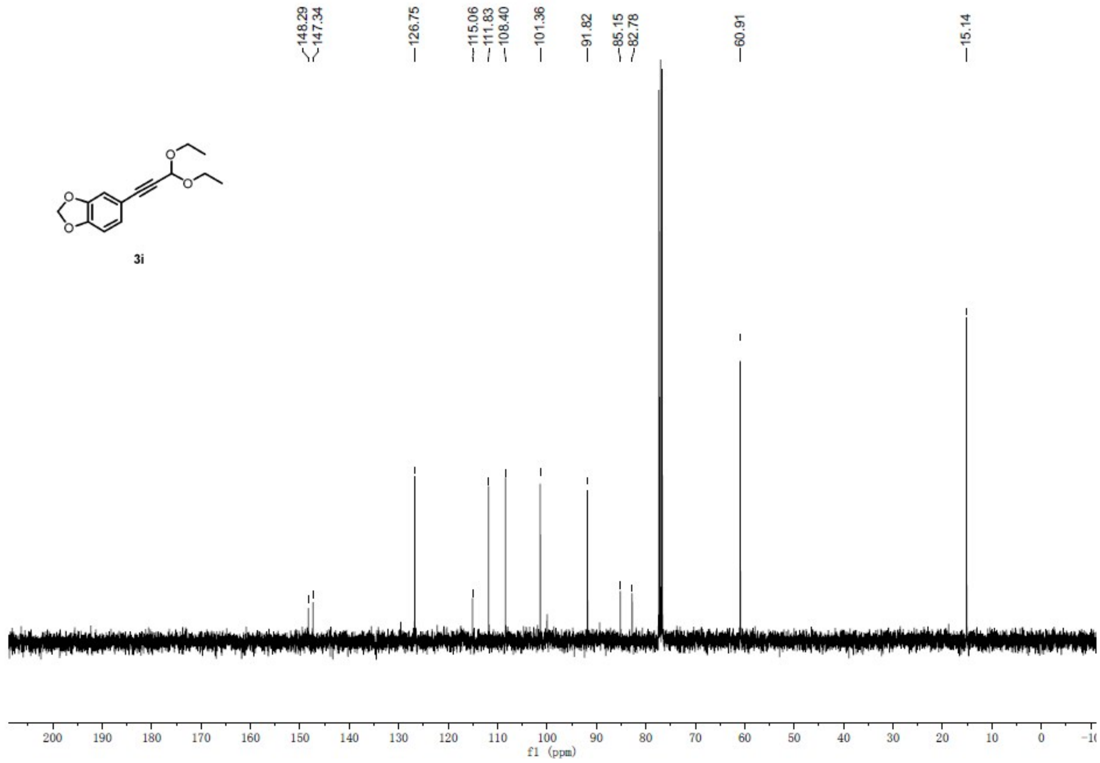
148.29
147.34
126.75
115.06
111.83
108.40
101.36
91.82
85.15
82.78
60.91
15.14
3i
f1 (ppm)

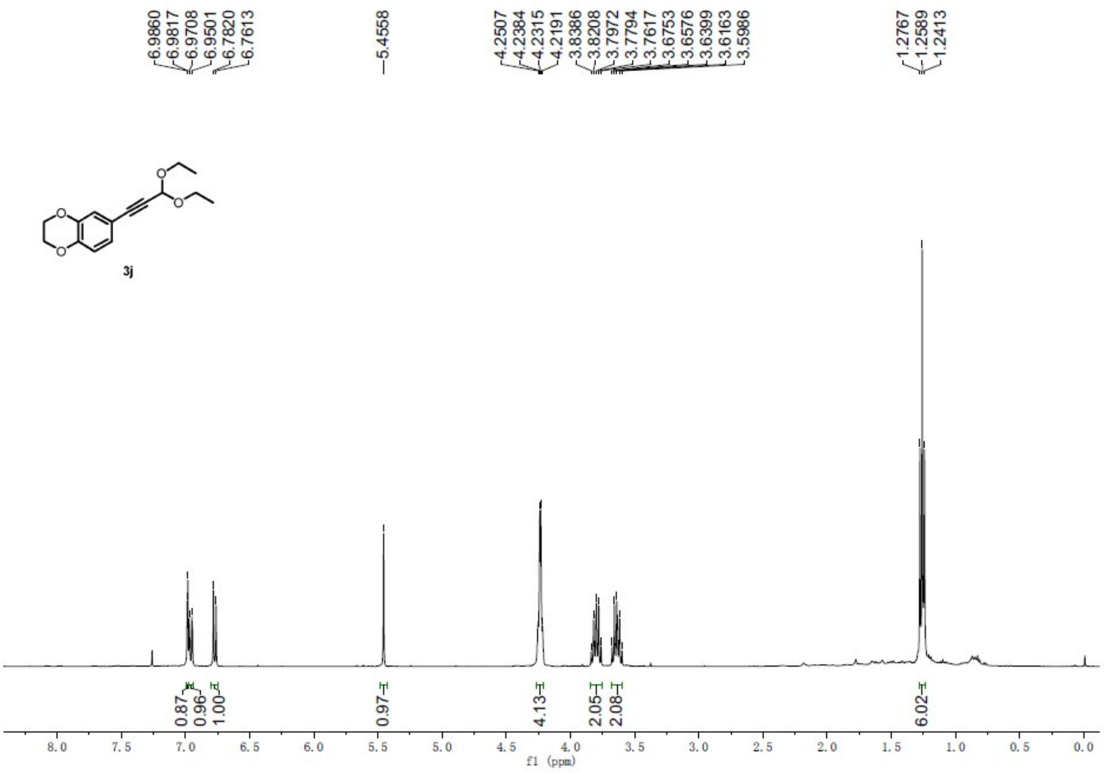

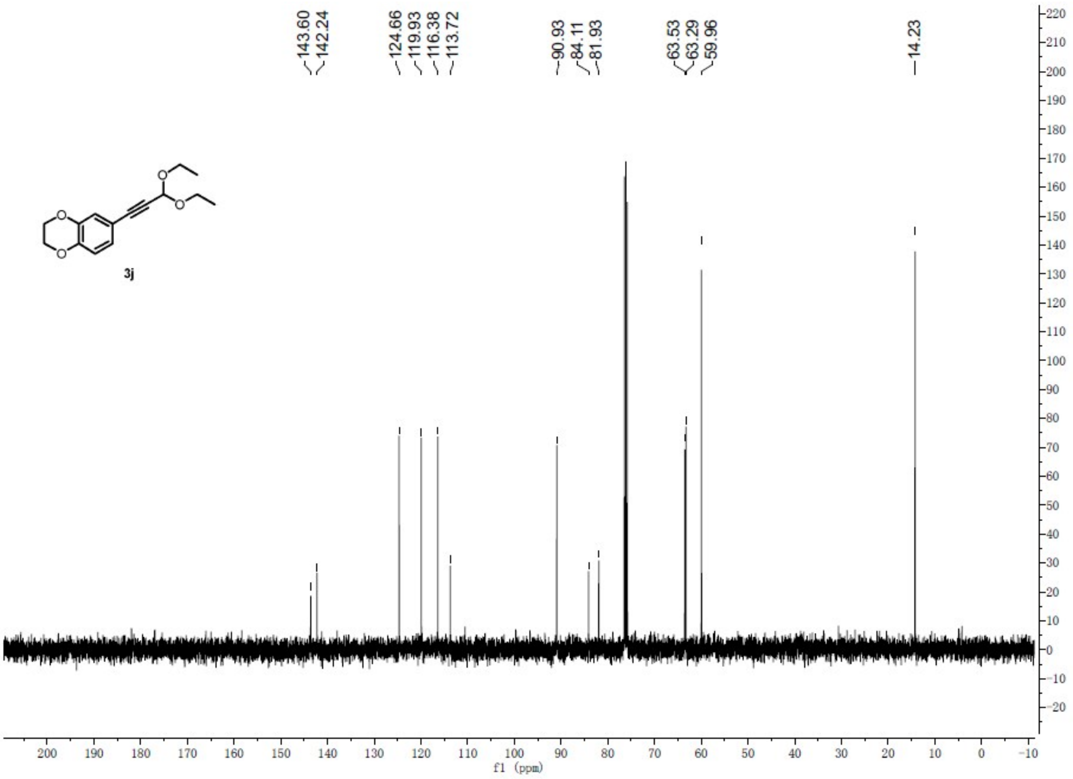

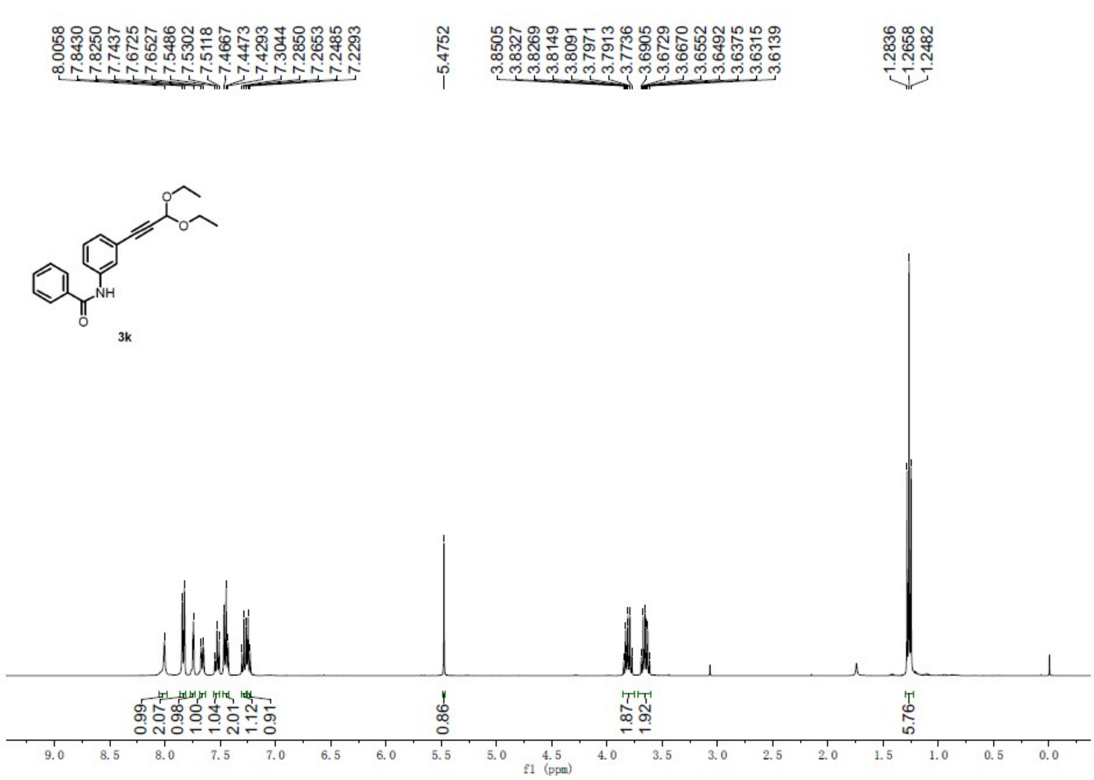
8.0058
7.8430
7.8250
7.7437
7.6725
7.6527
7.5486
7.5302
7.5118
7.4667
7.4473
7.4293
7.3044
7.2850
7.2653
7.2485
7.2293
5.4752
3.8505
3.8327
3.8269
3.8149
3.8091
3.7971
3.7913
3.7736
3.6905
3.6729
3.6670
3.6552
3.6492
3.6375
3.6315
3.6139
1.2836
1.2658
1.2482
O
O
NH
O
3k
0.99
2.07
0.98
1.00
1.04
2.01
1.12
0.91
0.86
1.87
1.92
5.76
9.0
8.5
8.0
7.5
7.0
6.5
6.0
5.5
5.0
4.5
4.0
3.5
3.0
2.5
2.0
1.5
1.0
0.5
0.0
f1 (ppm)

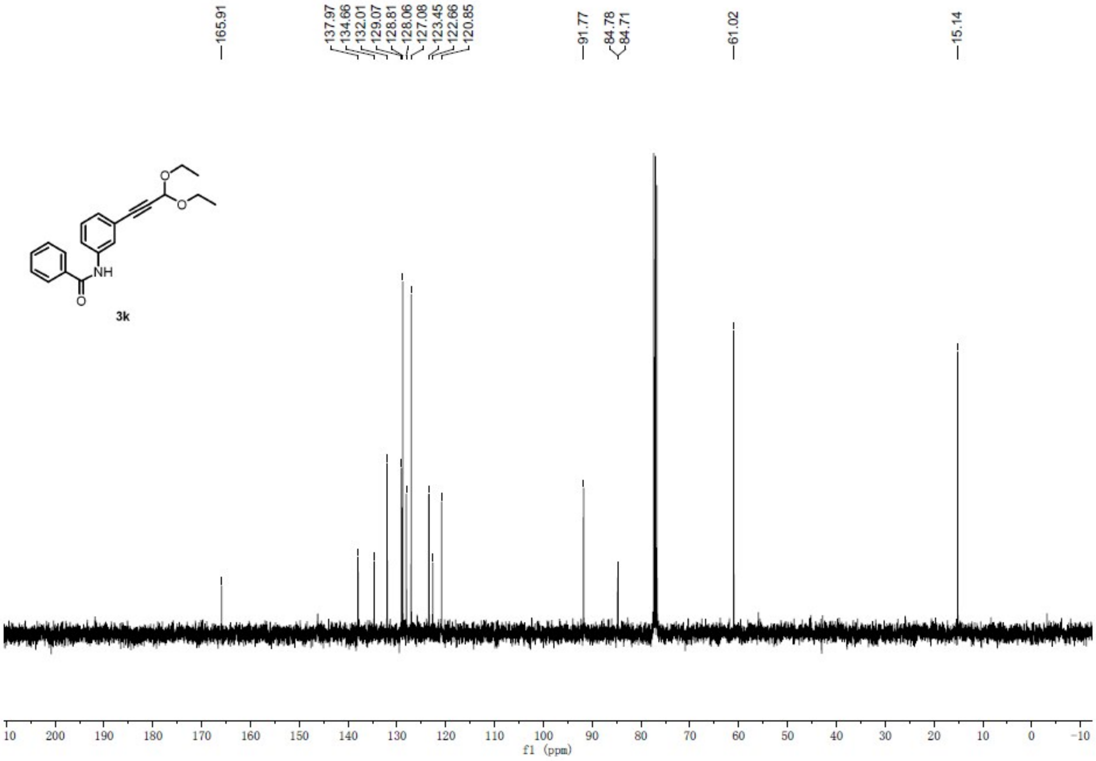
165.91
137.97
134.66
132.01
129.07
128.81
128.06
127.08
123.45
122.66
120.85
91.77
84.78
84.71
61.02
15.14
NH
O
3k
210 200 190 180 170 160 150 140 130 120 110 100 90 80 70 60 50 40 30 20 10 0 -10
f1 (ppm)

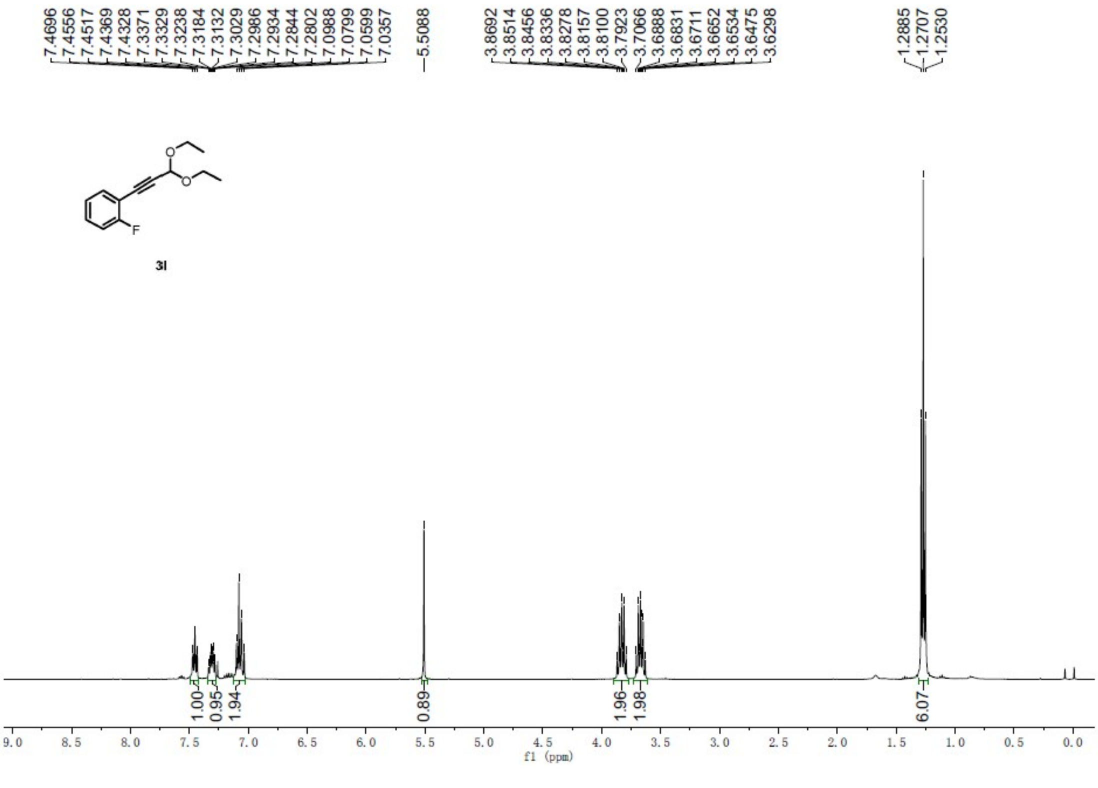
7.4696
7.4556
7.4517
7.4369
7.4328
7.3371
7.3329
7.3238
7.3184
7.3132
7.3029
7.2986
7.2934
7.2844
7.2802
7.0988
7.0799
7.0599
7.0357
5.5088
3.8692
3.8514
3.8456
3.8336
3.8278
3.8157
3.8100
3.7923
3.7066
3.6888
3.6831
3.6711
3.6652
3.6534
3.6475
3.6298
1.2885
1.2707
1.2530
O
F
3l
1.00
0.95
1.94
0.89
1.96
1.98
6.07
9.0 8.5 8.0 7.5 7.0 6.5 6.0 5.5 5.0 4.5 4.0 3.5 3.0 2.5 2.0 1.5 1.0 0.5 0.0
f1 (ppm)

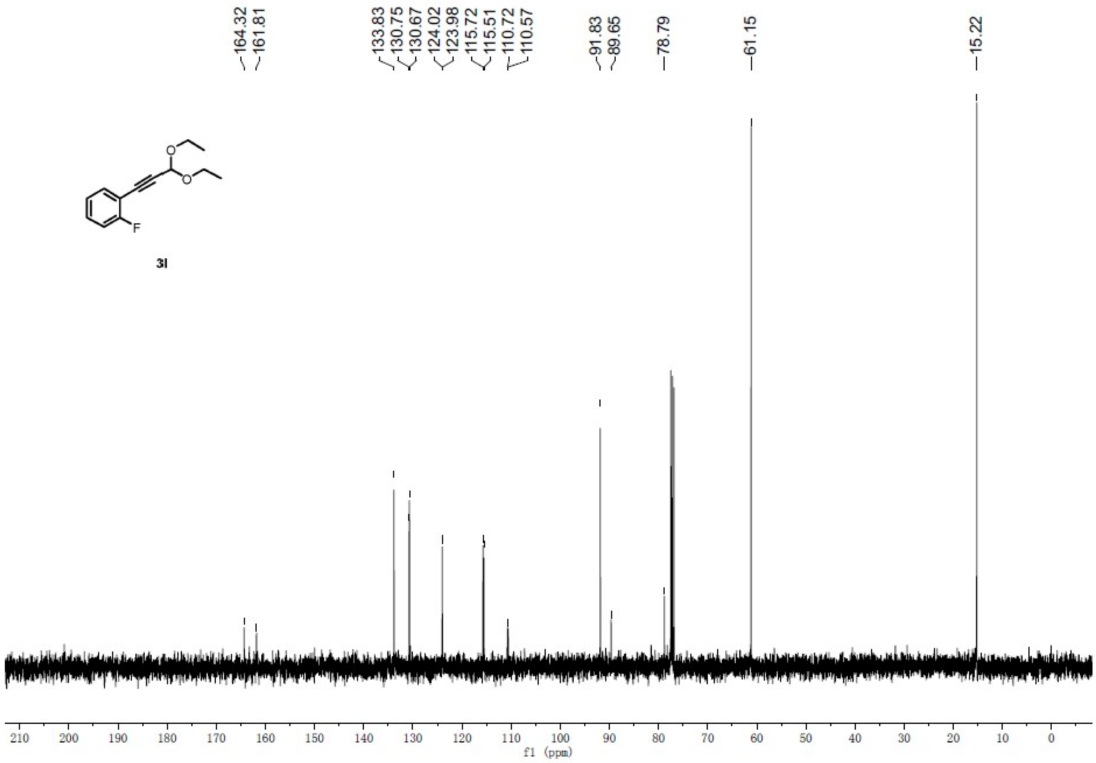
164.32
161.81
133.83
130.75
130.67
124.02
123.98
115.72
115.51
110.72
110.57
91.83
89.65
78.79
61.15
15.22
F
O
O
3l
210
200
190
180
170
160
150
140
130
120
110
100
90
80
70
60
50
40
30
20
10
0
f1 (ppm)

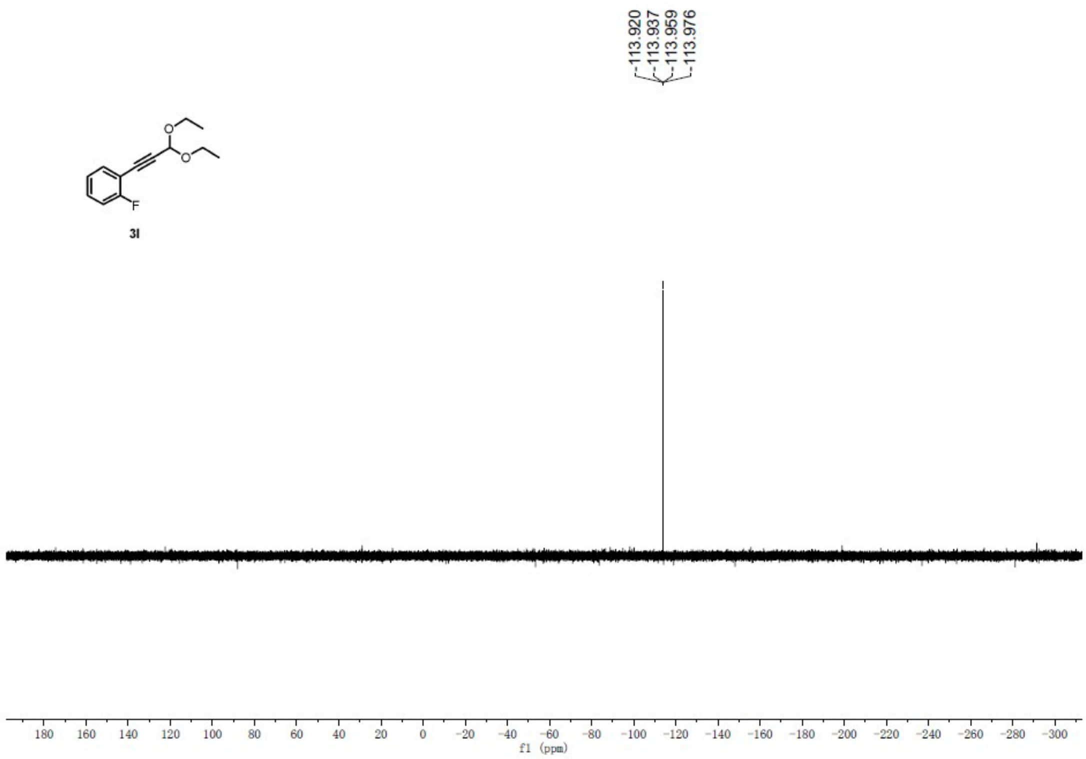

113.920
113.937
113.959
113.976
3l
f1 (ppm)

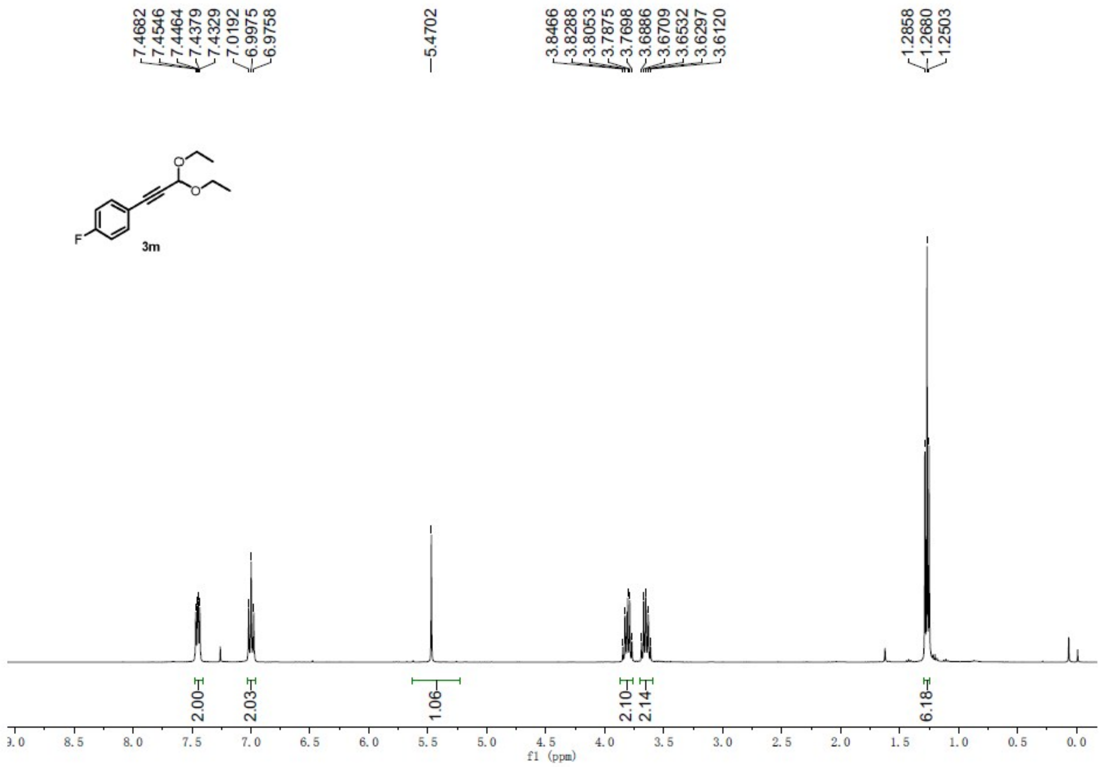

7.4682
7.4546
7.4464
7.4379
7.4329
7.0192
6.9975
6.9758
5.4702
3.8466
3.8288
3.8053
3.7875
3.7698
3.6886
3.6709
3.6532
3.6297
3.6120
1.2858
1.2680
1.2503
3m
2.00
2.03
1.06
2.10
2.14
6.18
f1 (ppm)

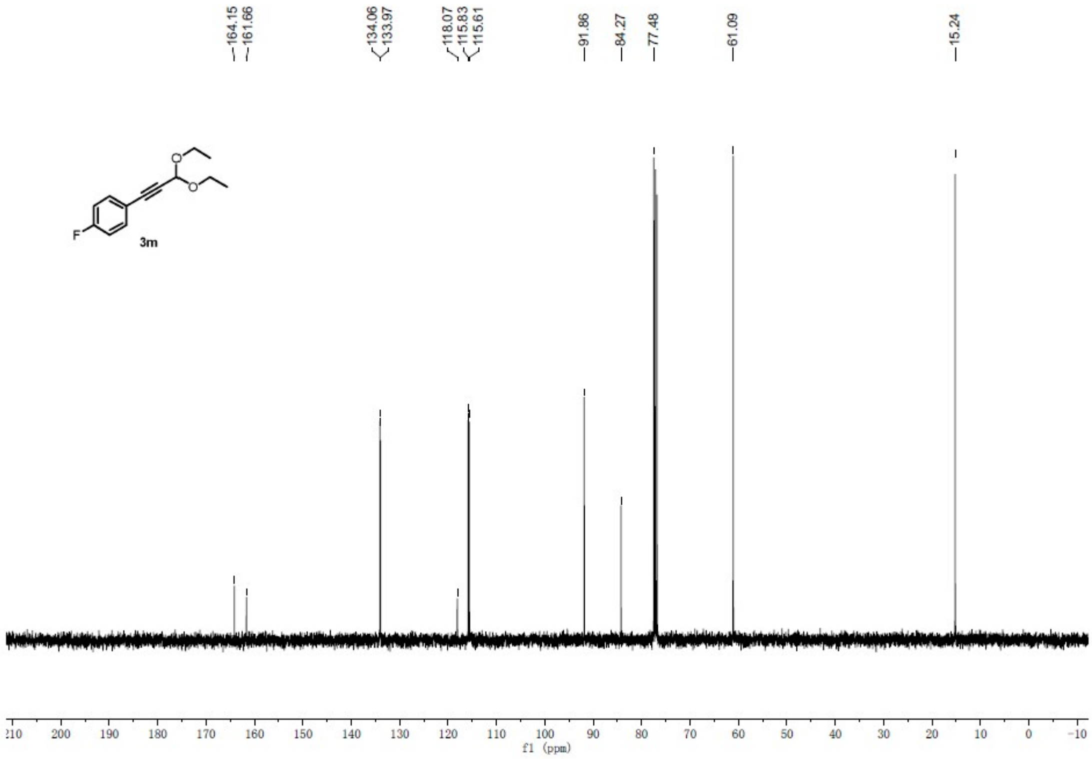
164.15
161.66
134.06
133.97
118.07
115.83
115.61
91.86
84.27
77.48
61.09
15.24
F
O
O
3m
210 200 190 180 170 160 150 140 130 120 110 100 90 80 70 60 50 40 30 20 10 0 -10
f1 (ppm)

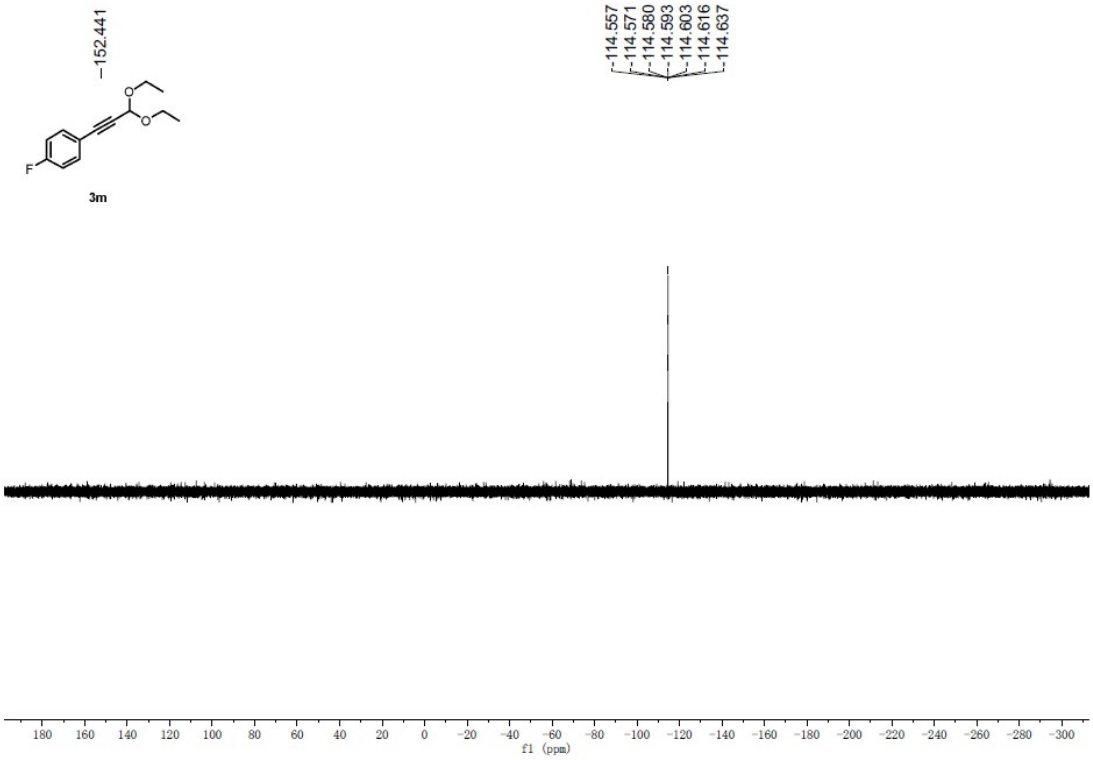

152.441
114.557
114.571
114.580
114.593
114.603
114.616
114.637
F
3m
180 160 140 120 100 80 60 40 20 0 −20 −40 −60 −80 −100 −120 −140 −160 −180 −200 −220 −240 −260 −280 −300
f1 (ppm)

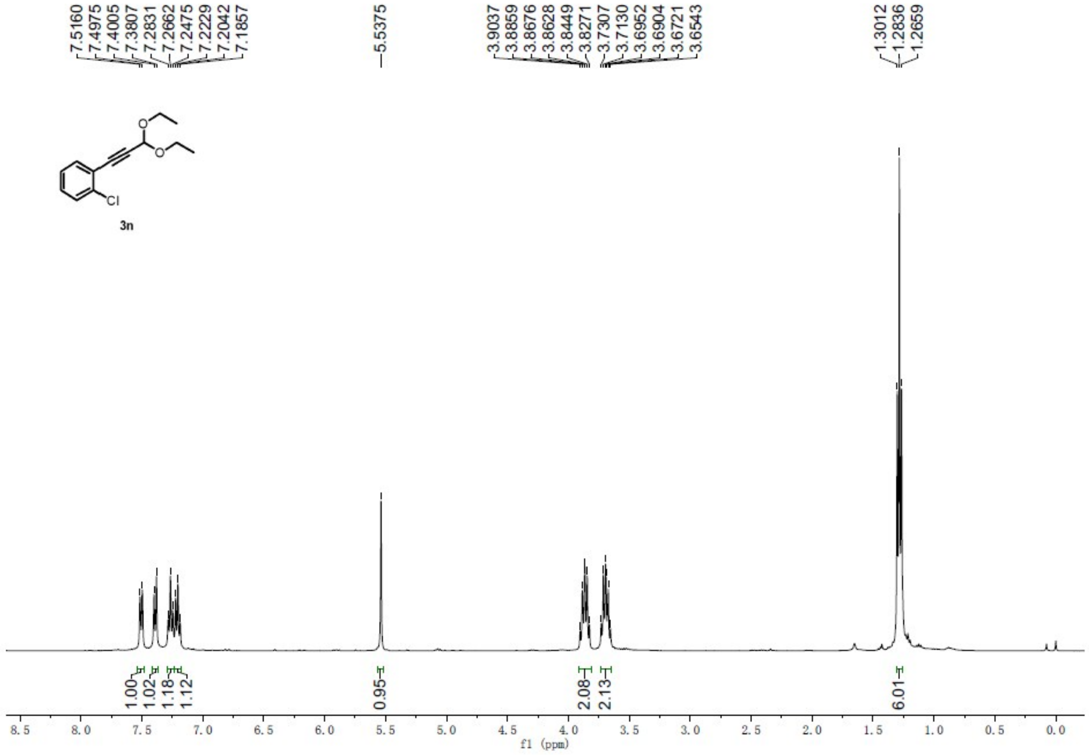

7.5160
7.4975
7.4005
7.3807
7.2831
7.2662
7.2475
7.2229
7.2042
7.1857
5.5375
3.9037
3.8859
3.8676
3.8628
3.8449
3.8271
3.7307
3.7130
3.6952
3.6904
3.6721
3.6543
1.3012
1.2836
1.2659
Cl
3n
1.00
1.02
1.18
1.12
0.95
2.08
2.13
6.01
8.5 8.0 7.5 7.0 6.5 6.0 5.5 5.0 4.5 4.0 3.5 3.0 2.5 2.0 1.5 1.0 0.5 0.0
f1 (ppm)

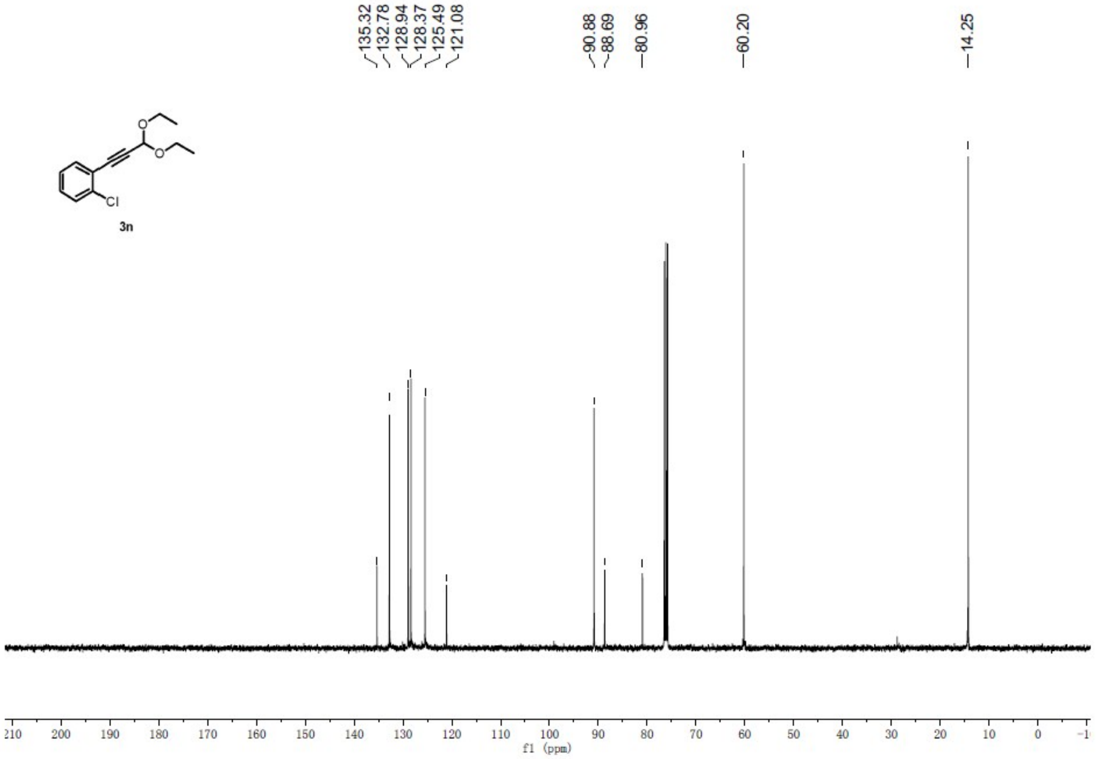
135.32
132.78
128.94
128.37
125.49
121.08
90.88
88.69
80.96
60.20
14.25
O
O
Cl
3n
210 200 190 180 170 160 150 140 130 120 110 100 90 80 70 60 50 40 30 20 10 0
f1 (ppm)

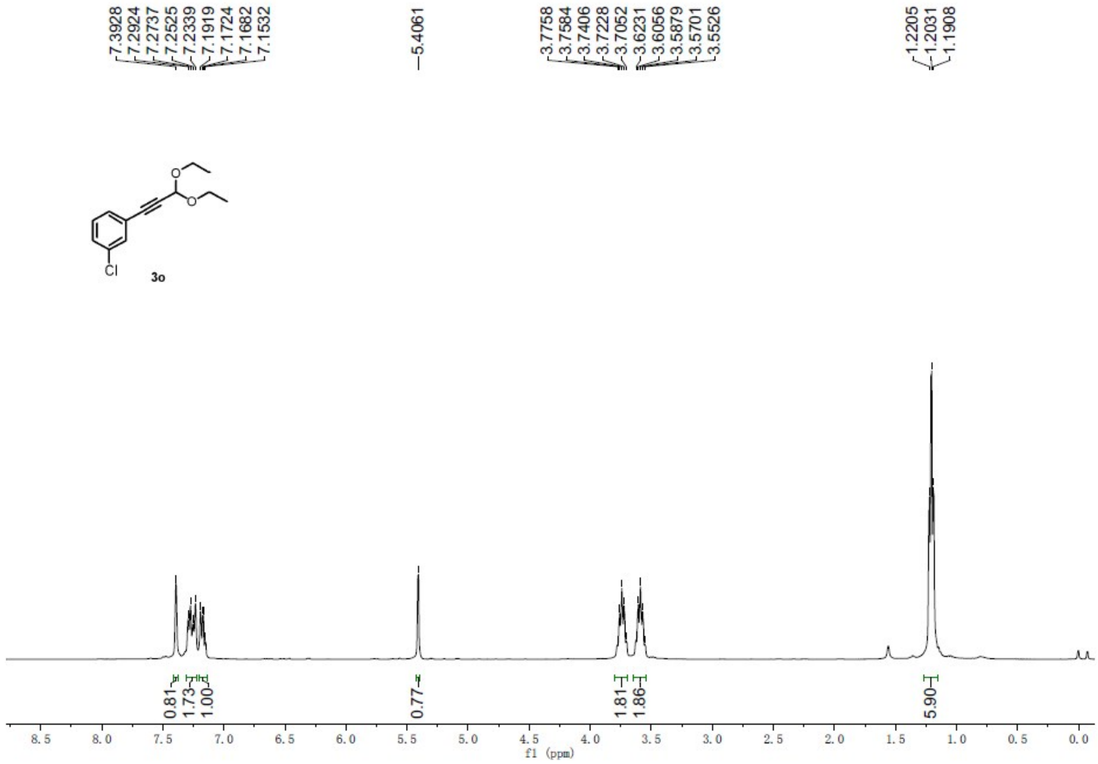
7.3928
7.2924
7.2737
7.2525
7.2339
7.1919
7.1724
7.1682
7.1532
5.4061
3.7758
3.7584
3.7406
3.7228
3.7052
3.6231
3.6056
3.5879
3.5701
3.5526
1.2205
1.2031
1.1908
O
O
Cl
3o
0.81
1.73
1.00
0.77
1.81
1.86
5.90
8.5 8.0 7.5 7.0 6.5 6.0 5.5 5.0 4.5 4.0 3.5 3.0 2.5 2.0 1.5 1.0 0.5 0.0
f1 (ppm)

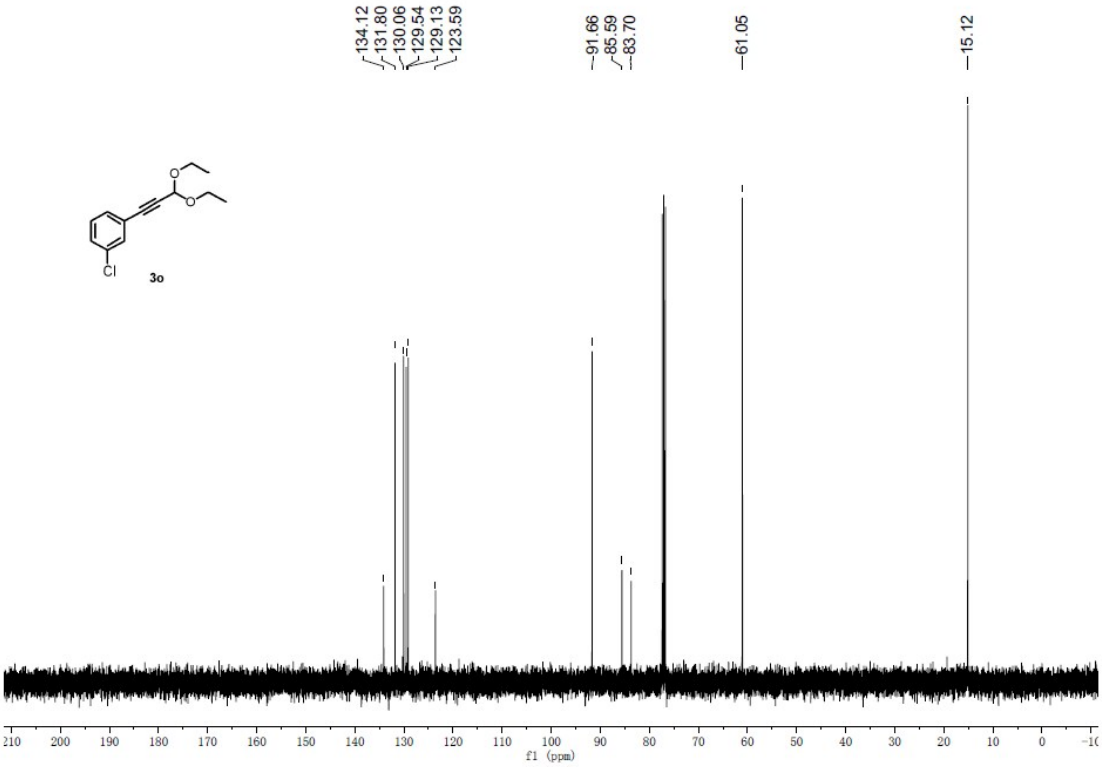
134.12
131.80
130.06
129.54
129.13
123.59
91.66
85.59
83.70
61.05
15.12
Cl
3o
210 200 190 180 170 160 150 140 130 120 110 100 90 80 70 60 50 40 30 20 10 0 -10
f1 (ppm)

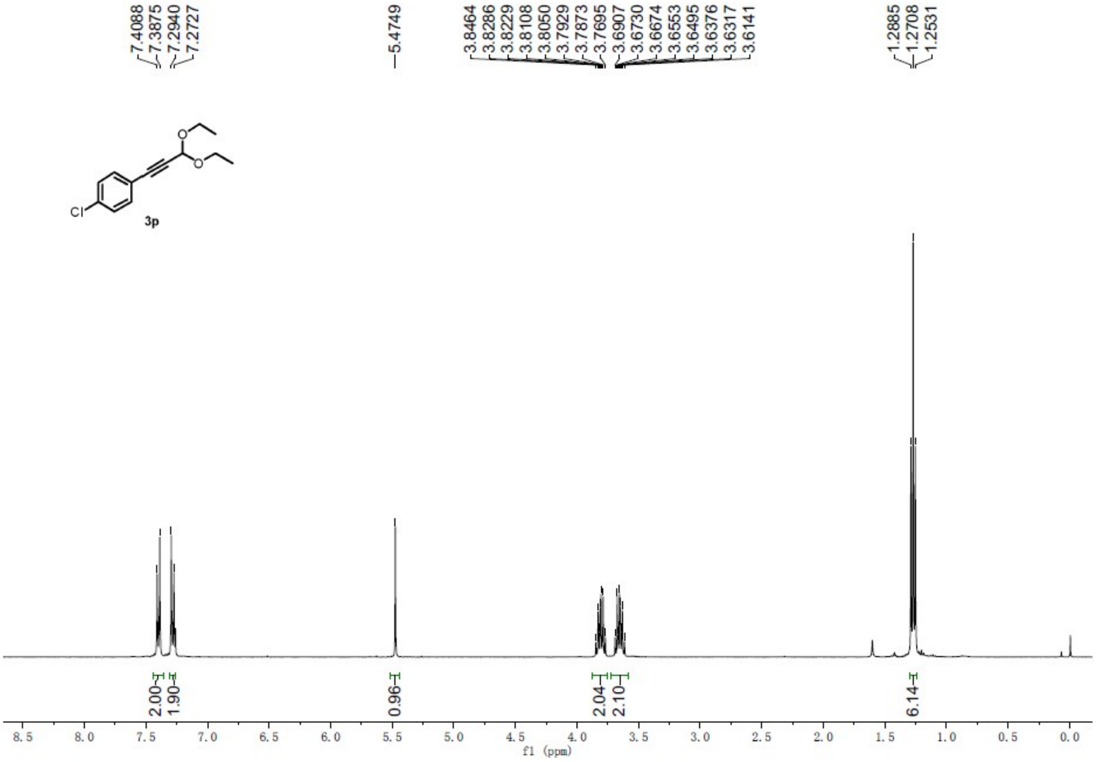
7.4088
7.3875
7.2940
7.2727
5.4749
3.8464
3.8286
3.8229
3.8108
3.8050
3.7929
3.7873
3.7695
3.6907
3.6730
3.6674
3.6553
3.6495
3.6376
3.6317
3.6141
1.2885
1.2708
1.2531
Cl
3p
2.00
1.90
0.96
2.04
2.10
6.14
8.5 8.0 7.5 7.0 6.5 6.0 5.5 5.0 4.5 4.0 3.5 3.0 2.5 2.0 1.5 1.0 0.5 0.0
f1 (ppm)

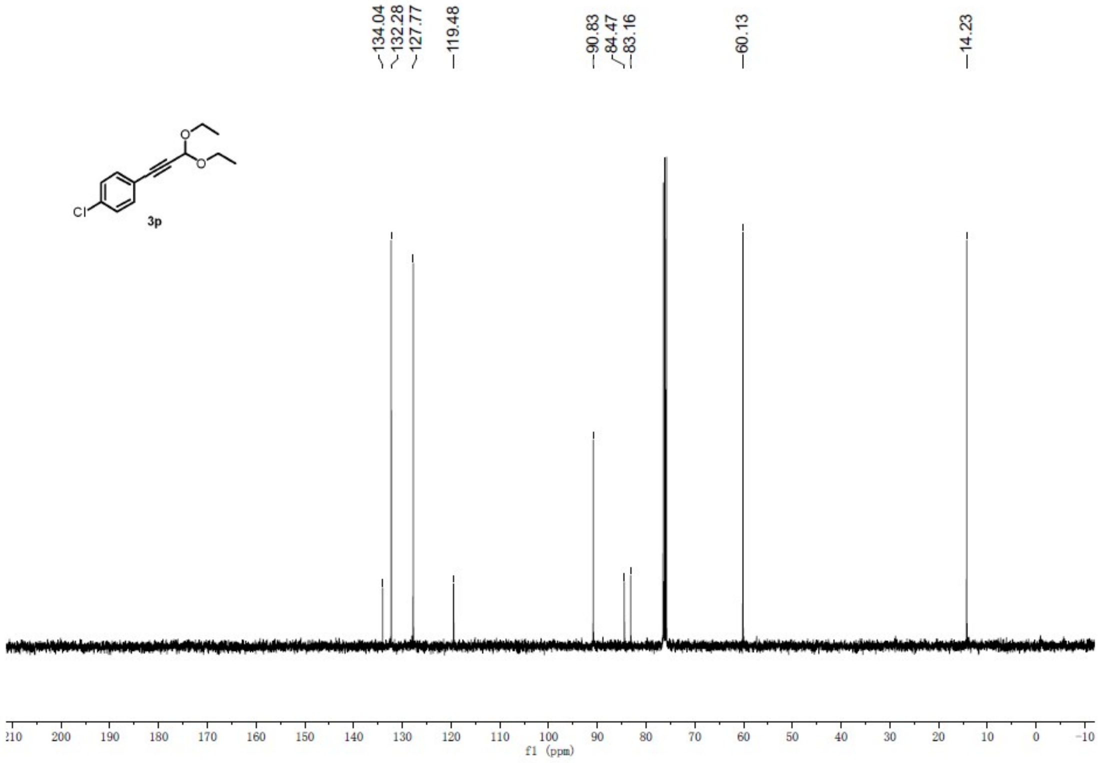
134.04
132.28
127.77
119.48
90.83
84.47
83.16
60.13
14.23
Cl
O
O
3p
210 200 190 180 170 160 150 140 130 120 110 100 90 80 70 60 50 40 30 20 10 0 -10
f1 (ppm)

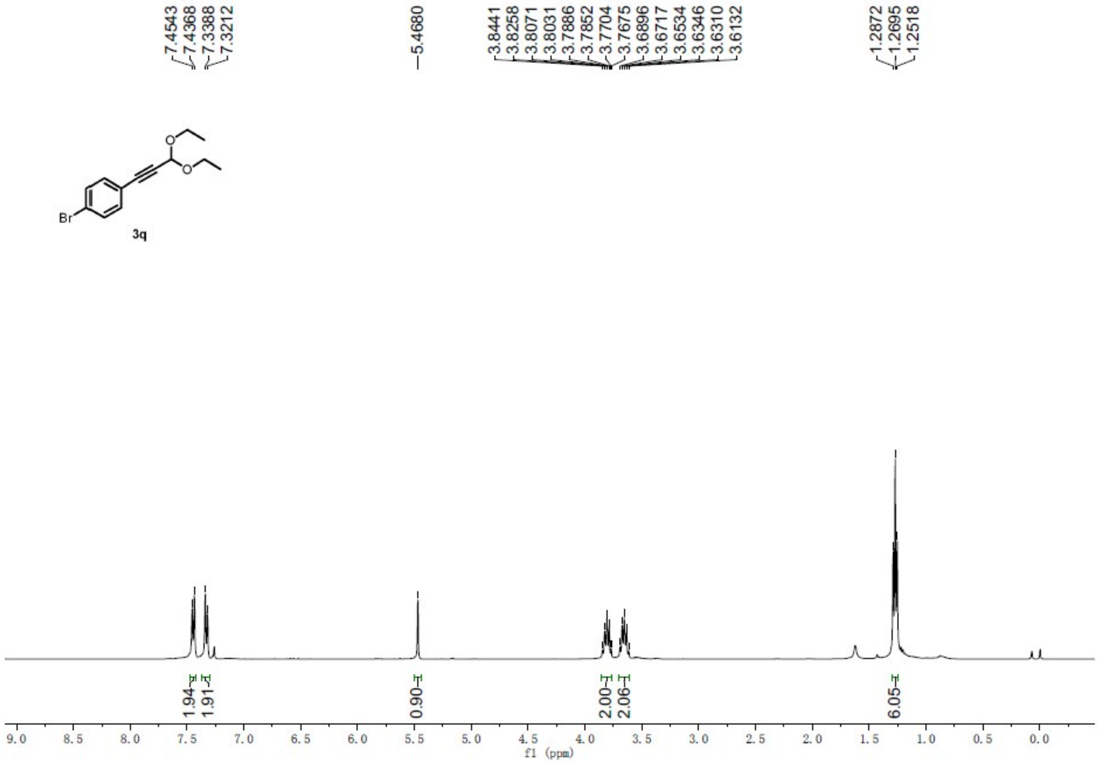
7.4543
7.4368
7.3388
7.3212
5.4680
3.8441
3.8258
3.8071
3.8031
3.7886
3.7852
3.7704
3.7675
3.6896
3.6717
3.6534
3.6346
3.6310
3.6132
1.2872
1.2695
1.2518
Br
O
O
3q
1.94
1.91
0.90
2.00
2.06
6.05
9.0 8.5 8.0 7.5 7.0 6.5 6.0 5.5 5.0 4.5 4.0 3.5 3.0 2.5 2.0 1.5 1.0 0.5 0.0
f1 (ppm)

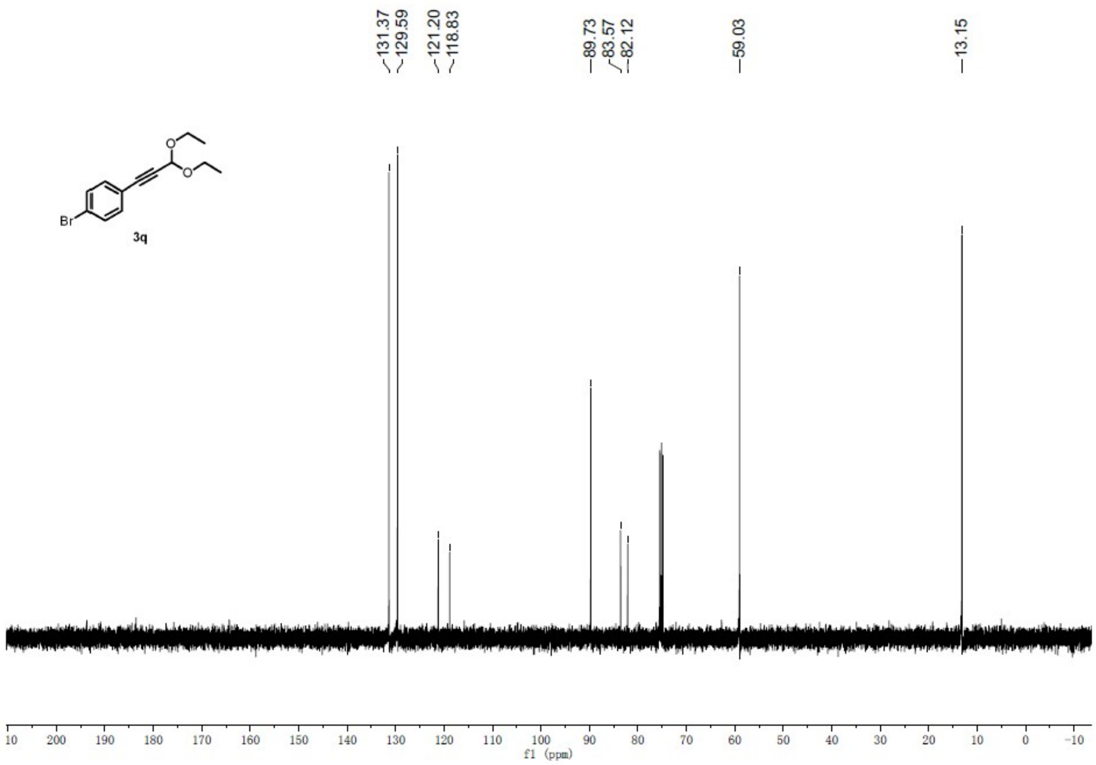
131.37
129.59
121.20
118.83
89.73
83.57
82.12
59.03
13.15
Br
3q
f1 (ppm)

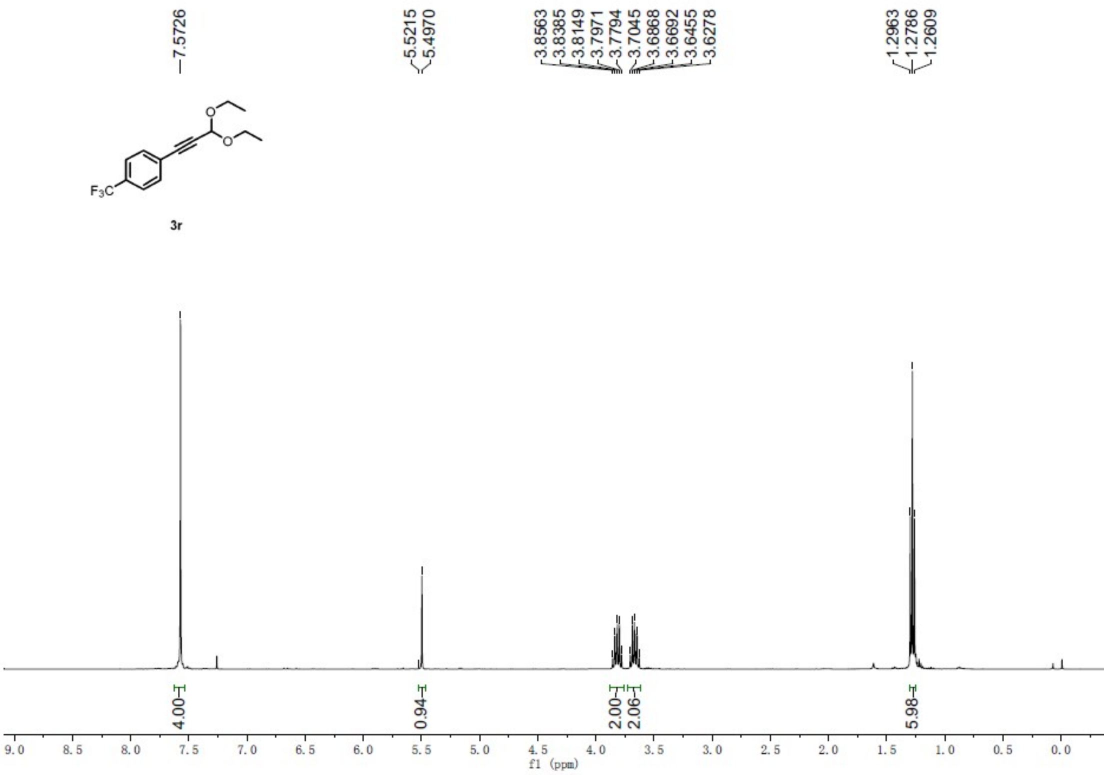
7.5726
5.5215
5.4970
3.8563
3.8385
3.8149
3.7971
3.7794
3.7045
3.6868
3.6692
3.6455
3.6278
1.2963
1.2786
1.2609
F3C
3r
4.00
0.94
2.00
2.06
5.98
f1 (ppm)

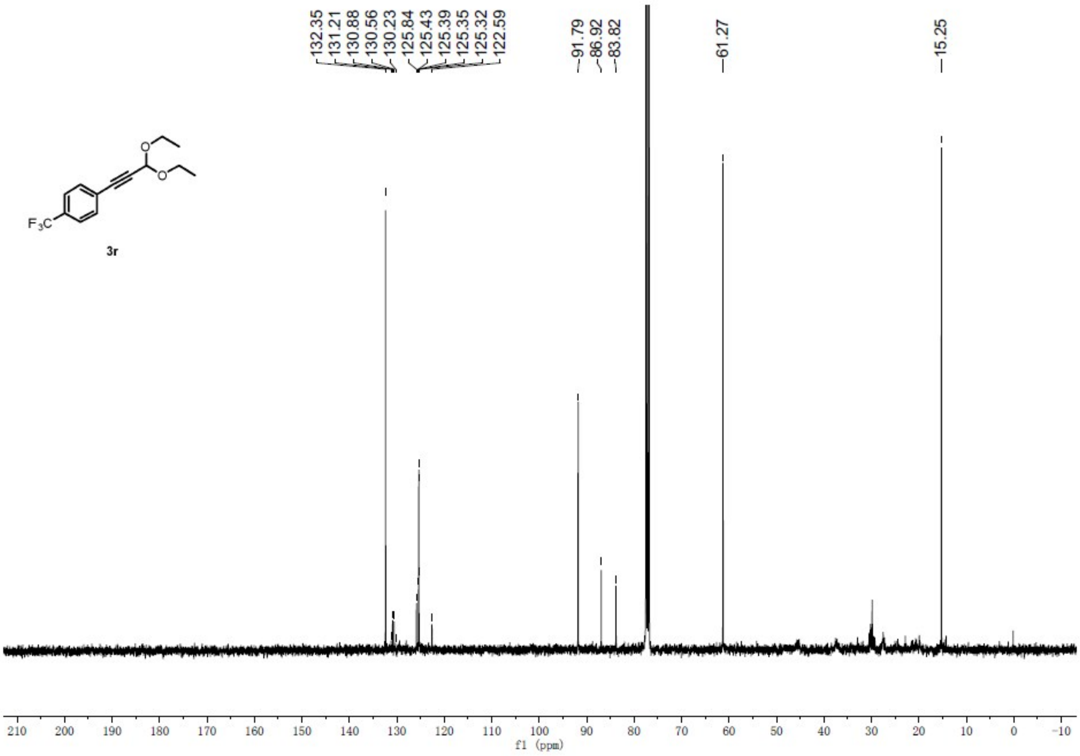
132.35
131.21
130.88
130.56
130.23
125.84
125.43
125.39
125.35
125.32
122.59
91.79
86.92
83.82
61.27
15.25
F3C
3r
f1 (ppm)

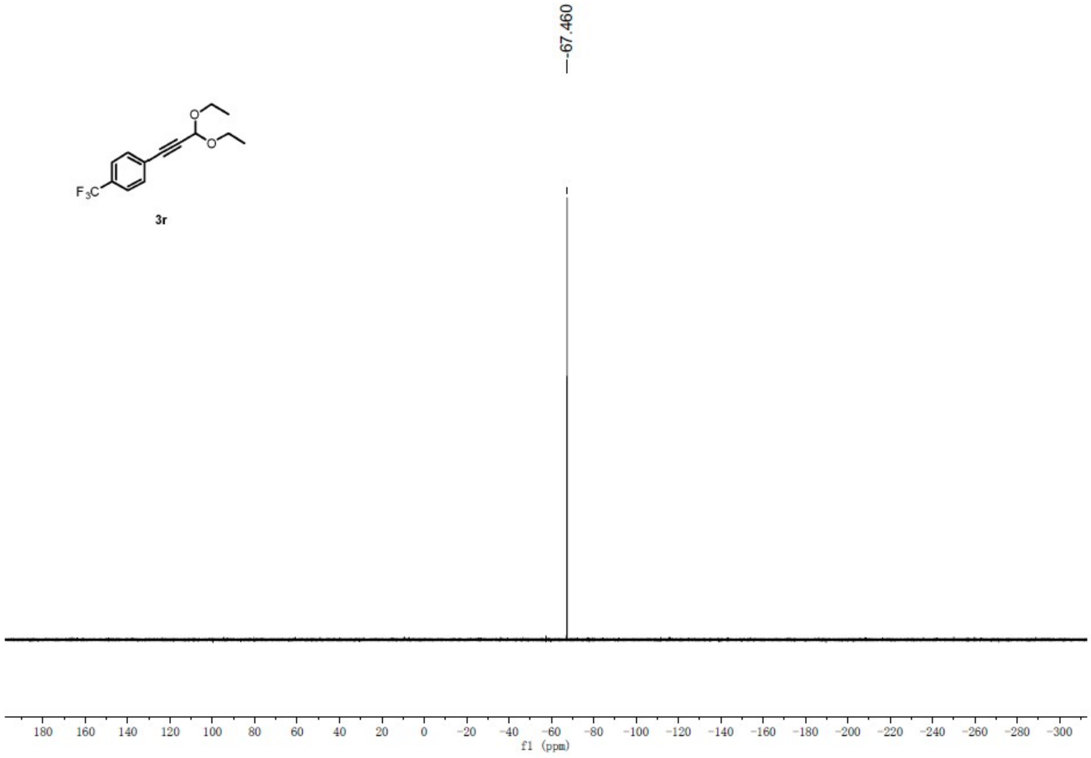
-67.460
F_3C
3r
180 160 140 120 100 80 60 40 20 0 -20 -40 -60 -80 -100 -120 -140 -160 -180 -200 -220 -240 -260 -280 -300
f1 (ppm)

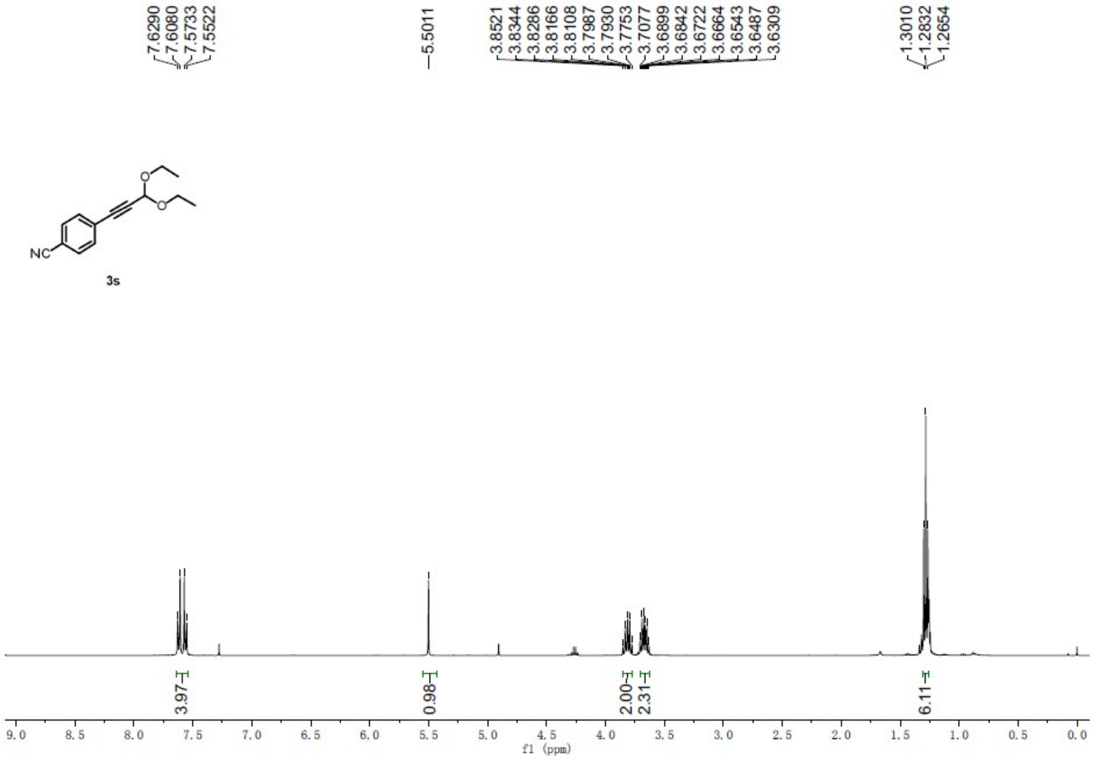
7.6290
7.6080
7.5733
7.5522
5.5011
3.8521
3.8344
3.8286
3.8166
3.8108
3.7987
3.7930
3.7753
3.7077
3.6899
3.6842
3.6722
3.6664
3.6543
3.6487
3.6309
1.3010
1.2832
1.2654
NC
3s
3.97
0.98
2.00
2.31
6.11
9.0 8.5 8.0 7.5 7.0 6.5 6.0 5.5 5.0 4.5 4.0 3.5 3.0 2.5 2.0 1.5 1.0 0.5 0.0
f1 (ppm)

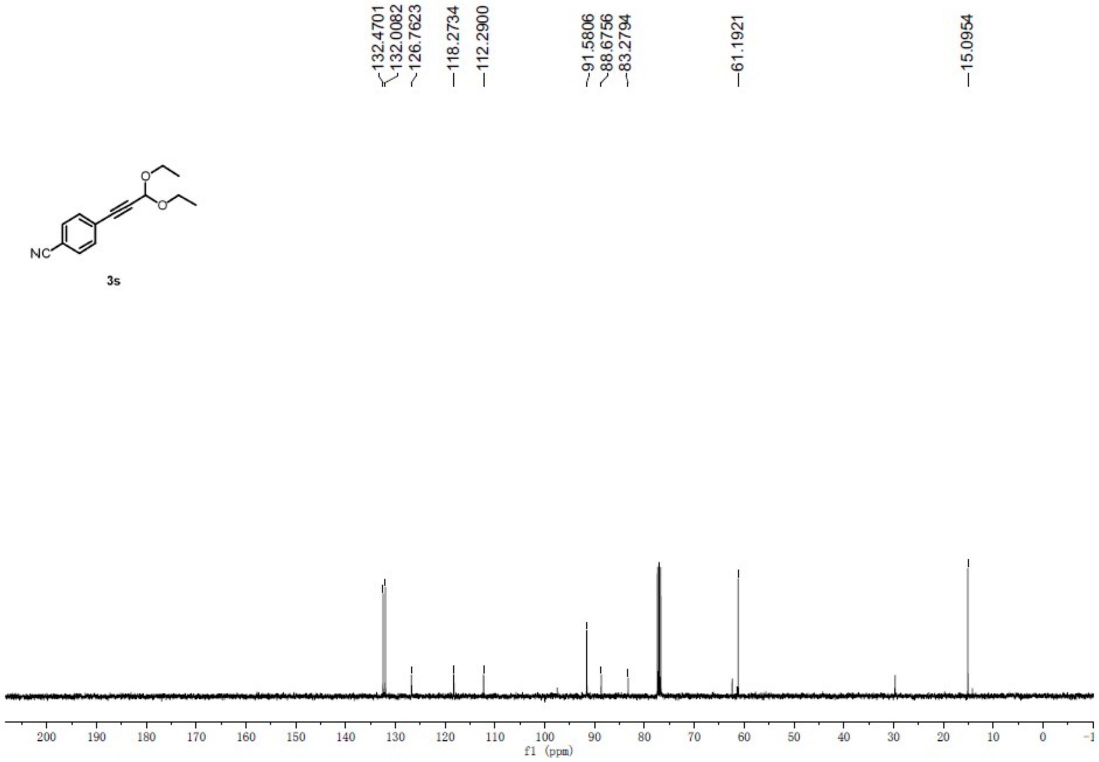
132.4701
132.0082
126.7623
118.2734
112.2900
91.5806
88.6756
83.2794
61.1921
15.0954
NC
O
O
3s
200 190 180 170 160 150 140 130 120 110 100 90 80 70 60 50 40 30 20 10 0 -1
f1 (ppm)

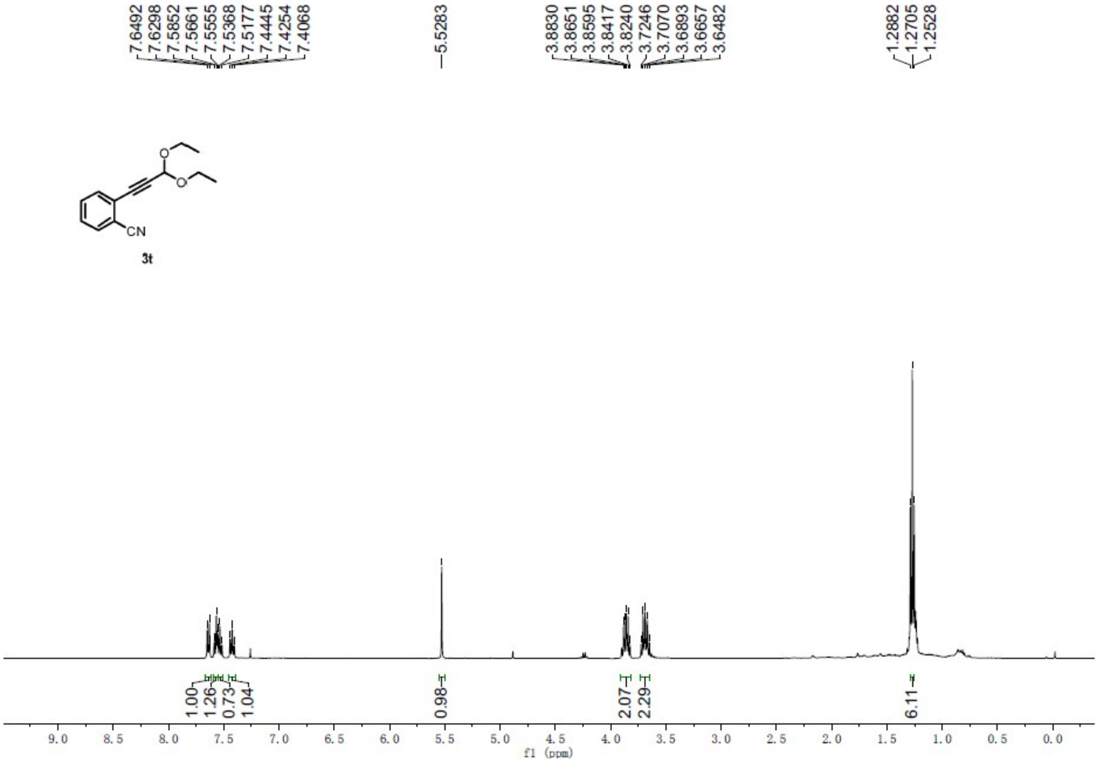
7.6492
7.6298
7.5852
7.5661
7.5555
7.5368
7.5177
7.4445
7.4254
7.4068
5.5283
3.8830
3.8651
3.8595
3.8417
3.8240
3.7246
3.7070
3.6893
3.6657
3.6482
1.2882
1.2705
1.2528
O
O
CN
3t
1.00
1.26
0.73
1.04
0.98
2.07
2.29
6.11
9.0 8.5 8.0 7.5 7.0 6.5 6.0 5.5 5.0 4.5 4.0 3.5 3.0 2.5 2.0 1.5 1.0 0.5 0.0
f1 (ppm)

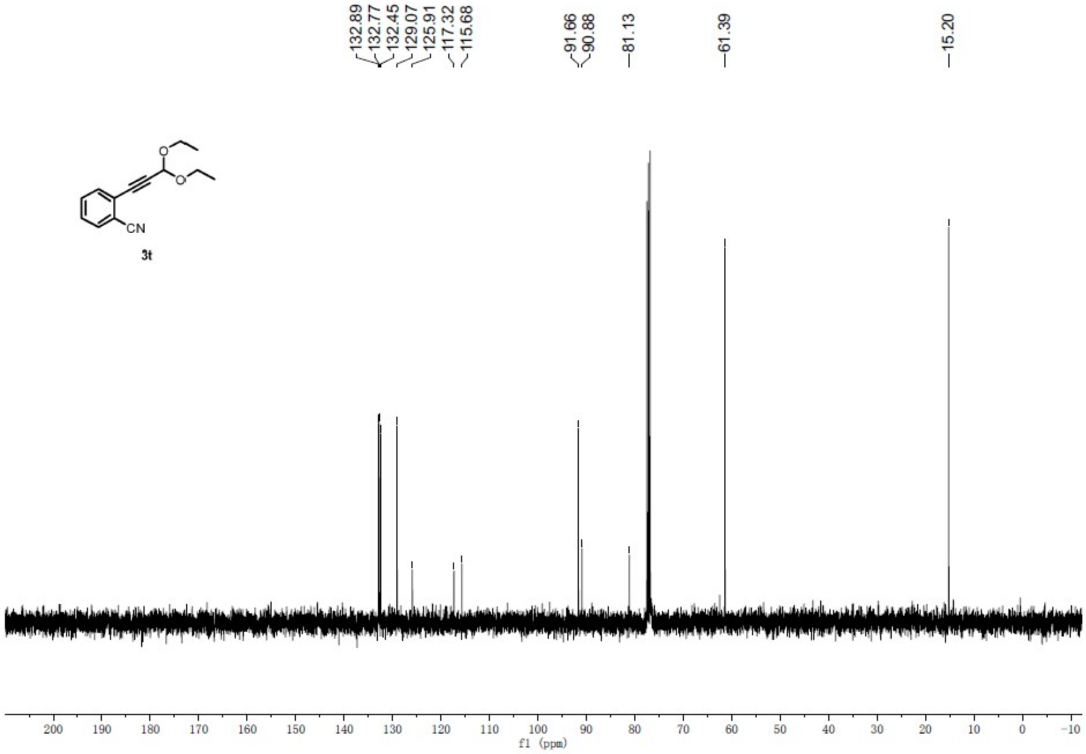
132.89
132.77
132.45
129.07
125.91
117.32
115.68
91.66
90.88
81.13
61.39
15.20
CN
3t
200 190 180 170 160 150 140 130 120 110 100 90 80 70 60 50 40 30 20 10 0 -10
f1 (ppm)

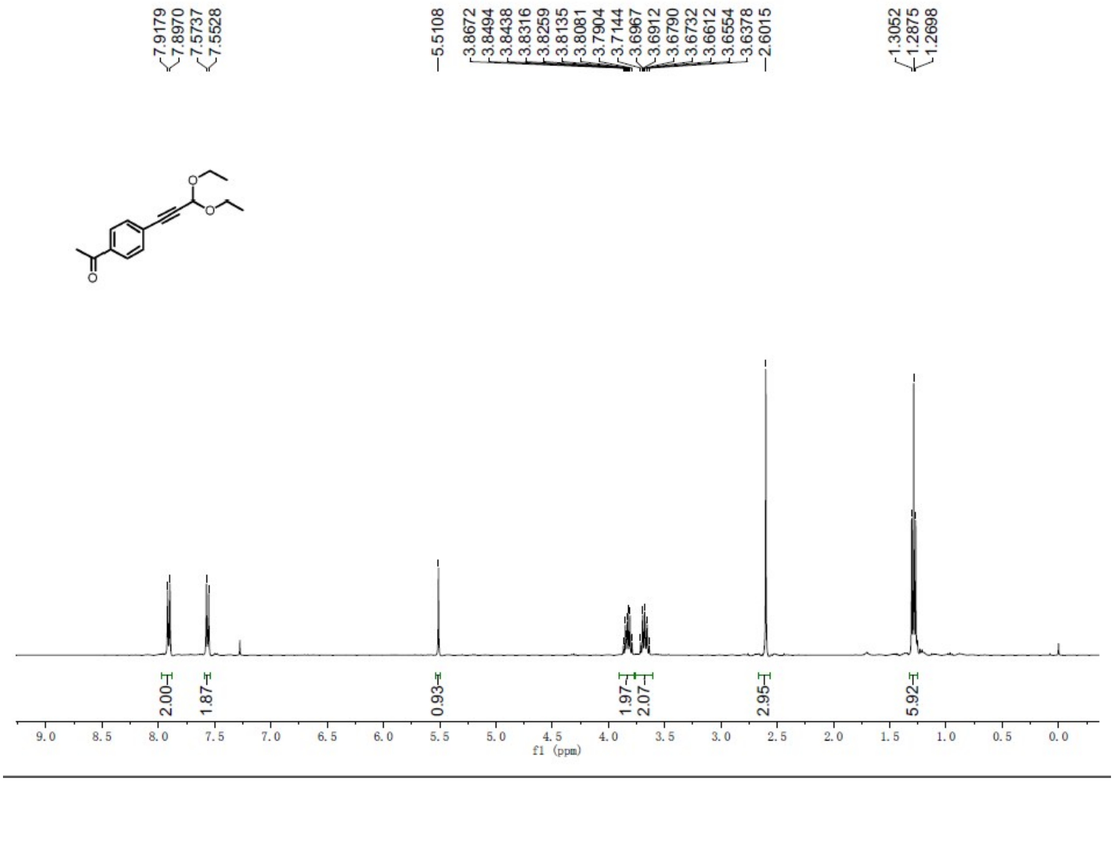
7.9179
7.8970
7.5737
7.5528
5.5108
3.8672
3.8494
3.8438
3.8316
3.8259
3.8135
3.8081
3.7904
3.7144
3.6967
3.6912
3.6790
3.6732
3.6612
3.6554
3.6378
2.6015
1.3052
1.2875
1.2698
O
2.00
1.87
0.93
1.97
2.07
2.95
5.92
9.0 8.5 8.0 7.5 7.0 6.5 6.0 5.5 5.0 4.5 4.0 3.5 3.0 2.5 2.0 1.5 1.0 0.5 0.0
f1 (ppm)

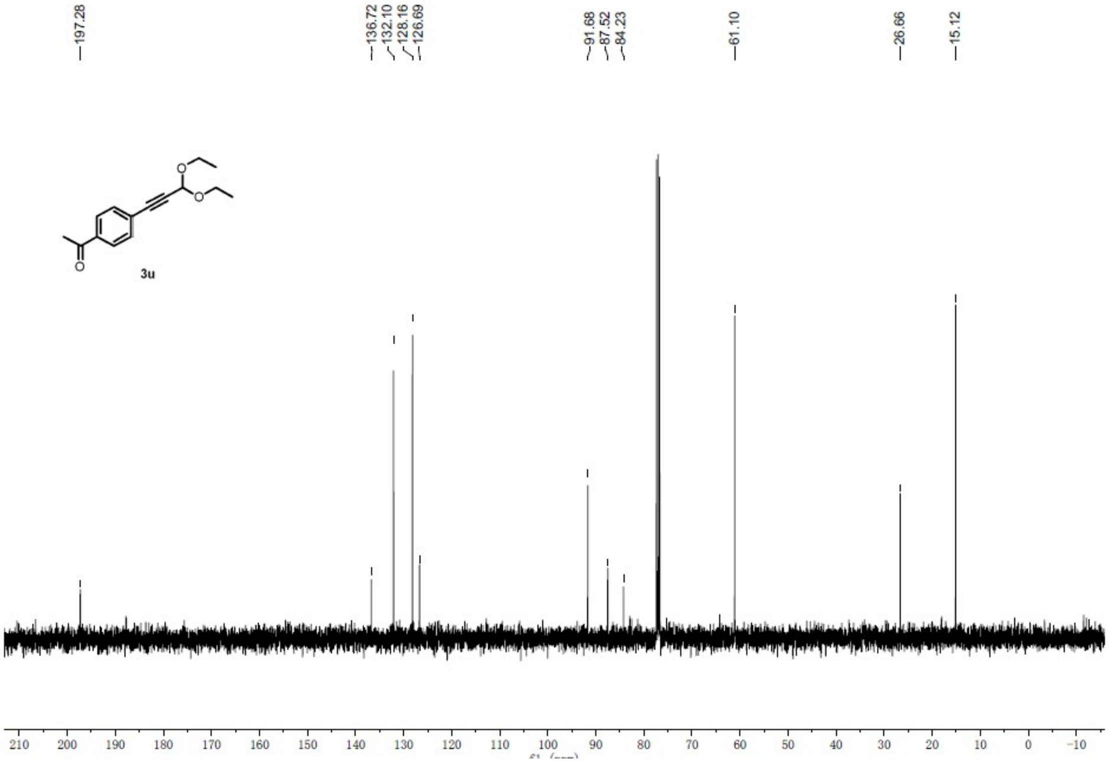

197.28
136.72
132.10
128.16
126.69
91.68
87.52
84.23
61.10
26.66
15.12
3u
210 200 190 180 170 160 150 140 130 120 110 100 90 80 70 60 50 40 30 20 10 0 -10

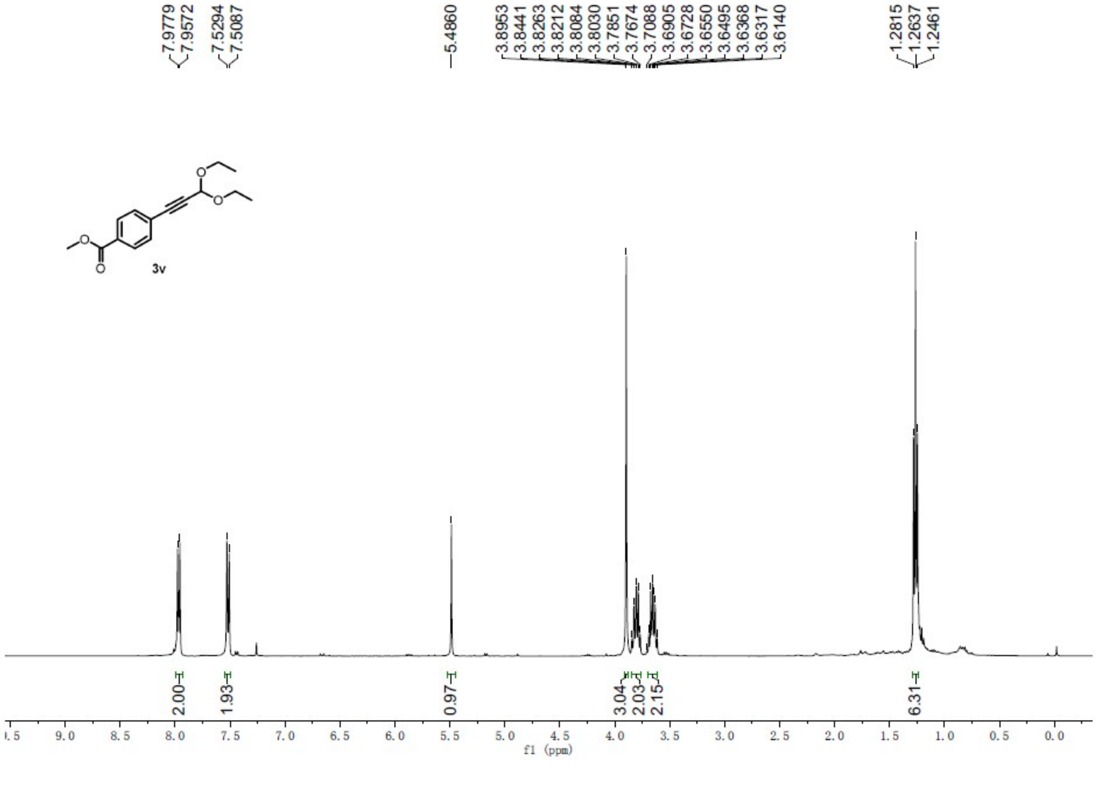

7.9779
7.9572
7.5294
7.5087
5.4860
3.8953
3.8441
3.8263
3.8212
3.8084
3.8030
3.7851
3.7674
3.7088
3.6905
3.6728
3.6550
3.6495
3.6368
3.6317
3.6140
1.2815
1.2637
1.2461
3v
2.00
1.93
0.97
3.04
2.03
2.15
6.31
9.0 8.5 8.0 7.5 7.0 6.5 6.0 5.5 5.0 4.5 4.0 3.5 3.0 2.5 2.0 1.5 1.0 0.5 0.0
f1 (ppm)

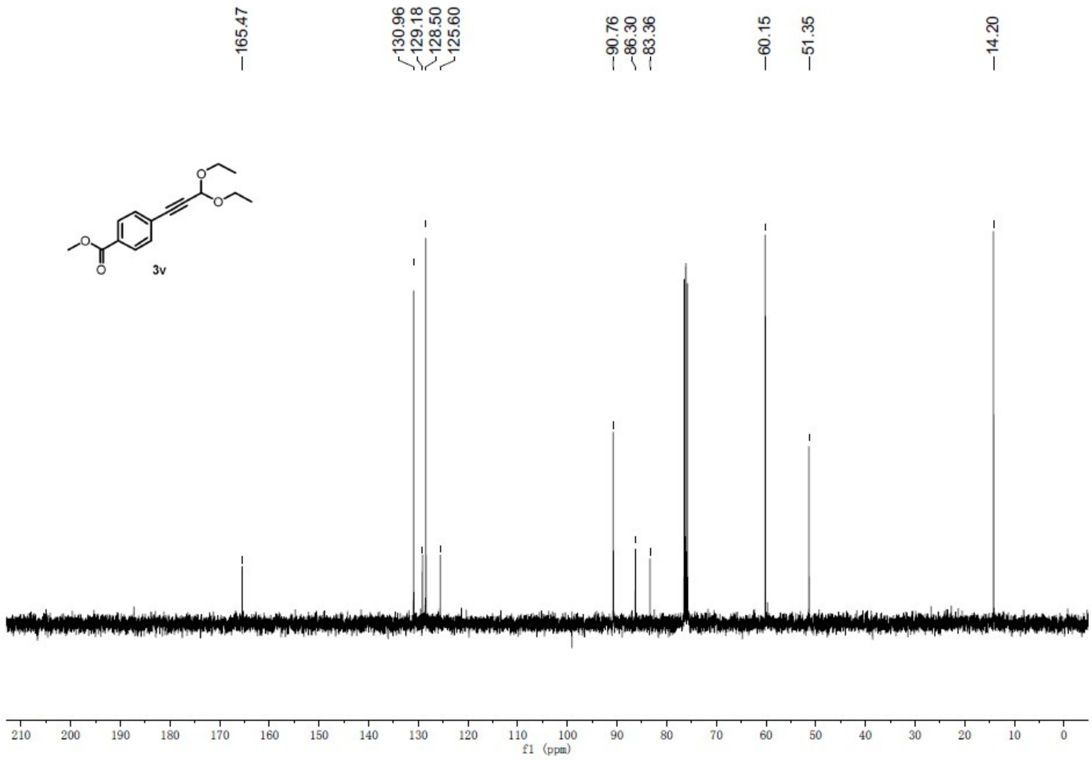

165.47
130.96
129.18
128.50
125.60
90.76
86.30
83.36
60.15
51.35
14.20
3v
210 200 190 180 170 160 150 140 130 120 110 100 90 80 70 60 50 40 30 20 10 0
f1 (ppm)

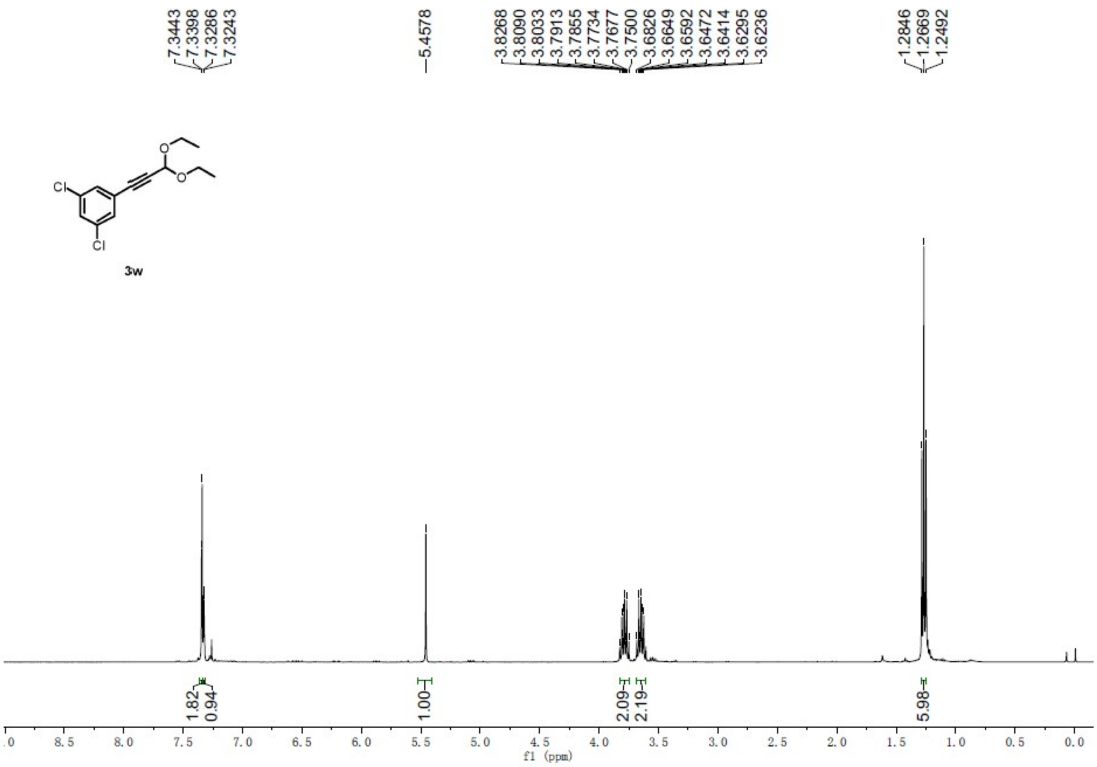
7.3443
7.3398
7.3286
7.3243
5.4578
3.8268
3.8090
3.8033
3.7913
3.7855
3.7734
3.7677
3.7500
3.6826
3.6649
3.6592
3.6472
3.6414
3.6295
3.6236
1.2846
1.2669
1.2492
Cl
O
Cl
3w
1.82
0.94
1.00
2.09
2.19
5.98
f1 (ppm)

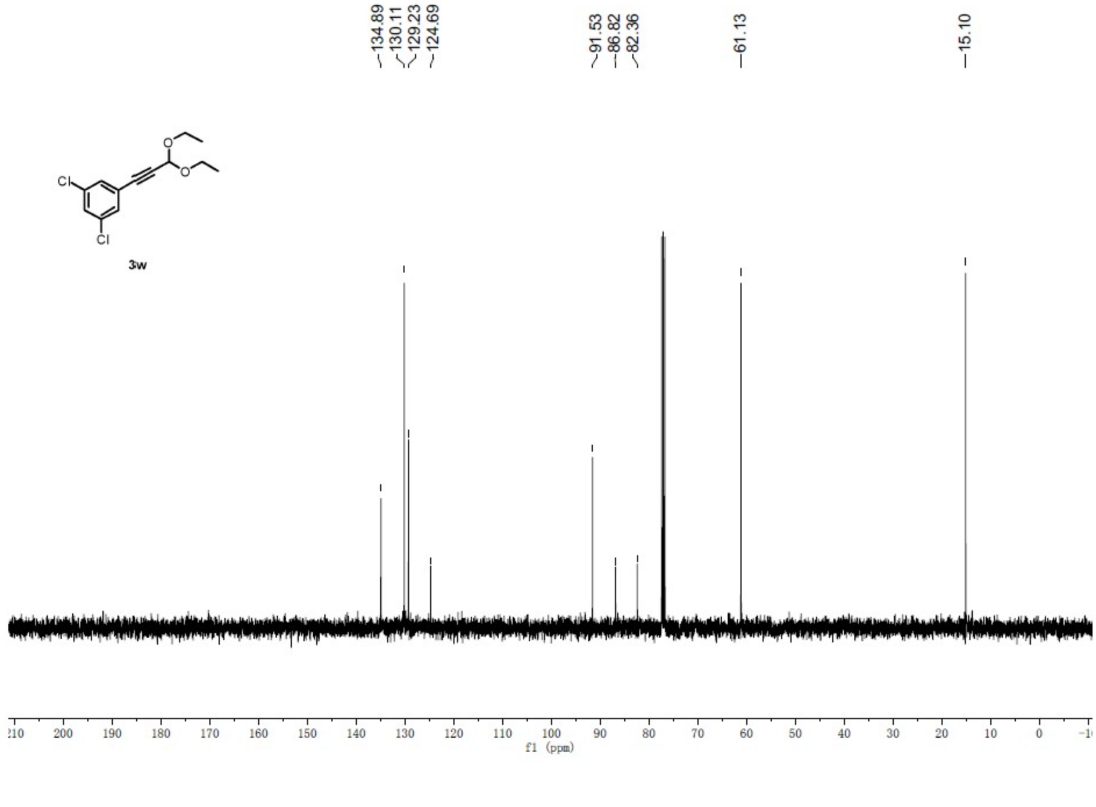
134.89
130.11
129.23
124.69
91.53
86.82
82.36
61.13
15.10
Cl
O
Cl
3w
f1 (ppm)

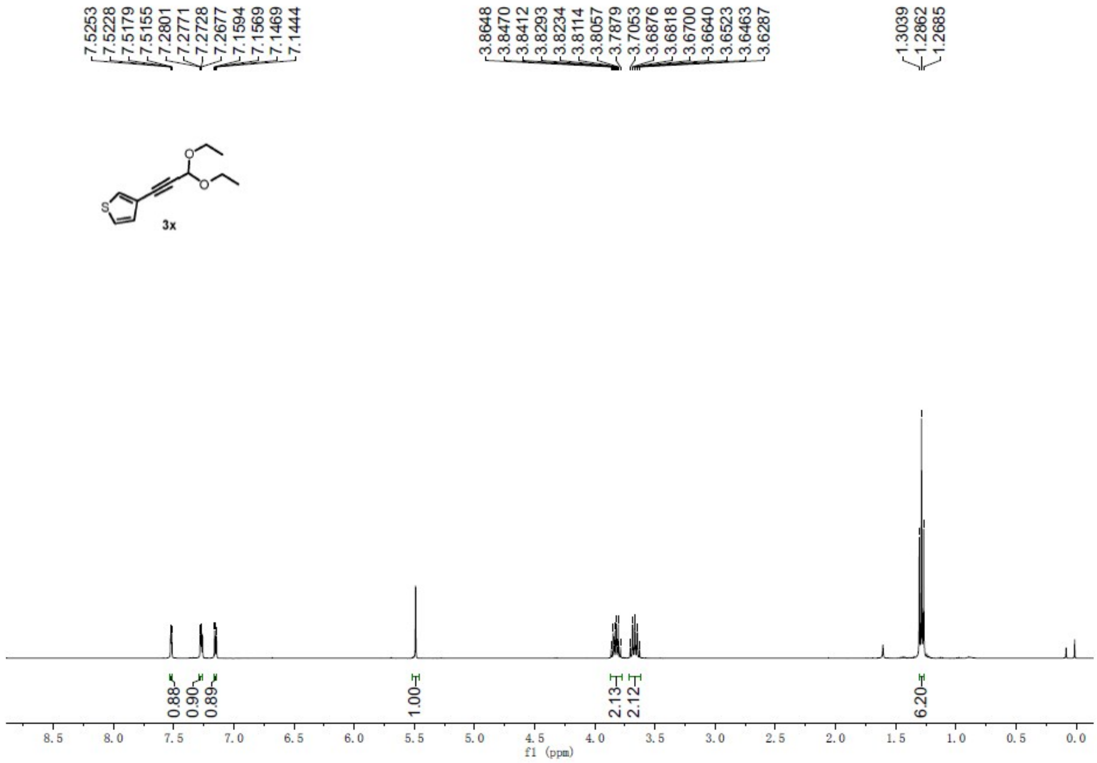
7.5253
7.5228
7.5179
7.5155
7.2801
7.2771
7.2728
7.2677
7.1594
7.1569
7.1469
7.1444
3.8648
3.8470
3.8412
3.8293
3.8234
3.8114
3.8057
3.7879
3.7053
3.6876
3.6818
3.6700
3.6640
3.6523
3.6463
3.6287
1.3039
1.2862
1.2685
S
O
O
3x
0.88
0.90
0.89
1.00
2.13
2.12
6.20
f1 (ppm)

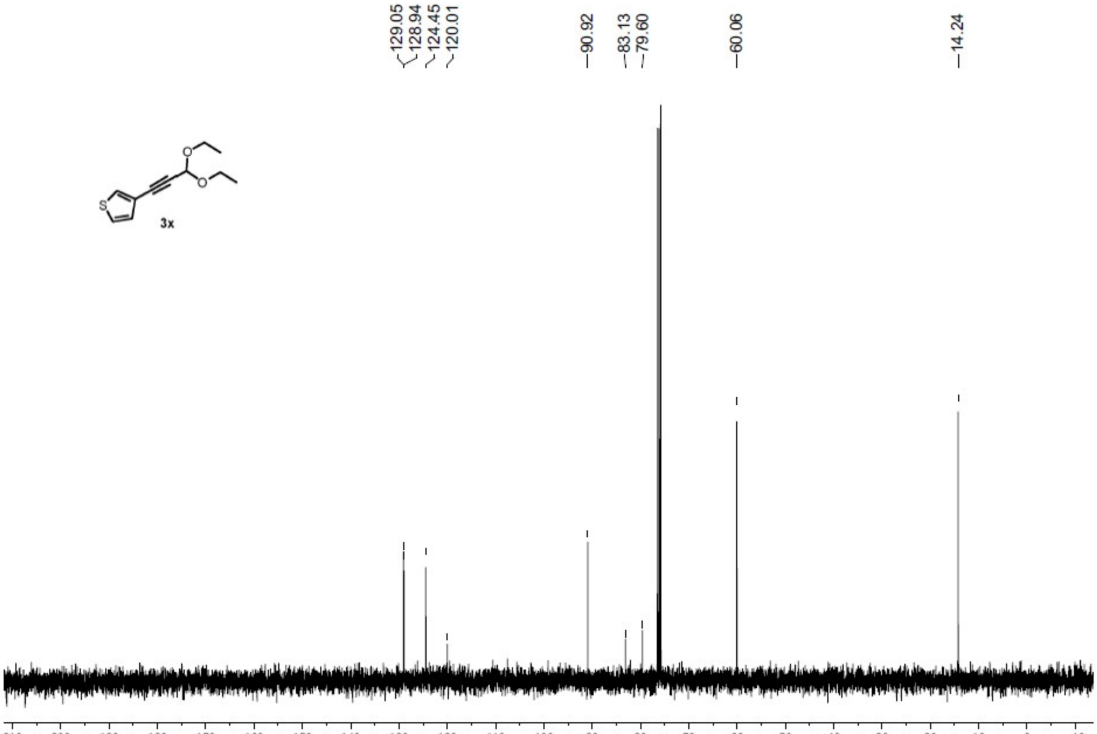
129.05
128.94
124.45
120.01
90.92
83.13
79.60
60.06
14.24
S
O
O
3x
f1 (ppm)

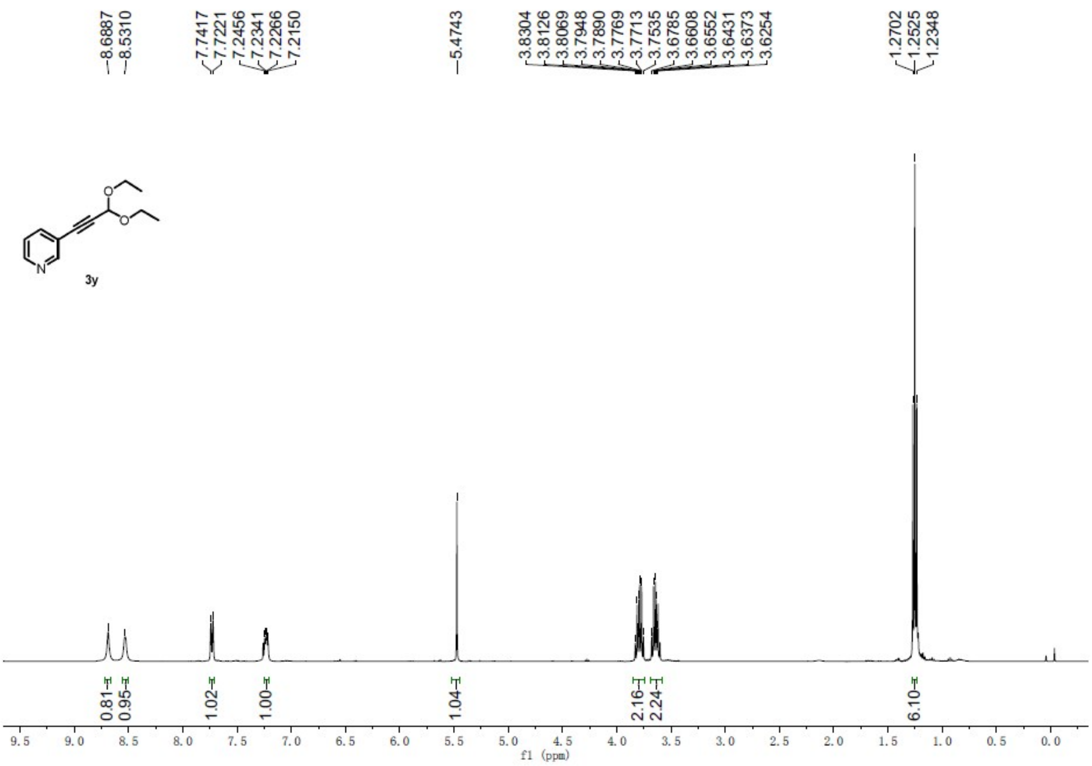
8.6887
8.5310
7.7417
7.7221
7.2456
7.2341
7.2266
7.2150
5.4743
3.8304
3.8126
3.8069
3.7948
3.7890
3.7769
3.7713
3.7535
3.6785
3.6608
3.6552
3.6431
3.6373
3.6254
1.2702
1.2525
1.2348
N
O
O
3y
0.81
0.95
1.02
1.00
1.04
2.16
2.24
6.10
9.5 9.0 8.5 8.0 7.5 7.0 6.5 6.0 5.5 5.0 4.5 4.0 3.5 3.0 2.5 2.0 1.5 1.0 0.5 0.0
f1 (ppm)

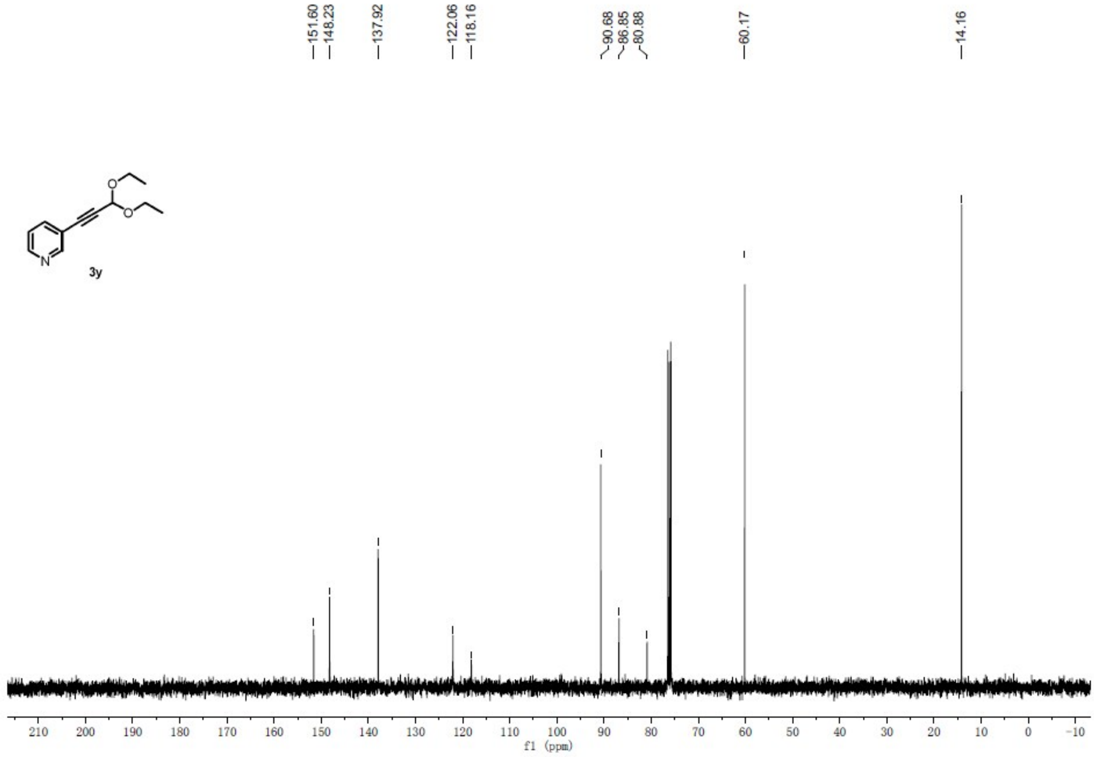
151.60
148.23
137.92
122.06
118.16
90.68
86.85
80.88
60.17
14.16
N
O
O
3y
210 200 190 180 170 160 150 140 130 120 110 100 90 80 70 60 50 40 30 20 10 0 -10
f1 (ppm)

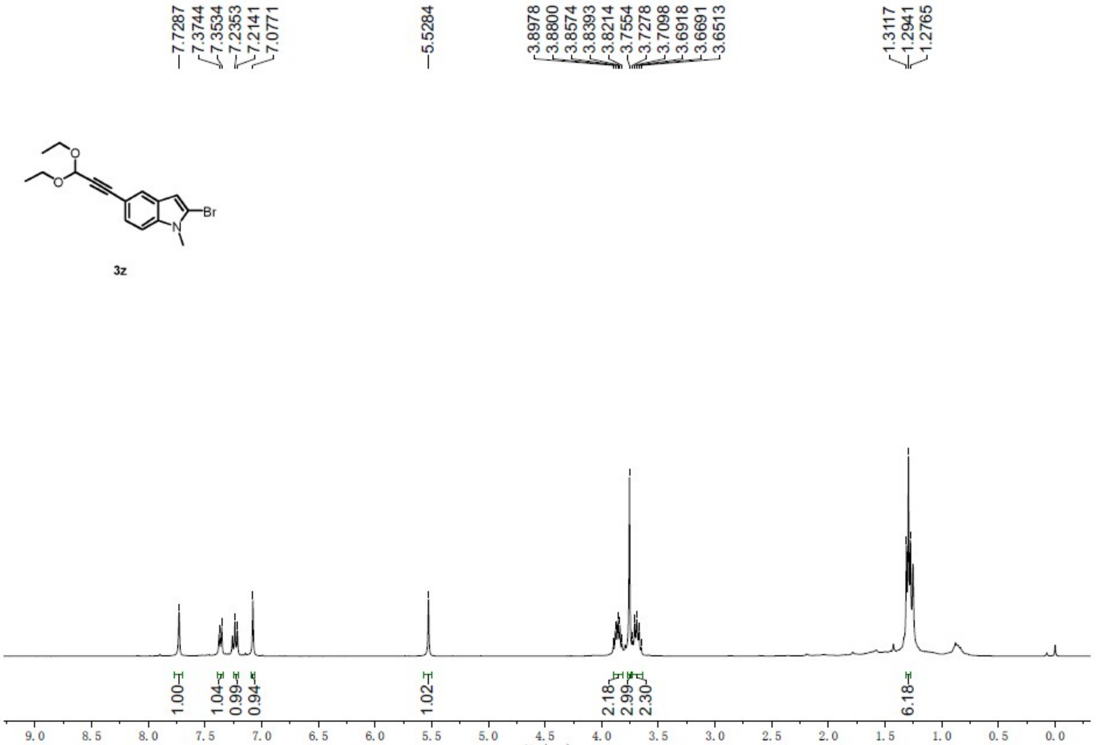
7.7287
7.3744
7.3534
7.2353
7.2141
7.0771
5.5284
3.8978
3.8800
3.8574
3.8393
3.8214
3.7554
3.7278
3.7098
3.6918
3.6691
3.6513
1.3117
1.2941
1.2765
Br
N
O
O
3z
1.00
1.04
0.99
0.94
1.02
2.18
2.99
2.30
6.18
9.0 8.5 8.0 7.5 7.0 6.5 6.0 5.5 5.0 4.5 4.0 3.5 3.0 2.5 2.0 1.5 1.0 0.5 0.0
f1 (ppm)

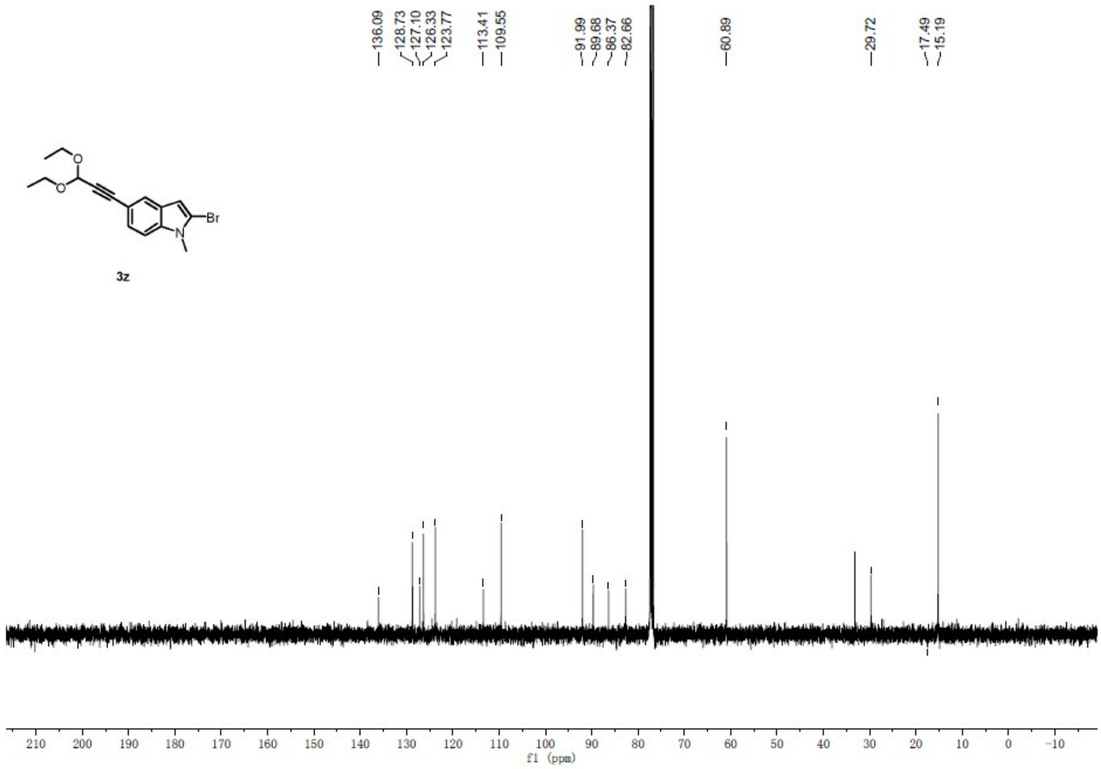
136.09
128.73
127.10
126.33
123.77
113.41
109.55
91.99
89.68
86.37
82.66
60.89
29.72
17.49
15.19
Br
N
O
O
3z
210 200 190 180 170 160 150 140 130 120 110 100 90 80 70 60 50 40 30 20 10 0 -10
f1 (ppm)

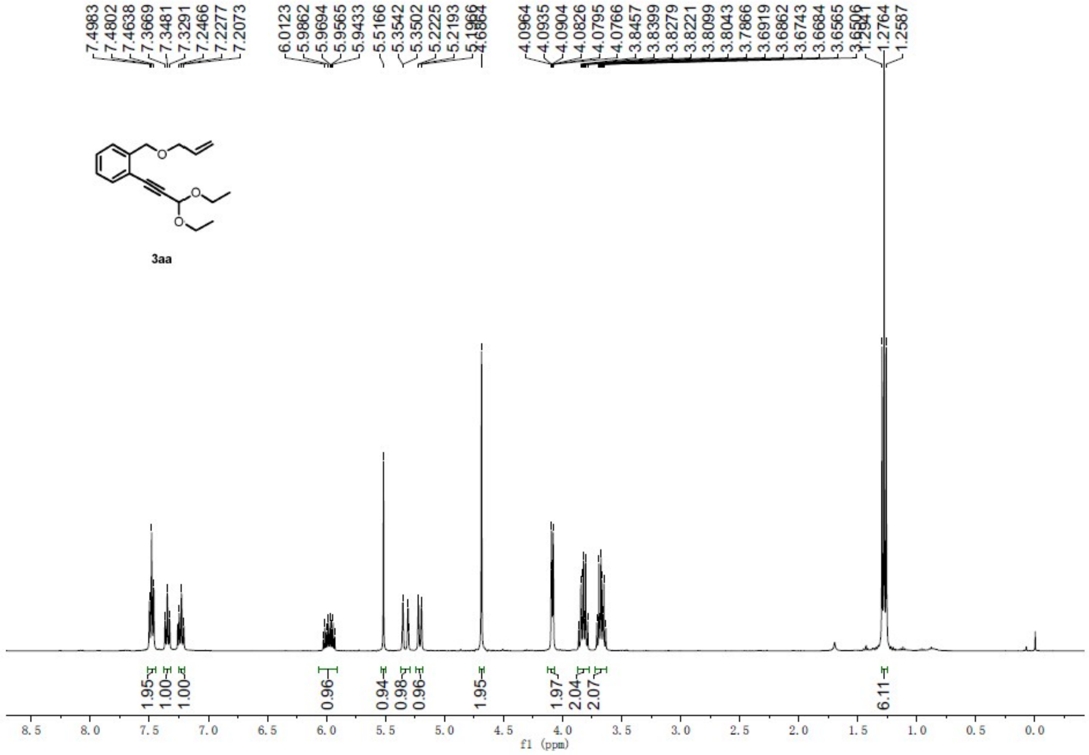
7.4983
7.4802
7.4638
7.3669
7.3481
7.3291
7.2466
7.2277
7.2073
6.0123
5.9862
5.9694
5.9565
5.9433
5.5166
5.3542
5.3502
5.2225
5.2193
4.6864
4.0964
4.0935
4.0904
4.0826
4.0795
4.0766
3.8457
3.8399
3.8279
3.8221
3.8099
3.8043
3.7866
3.6919
3.6862
3.6743
3.6684
3.6565
1.2764
1.2587
3aa
1.95
1.00
1.00
0.96
0.94
0.98
0.96
1.95
1.97
2.04
2.07
6.11
f1 (ppm)

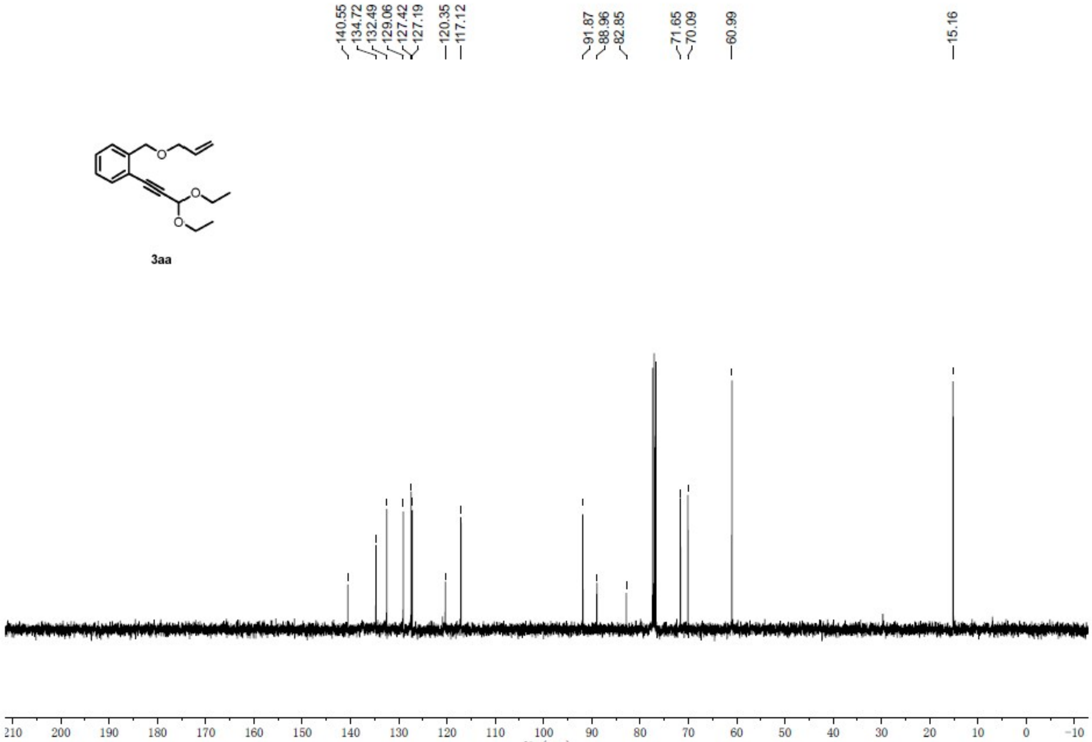
140.55
134.72
132.49
129.06
127.42
127.19
120.35
117.12
91.87
88.96
82.85
71.65
70.09
60.99
15.16
3aa
f1 (ppm)

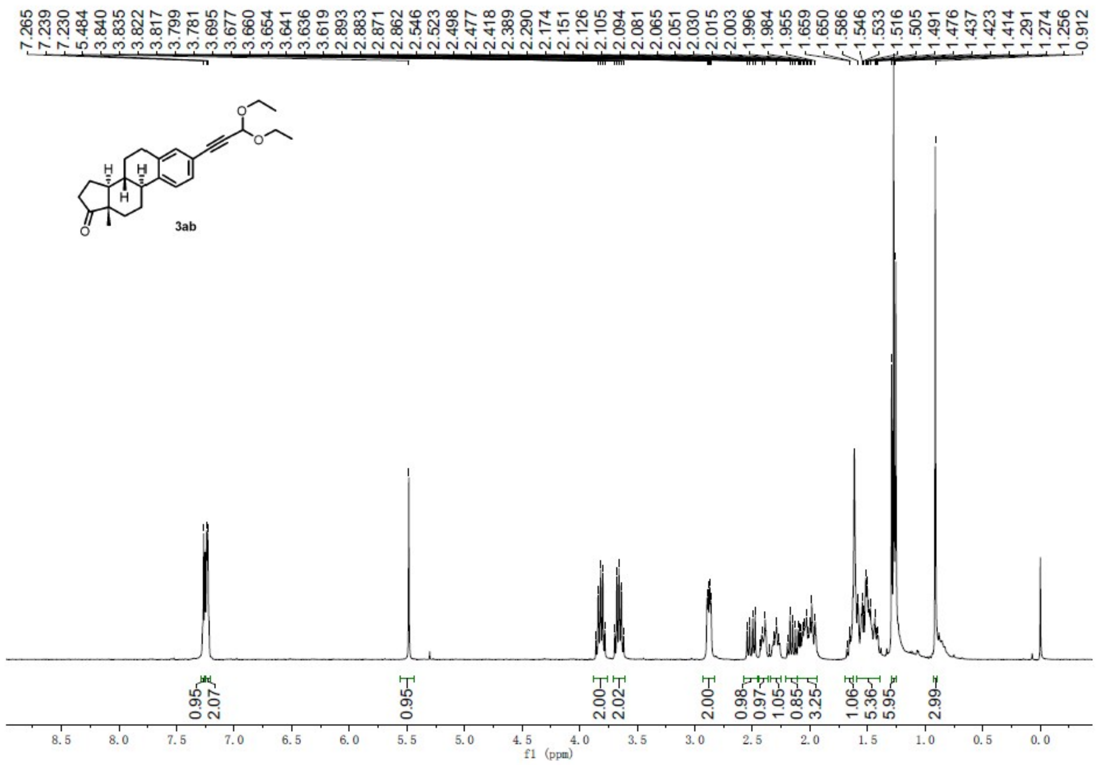

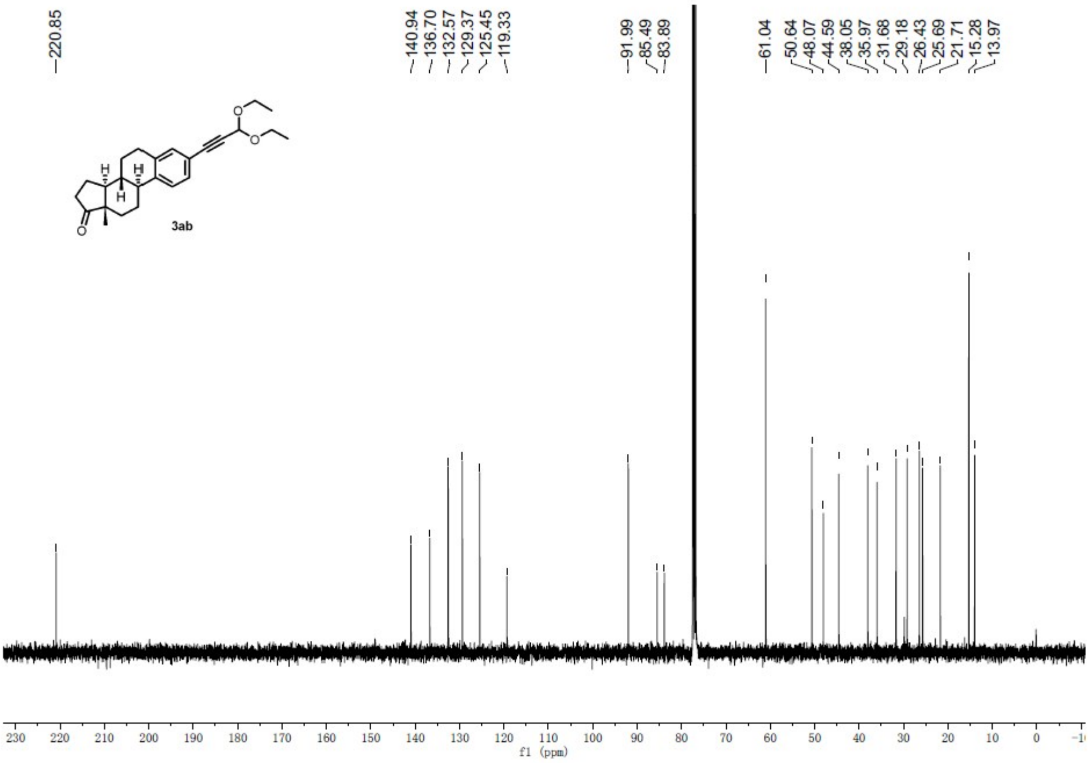
220.85
140.94
136.70
132.57
129.37
125.45
119.33
91.99
85.49
83.89
61.04
50.64
48.07
44.59
38.05
35.97
31.68
29.18
26.43
25.69
21.71
15.28
13.97
3ab
230 220 210 200 190 180 170 160 150 140 130 120 110 100 90 80 70 60 50 40 30 20 10 0
f1 (ppm)

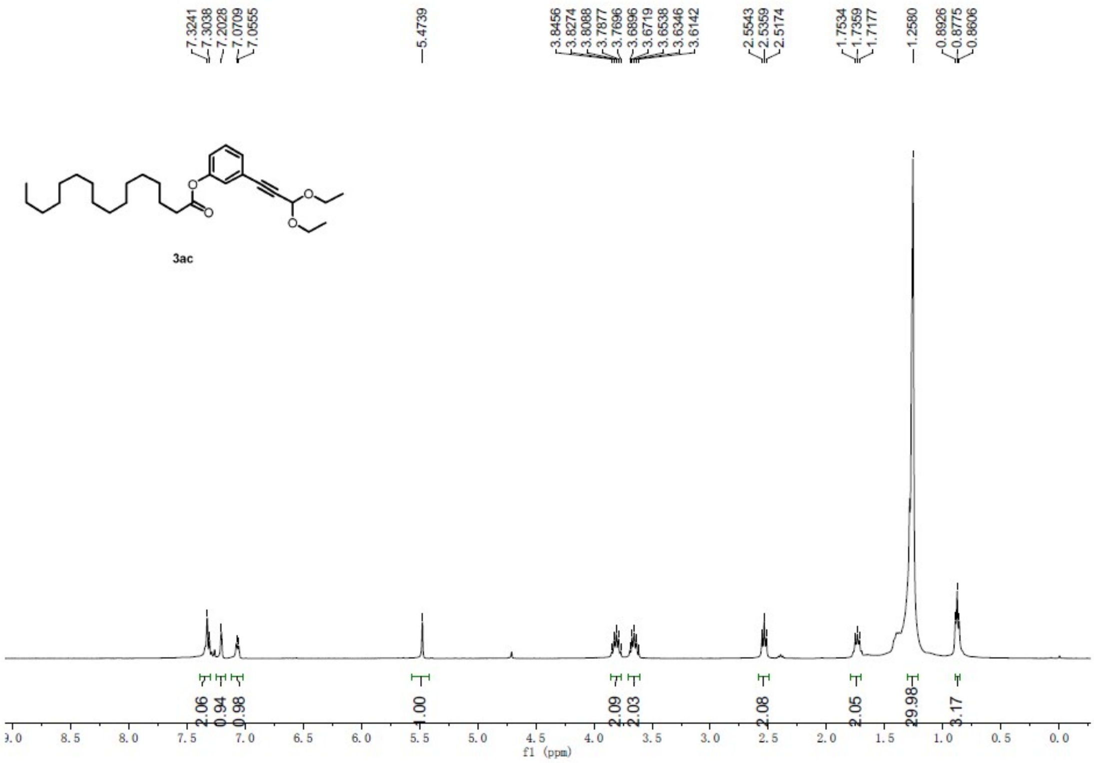
7.3241
7.3038
7.2028
7.0709
7.0555
5.4739
3.8456
3.8274
3.8088
3.7877
3.7696
3.6896
3.6719
3.6538
3.6346
3.6142
2.5543
2.5359
2.5174
1.7534
1.7359
1.7177
1.2580
0.8926
0.8775
0.8606
3ac
2.06
0.94
0.98
4.00
2.09
2.03
2.08
2.05
29.98
3.17
8.5 8.0 7.5 7.0 6.5 6.0 5.5 5.0 4.5 4.0 3.5 3.0 2.5 2.0 1.5 1.0 0.5 0.0
f1 (ppm)

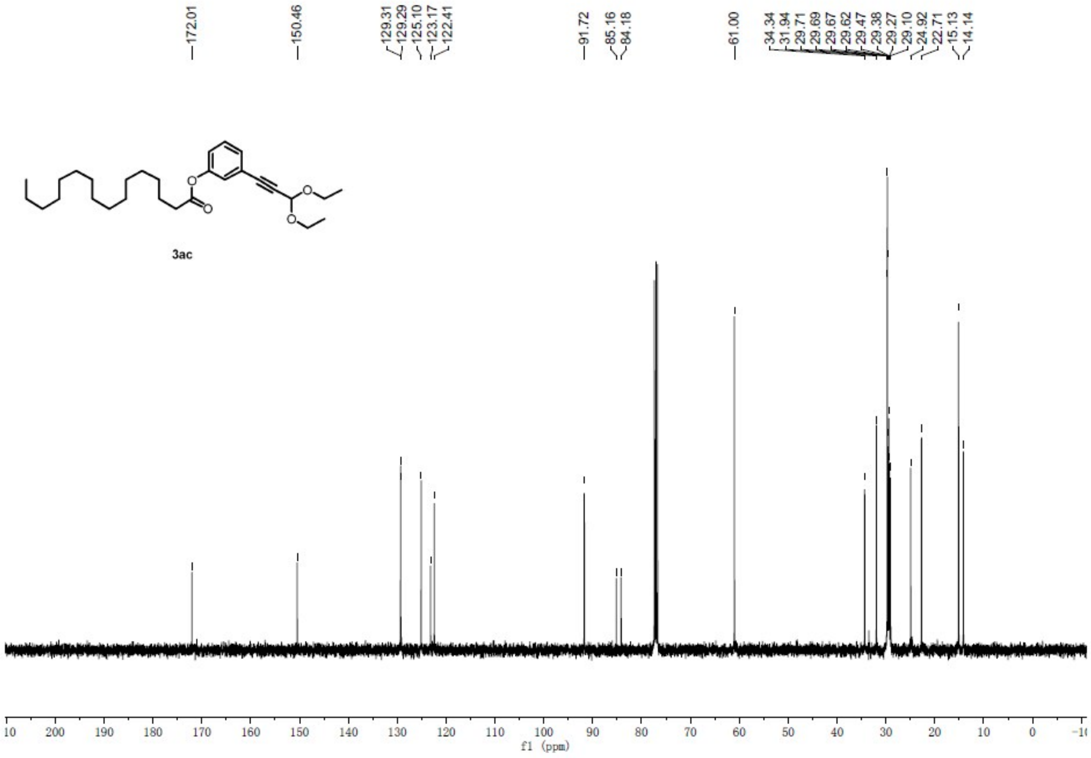

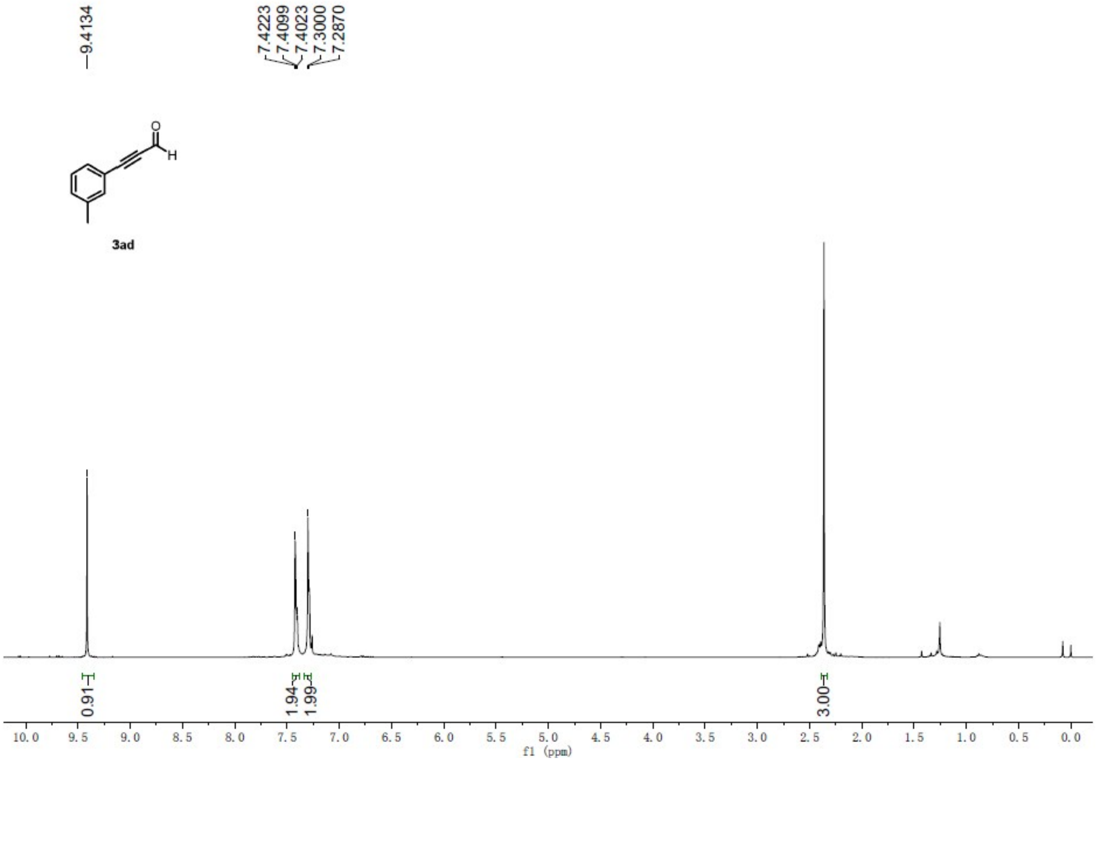

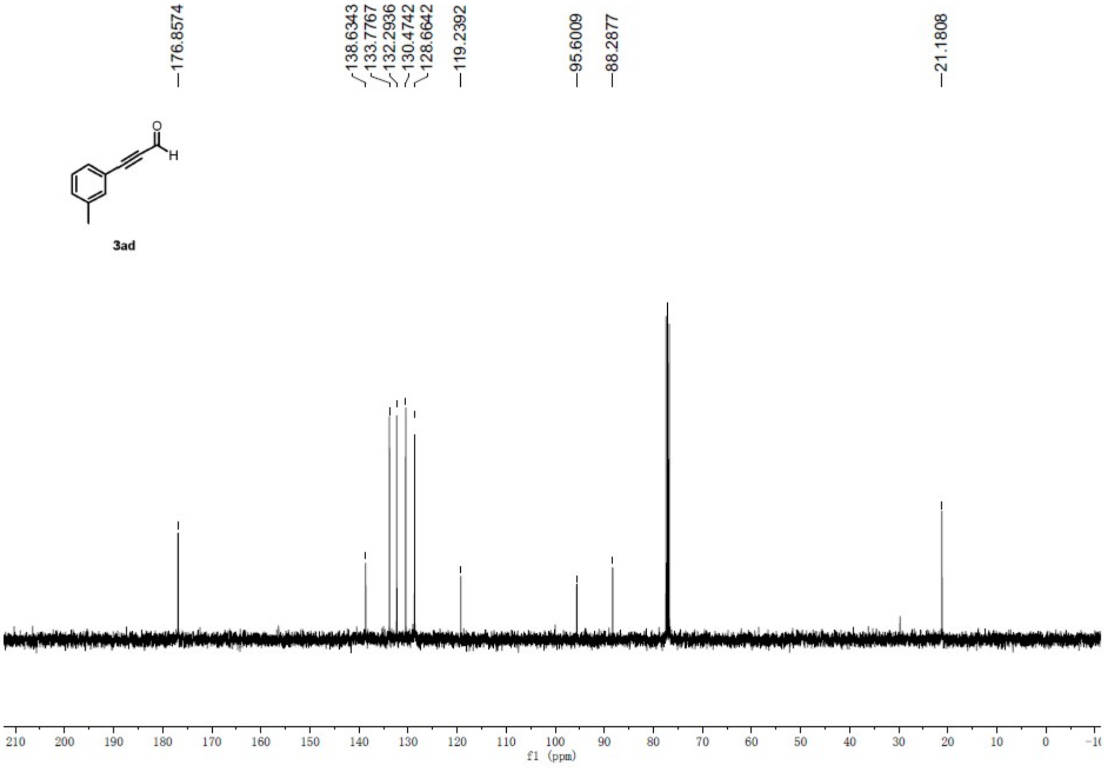
176.8574
138.6343
133.7767
132.2936
130.4742
128.6642
119.2392
95.6009
88.2877
21.1808
3ad
f1 (ppm)

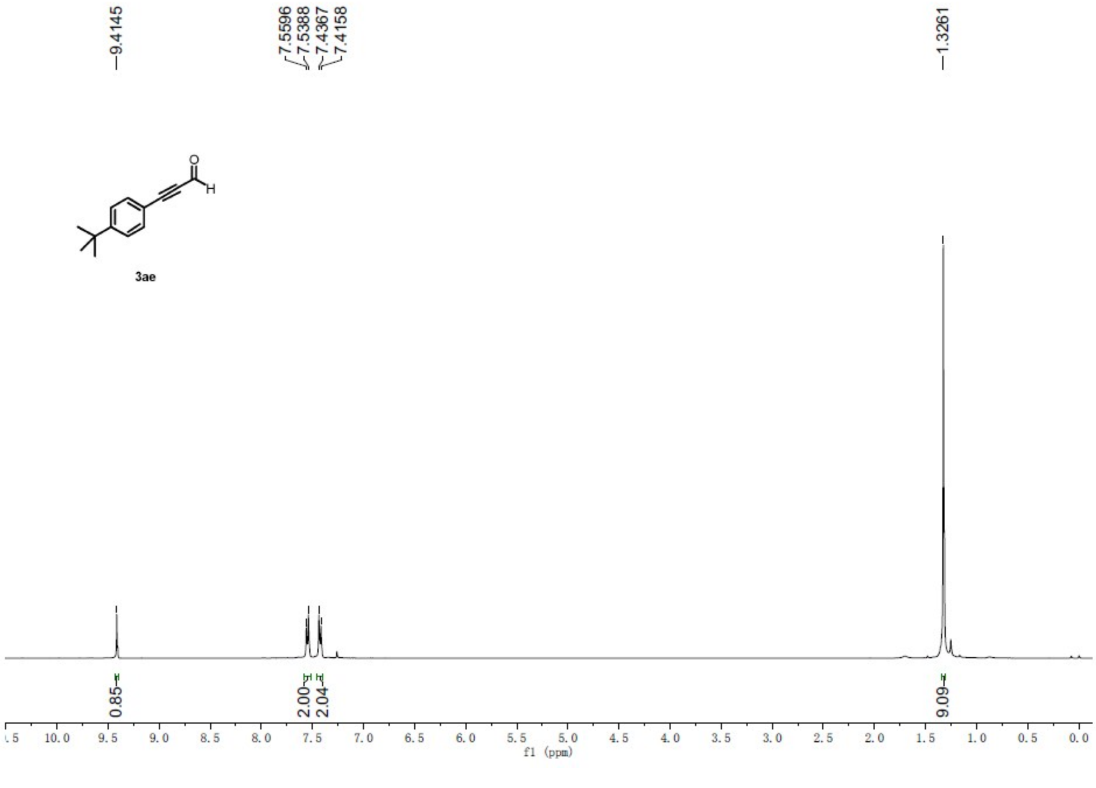
9.4145
7.5596
7.5388
7.4367
7.4158
1.3261
3ae
0.85
2.00
2.04
9.09
f1 (ppm)

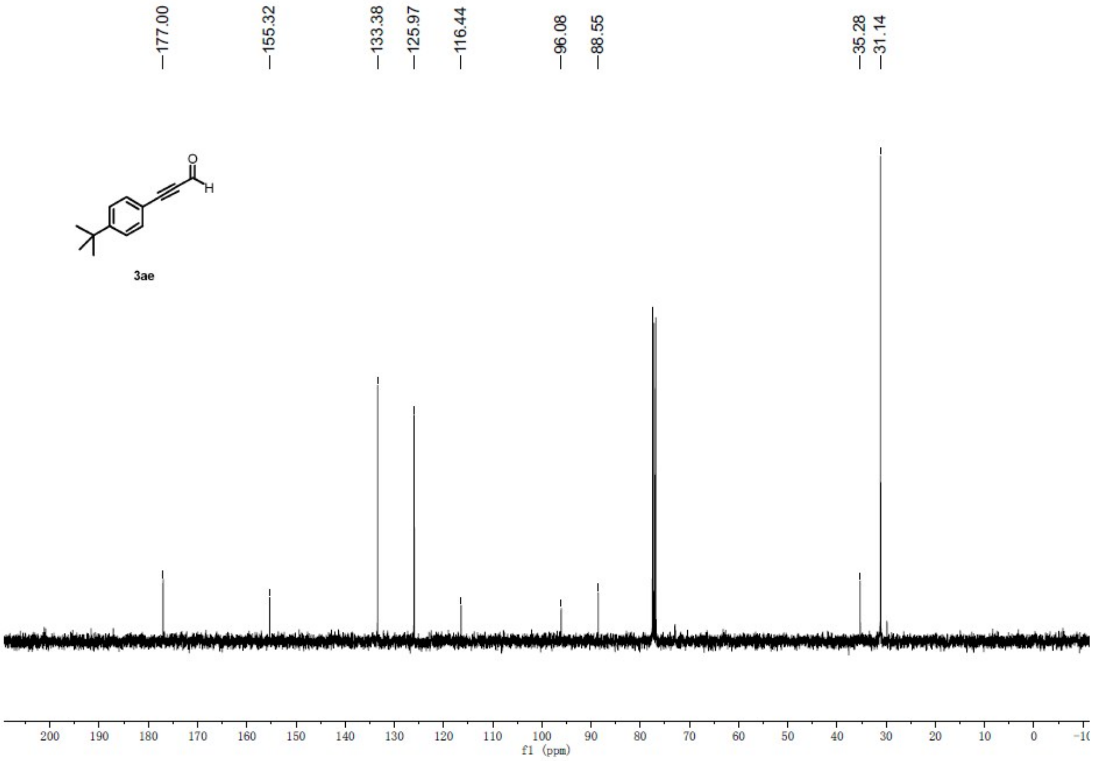

177.00
155.32
133.38
125.97
116.44
96.08
88.55
35.28
31.14
O
H
3ae
200 190 180 170 160 150 140 130 120 110 100 90 80 70 60 50 40 30 20 10 0
f1 (ppm)

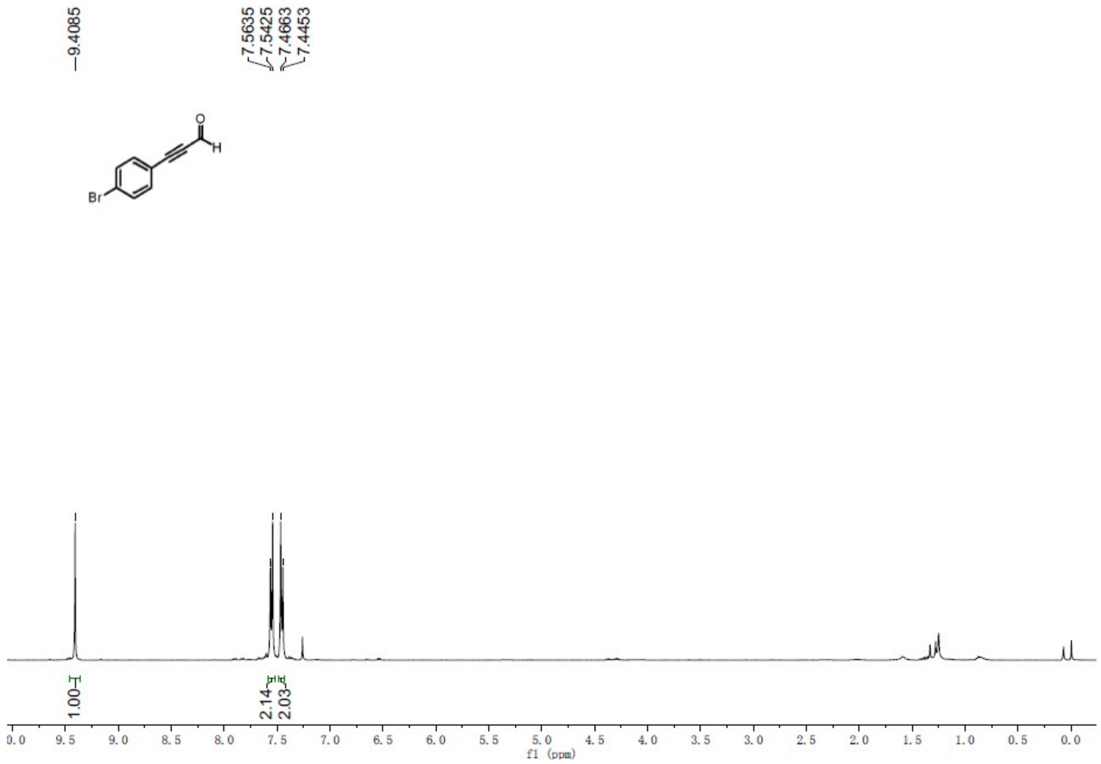

9.4085
7.5635
7.5425
7.4663
7.4453
Br
O
H
1.00
2.14
2.03
f1 (ppm)

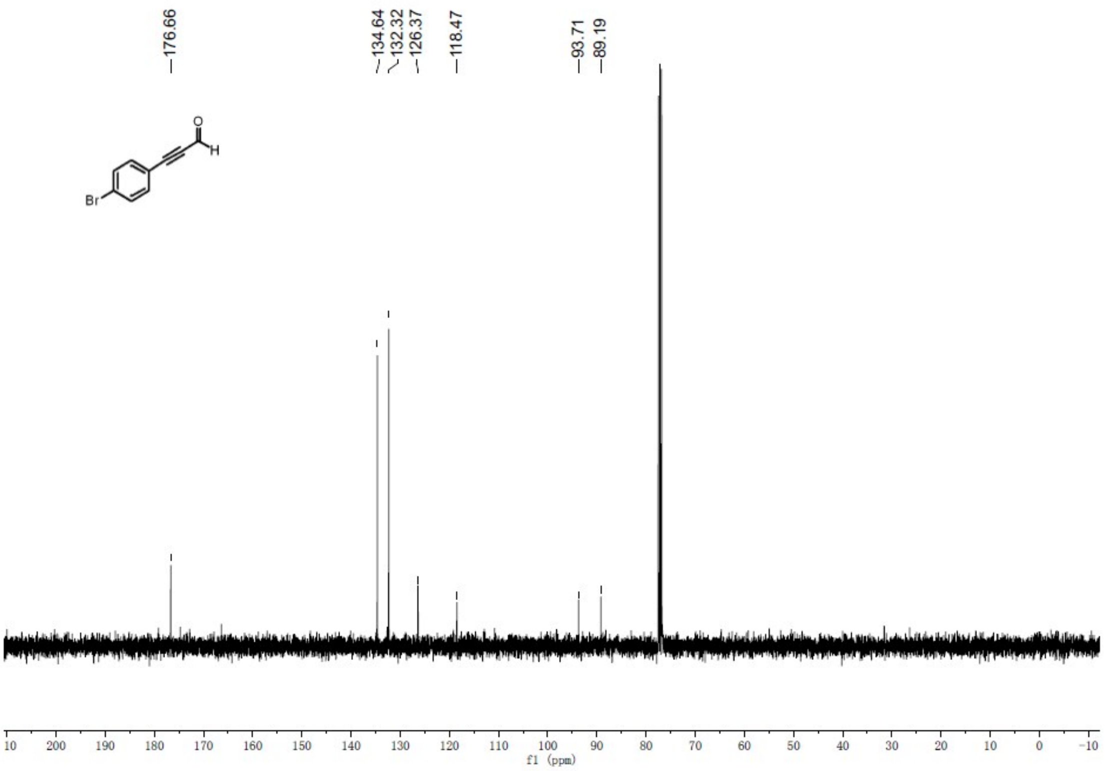

176.66
134.64
132.32
126.37
118.47
93.71
89.19
Br
O
H
f1 (ppm)
